# New Pyridobenothiazolone Derivatives Display Nanomolar Pan‐Serotype Anti‐Dengue Virus Activity

**DOI:** 10.1002/cmdc.202500163

**Published:** 2025-04-21

**Authors:** Tommaso Felicetti, Chin Piaw Gwee, Kitti Wing Ki Chan, Giacomo Pepe, Ciro Milite, Pietro Campiglia, Satoru Watanabe, Muhammad Danial Bin Mohd Mazlan, Stefano Sabatini, Serena Massari, Oriana Tabarrini, Gianluca Sbardella, Subhash G. Vasudevan, Giuseppe Manfroni

**Affiliations:** ^1^ Dipartimento di Scienze Farmaceutiche Università degli Studi di Perugia Via Del Liceo 1‐06123 Perugia Italy; ^2^ Program in Emerging Infectious Diseases Duke‐NUS Medical School Singapore 169857 Singapore; ^3^ Dipartimento di Farmacia Università degli Studi di Salerno Via Giovanni Paolo II, 132 84084 Fisciano Italy; ^4^ Institute for Biomedicine and Glycomics Griffith University Queensland 4222 Australia

**Keywords:** antiviral agents, dengue, flavivirus, medicinal chemistry, pyridobenzothiazolones

## Abstract

Dengue virus (DENV) serotypes 1–4 are mosquito‐borne flaviviruses that are responsible for significant morbidity and mortality worldwide, particularly in tropical and subtropical regions. Although two vaccines have been approved, their unbalanced efficacy across serotypes poses potential risks for specific populations. There are currently no approved antiviral treatments for DENV, resulting in a clear medical need, especially in endemic countries. Herein, a medicinal chemistry optimization of the pyridobenzothiazolone (PBTZ) derivative **2** is conducted, which results in the synthesis of a new series of PBTZ analogues. Compounds **15** and **19** exhibit nanomolar EC_50_ values against all four DENV serotypes. While new PBTZ analogues do not inhibit DENV polymerase as the first series of PBTZ analogues do, they display anti‐DENV activity across all time points during time‐of‐addition assays and demonstrate the capacity to influence the infectivity of newly produced virions without affecting viral RNA synthesis. Compound **19** exhibits an EC_50_ of 50 nM against DENV‐2 and a selectivity index of >2074, representing the most potent PBTZ analogue reported to date, with a significant improvement of over 30‐fold compared to the initial hit **2**. In vitro pharmacokinetic studies conducted on compound **19** disclose a promising profile, but with still some suboptimal values.

## Graphical abstract

A ligand‐based strategy is employed, starting from hit compound **2**, to develop the C‐2 modified pyridobenzothiazolone **19** as a pan‐serotype nanomolar DENV‐1‐4 inhibitor. Investigations of the mechanism reveal the peculiar ability to abolish the infectivity of virions in a second infection. In vitro pharmacokinetic studies conducted on **19** show promise, but not enough for an advancement to in vivo evaluation.

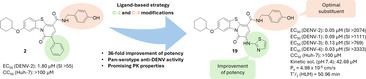

## 1 Introduction

The genus *Flavivirus* comprises ≈70 species of positive‐sense, single‐stranded RNA arthropod‐borne viruses (arboviruses).^[^ ^1^ ^]^ Of these, at least 30 are capable of causing disease in humans.^[^ ^2^ ^]^ Flaviviruses share common genomic organization and life cycles, as well as several host–pathogen protein interactions. Their transmission to a host (either human or animal) occurs through the bite or sting of vectors, including mosquitoes, flies, ticks, and fleas.^[^ ^3^ ^]^ Over the past few decades, the geographical distribution of Flaviviruses vectors has expanded due to climate change, leading to an increased incidence of infections.^[^ ^4^ ^]^ Of note, vector‐borne diseases account for ≈17% of all infectious diseases worldwide, and the World Health Organization (WHO) estimates that ≈80% of the global population is at risk of contracting one or more of these diseases.^[^ ^5^ ^]^ Among all Flaviviruses, six are of particular medical relevance: dengue (DENV), Zika (ZIKV), yellow fever (YFV), tick‐borne encephalitis (TBEV), Japanese encephalitis (JEV), and West Nile (WNV).^[^ ^4^ ^]^

DENV, the most rapidly spreading mosquito‐borne viral disease globally, represents a significant public health concern. DENV infection is distinguished by an increasing frequency of outbreaks, a rising mortality rate, and a broader geographical distribution across diverse regions of the world.^[^ ^6^ ^]^ According to WHO, DENV is endemic in over 100 countries and has recently emerged in new areas, including Europe, where it has caused serious outbreaks.^[^ ^7^ ^]^ Annually, WHO estimates that ≈100–400 million individuals are infected with DENV, with 500 000 hospitalizations and 20 000 deaths.^[^ ^8^ ^]^ The primary mode of transmission is through the bites of *Aedes aegypti* or *Aedes albopictus* mosquitoes.^[^ ^3^ ^]^ DENV exists in four distinct serotypes (DENV‐1–4), which exhibit close to 60–70% sequence similarity but are antigenically distinct and cause dengue diseases that share similar symptomatology.^[^ ^9^, ^10^ ^]^ Initial infection with any one of the DENV serotypes typically results in mild disease and provides lifelong immunity against that specific serotype. However, secondary infection with a different DENV serotype is associated with a heightened risk of severe illness due to a phenomenon known as antibody‐dependent enhancement.^[^ ^11^ ^]^ ≈25% of individuals infected with DENV exhibit symptoms, while severe cases leading to hospitalization occur in ≈1 out of 800 people. Mild, nonspecific symptoms such as fever, rash, and vomiting are common among patients. However, severe DENV infections can result in a syndrome known as hemorrhagic fever, which is characterized by hematemesis (vomiting blood) and fluid accumulation in the thorax or peritoneum.^[^ ^3^ ^]^ Without immediate fluid replacement therapy, this can progress to shock syndrome.^[^ ^12^ ^]^

Structurally, flaviviruses exhibit a similarity to the majority of enveloped virions, comprising a nucleocapsid that serves to protect the genome of single‐stranded RNA. This nucleocapsid is enveloped by a lipid bilayer containing membrane (M) and envelope (E) proteins.^[^ ^13^ ^]^ The genome encodes a single open reading frame (ORF), with 5′ and 3′ untranslated regions that have critical roles in translation and replication in conjunction with host and virus‐encoded proteins.^[^ ^13^ ^]^ The ORF is translated as a large polyprotein that is processed by viral and host proteases into three structural proteins (capsid, membrane, and envelope) and seven nonstructural proteins: NS1, NS2A, NS2B, NS3 (protease), NS4A, NS4B, and NS5 (polymerase). The nonstructural proteins play critical roles in viral RNA replication, modulation of the host immune response, and viral assembly.^[^ ^13^ ^]^

The absence of targeted antiviral therapies for DENV infections renders vaccination the most effective method for controlling the spread of the virus.^[^ ^14^ ^]^ However, despite the availability of two DENV vaccines, Dengvaxia and Qdenga, their limited protective efficacy against the DENV‐4 serotype and the potential risk of severe disease in naïve vaccines have hindered their widespread use.^[^ ^15^ ^]^ With respect to prospective therapeutic options, mosnodenvir (JNJ‐1802), an NS4B inhibitor with pan‐serotype anti‐DENV activity, entered Phase 2 clinical trials for the prevention of DENV infection in adults aged 16–65 years.^[^ ^16^ ^]^ However, at the conclusion of 2024, Johnson & Johnson made the decision to discontinue the study, a choice that was influenced by strategic reprioritization rather than safety concerns.^[^ ^17^ ^]^ Concurrently, a genomic surveillance study revealed that DENV‐2 and −3 lineages exhibited a low sensitivity to mosnodenvir, emerging multiple times over the years, thereby raising concerns about its efficacy on a large scale.^[^ ^18^ ^]^ Consequently, there is an immediate need to develop a therapy to treat DENV infections and to mitigate the risk of widespread epidemics.

**Figure 1:**
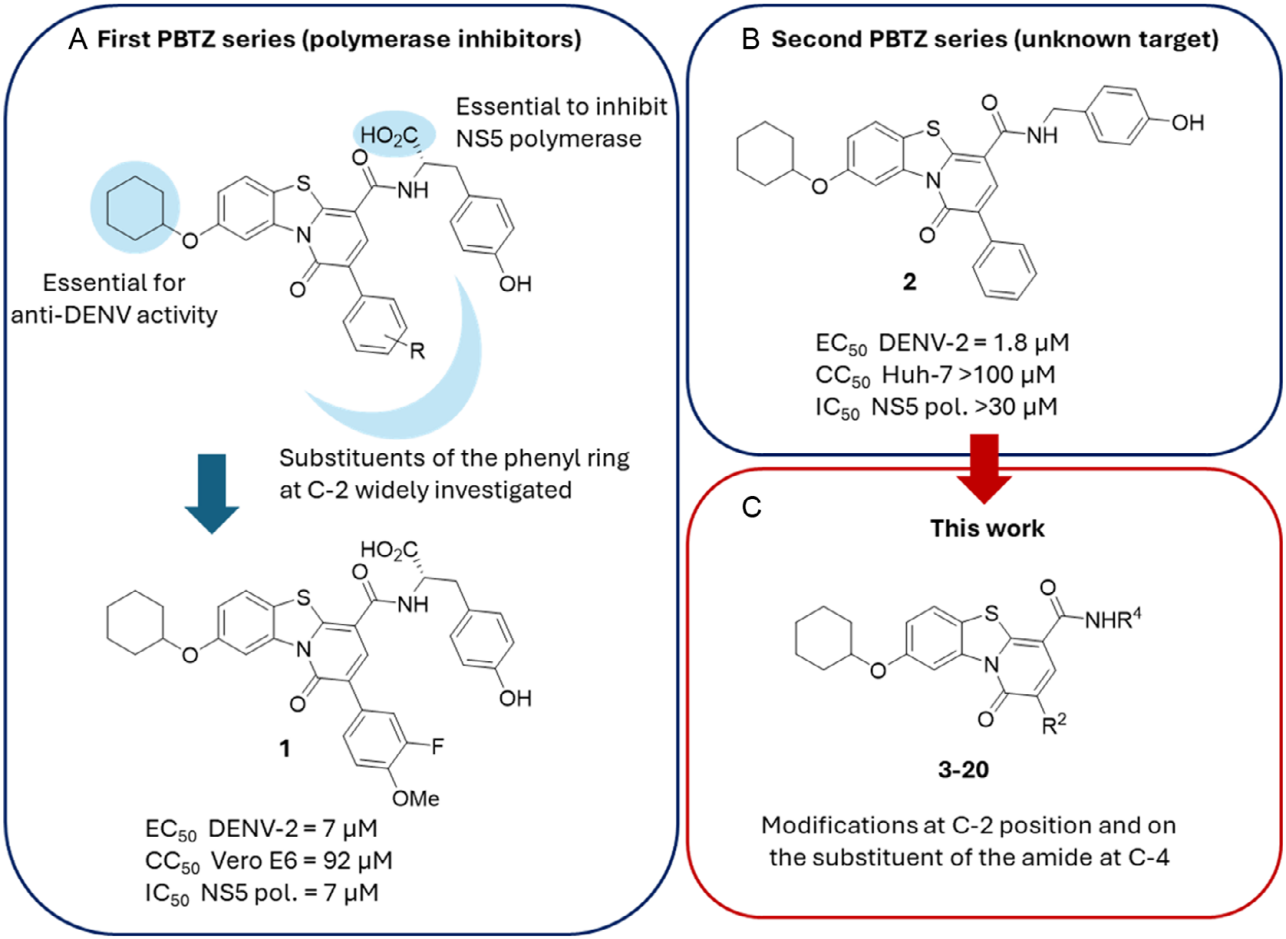
A) Chemical structures of PBTZ derivatives belonging to the first series, B) second series, and C) those developed in this work and a brief SAR summary around the known series A and B. A) PBTZ analogues belonging to the first series were developed as inhibitors of the dengue virus NS5 polymerase.^[^ ^19^ ^]^ Medicinal chemistry efforts resulted in the introduction of various modifications at the C‐2, C‐4, and C‐8 positions. Modifications at the C‐2 position primarily entailed the introduction of diverse substituents on the phenyl ring. In select instances, such as with **1**, this resulted in an enhancement of anti‐DENV activity. Modifications to the substituent of the amide at C‐4 included the introduction of different moieties, including aliphatic and aromatic groups. However, the primary conclusion was that the presence of a free carboxyl function was essential for polymerase inhibition. Modifications at the C‐8 position unambiguously demonstrated the critical role of the ether bridge and the cyclohexyl ring as a substituent. Replacements with alternative groups consistently resulted in derivatives with diminished or no activity.^[^ ^20^, ^21^ ^]^ B) The second series of PBTZ derivatives was more recent and less extensively investigated. Analogues belonging to this class are distinguished by the absence of polymerase inhibition, which renders them orphans of a known target.^[^ ^21^, ^22^ ^]^ The primary chemical modifications pertained to the substituent of the amide at C‐4, and these yielded some evidence. Despite the absence of the carboxyl group, it was demonstrated that a polar group capable of forming H bonds led to the formation of derivatives that effectively retained anti‐DENV activity. The introduction of alcohols resulted in an elevated level of toxicity. The removal of groups involved in H bonds resulted in the complete inactivation of the derivatives.^[^ ^21^, ^22^ ^]^ C) General chemical structure of the PBTZ derivatives designed and synthesized in this work. Chemical modifications were focused on the C‐2 position as well as on the substituent of the amide at C‐4.

In this direction, we have previously identified a series of pyridobenzothiazolone (PBTZ) analogues as broad‐spectrum antiflavivirus agents.^[^ ^19^, ^20^, ^21^, ^22^ ^]^ Most of the active PBTZ analogues were characterized by the presence of a cyclohexyloxy moiety at C‐8 position as well as a variously functionalized phenyl ring at C‐2 of the main tricyclic core. The main differences were observed when the C‐4 amide substituent was modified, which yielded two series of PBTZ analogues that both retained robust cell‐based antiflavivirus activity. The first series of compounds featured an aminoacidic substituent at the C‐4 position with a free carboxylic function and inhibited the NS5 polymerase (derivative **1** was included in **Figure** **1**A as a representative example).^[^ ^19^, ^20^ ^]^ In contrast, the second and more recent series was devoid of carboxylic functionality and demonstrated no inhibitory activity against NS5 polymerase despite retaining noteworthy anti‐DENV activity (Figure 1B).^[^ ^21^, ^22^ ^]^ Derivative **2** ^[^ ^21^ ^]^ (Figure 1B and **Table** **1**), belonging to the second PBTZ series, represents one of the most potent PBTZ anti‐DENV analogues. Consequently, it was selected as the starting hit for further investigation. Given the lack of understanding regarding the mechanism of action, a ligand‐based drug design strategy was employed. Herein, we present the design and synthesis of a series of C‐4 and C‐2 functionalized PBTZ amide derivatives **3–20** (Figure 1C and Table 1). The results of these efforts led to the identification of some potent antiflavivirus agents with nanomolar EC_50_ values.

**Table 1:** Anti‐DENV‐2 activity and cytotoxicity on Huh‐7 cells of PBTZ analogues **3**–**20**.

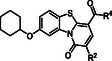					
Code	R^2^	R^4^	EDEN2 EC_50_ [μM]	Huh‐7 CC_50_ [μM]	SI
**2** ^[^ ^21^ ^]^	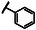	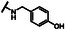	1.80 ± 0.08	>100	>50
**3**	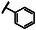	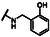	0.70 ± 0.18	>100	>143
**4**	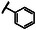	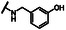	2.22 ± 0.51	67.2	30
**5**	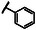	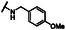	NIa)	NDb)	NDb)
**6**	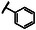	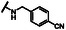	0.54 ± 0.04	>100	>184
**7**	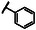	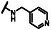	1.14 ± 0.41	>100	>88
**8**	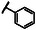	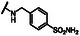	0.71 ± 0.19	40.6	57
**9**	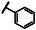	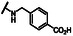	0.32 ± 0.07	52.6	166
**10**	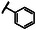	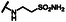	0.39 ± 0.20	32.9	84
**11**	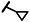	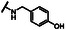	1.40 ± 0.31	87.6	62
**12**	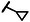	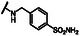	1.10 ± 0.18	78.8	71
**13**	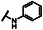	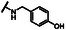	0.45 ± 0.12	98.5	219
**14**	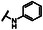	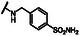	1.64 ± 0.74	93.0	57
**15**	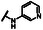	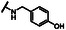	0.14 ± 0.05	>100	>700
**16**	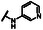	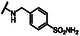	0.28 ± 0.08	>100	>351
**17**	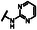	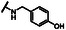	0.11 ± 0.02	>100	>872
**18**	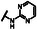	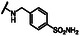	0.10 ± 0.02	49.5	476
**19**	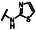	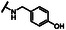	0.05 ± 0.01	>100	>2074
**20**	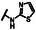	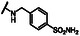	0.19 ± 0.07	95.9	494

Compounds were initially tested at 10 μM against DENV‐2‐infected Huh‐7 cells. Subsequently, those compounds that were able to completely inhibit viral growth at 10 μM were subjected to a dose–response testing from 0.001 to 50 μM in order to calculate the effective concentration that results in 50% reduction in infective virus particles (EC_50_) for DENV‐2. In parallel, toxicity was determined on Huh‐7 cells to obtain CC_50_ values, and the SI (CC_50_/EC_50_) was calculated. The antiviral efficacy is presented as average with standard deviation from two independent experiments.

a) NI: no inhibition as the compound exhibited cellular toxicity at 10 μM.

b) ND: not determined.

## 2 Results and Discussion

### 2.1 Chemistry

**Scheme 1:**
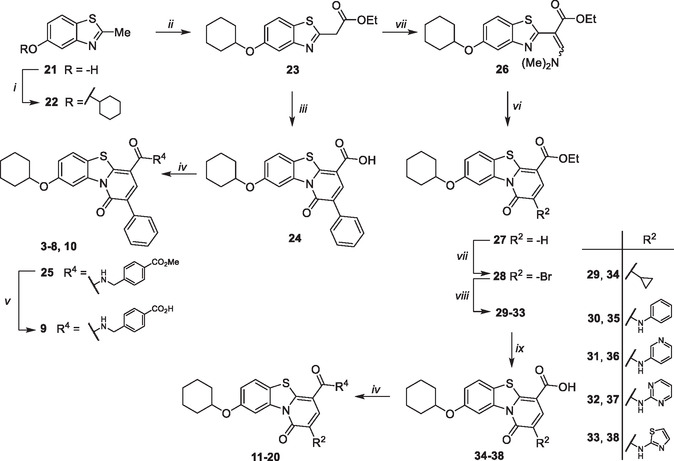
See Table 1 for chemical structures of derivatives **3‐20**. Reagents and conditions: i) cyclohexyl *p*‐toluenesulfonate, Cs_2_CO_3_, dry DMF, 100 °C, 4 h; ii) 60% NaH, CO(OEt)_2_, dry THF, reflux, 8 h; iii) DMF‐DMA, phenylacetic anhydride, 110 °C, 2 h, then 10% aq. NaOH, EtOH, 80 °C, 2 h; iv) appropriate amine, BOP, Et_3_N, rt, 1–12 h, or appropriate amine, EDCI·HCl, HOBt·H_2_O, DIPEA, rt, 2–48 h; v) 1N aq. LiOH, dioxane, rt, 16 h; vi) DMF‐DMA, dry DMF, 80 °C, 2 h; vii) Br_2_, AcOH, rt, 30 min; viii) appropriate amine, BINAP or Xantphos, Pd(OAc)_2_ or Pd_2_(dba)_3_, K_2_CO_3_ or Cs_2_CO_3_ or NaO*t*Bu, dry toluene, reflux, 1–24 h, or (for **29**) cyclopropyl boronic acid, Pd(OAc)_2_, SPhos, K_3_PO_4_, dry toluene, reflux, 2 h; and ix) 10% aq. NaOH, MeOH, 80 °C, 1–4 h.

The synthesis of the target compounds **3–20** is presented in **Scheme** **1**. Traditionally, the synthesis of the PBTZ derivatives starts with the introduction of the cyclohexyl moiety on the oxygen at the C‐5 position of the commercially available 5‐hydroxy‐2‐methylbenzothiazole **21**. Yet, this initial step presents some challenges due to the low reaction yield, the high production of waste products, and the need for a purification step.^[^ ^20^ ^]^ Indeed, the impossibility of reacting **21** with a cyclohexyl halide, due to the preference for elimination reactions over the desired nucleophilic substitution, has thus far necessitated the use of a Mitsunobu reaction, exploiting the acidity of the C‐5 hydroxyl group of **21**. However, this procedure requires the use of excess PPh_3_ and DIAD as coupling agents, in addition to sonication for 12 h.^[^ ^20^ ^]^ Furthermore, the starting material is not fully consumed and must be removed by basic washes to be recovered before purification of the crude by chromatography. Accordingly, herein, an effort was made to identify new optimized reaction conditions to overcome this shortcoming in the initial step of the synthesis. After various attempts, we discovered that the reaction of 5‐hydroxy‐2‐methylbenzothiazole **21** with cyclohexyl *p*‐toluenesulfonate in dry DMF using Cs_2_CO_3_ as a base at 100 °C for 4 h yielded complete conversion of the starting material, eliminating the necessity for crude purification and yielding derivative **22** in a reaction yield of 95%. This approach effectively addressed the shortcomings observed in the initial step of the synthesis. Subsequent acetylation of the C‐2 methyl group by reaction with diethyl carbonate in the presence of NaH yielded the acetate derivative **23**, as previously reported.^[^ ^20^ ^]^ Then, the recently developed one‐pot three‐component reaction^[^ ^22^ ^]^ was employed to react derivative **23** with DMF‐DMA and phenylacetic anhydride in neat conditions at 110 °C. This was followed by the addition of aqueous 10% NaOH and EtOH, which resulted in the formation of the C‐8 cyclohexyloxy PBTZ acid **24**.^[^ ^22^ ^]^ Subsequent coupling reactions of **24** with various amines in dry DMF at room temperature, in the presence of EDCI·HCl, HOBt, and DIPEA or BOP and Et_3_N, yielded the target compounds **3–8** and **10** and the methyl ester intermediate **25**. A basic hydrolysis under mild conditions of **25** resulted in the formation of the acid compound **9**. The synthesis of the differently C‐2 functionalized PBTZ derivatives **11–20** was not feasible using the three‐component reaction. This was due to the reduced reactivity of the acetic anhydride, which was needed for synthesizing the intermediate **27**, in comparison with the phenylacetic anhydride. Accordingly, the acrylate intermediate **26** was first formed by reacting derivative **23** with DMF‐DMA, as previously reported.^[^ ^22^ ^]^ This intermediate was then reacted with acetic anhydride, resulting in the desired C‐2 unsubstituted PBTZ ester **27**.^[^ ^22^ ^]^ At this point, a selective bromination of PBTZ ester **27** was conducted, yielding the C‐2 bromo derivative **28**. This served as a crucial intermediate in the subsequent Suzuki–Miyaura and Buchwald–Hartwig reactions. Indeed, derivative **28** was reacted with the cyclopropyl boronic acid in the presence of S‐Phos, Pd(OAc)_2_, and K_3_PO_4_ in dry toluene, resulting in the analogue **29**. Conversely, the bromo derivative **28** was reacted with the aniline in dry toluene in the presence of BINAP, Pd(OAc)_2_, and NaO*t*Bu to yield the derivative **30**. Alternatively, derivatives **31**–**33** were obtained by reacting different amines in the presence of Xantphos, Pd_2_(dba)_3_, and K_2_CO_3_. All ester analogues were then subjected to hydrolysis in basic conditions using a mixture of aqueous 10% NaOH solution and MeOH, with the exception of the thiazole derivative **33**, which was reacted in a mixture of 1M LiOH in water and dioxane to preserve thiazole stability, to yield the corresponding acids **34–38**. Subsequently, the amidation reaction was conducted in the presence of EDCI·HCl, HOBt, and DIPEA in dry DMF, affording the target compounds **11–20**.

### 2.2 Design of PBTZ Amide Analogues and Evaluation of Their anti‐DENV‐2 Activity

The anti‐DENV‐2 efficacy and cellular toxicity of the novel analogues of compound **2** in Huh‐7 cells are summarized in Table 1. The initial phase of the design of novel analogues of compound **2** entailed the investigation of the impact of the hydroxyl group present on the C‐4 benzyl substituent. This was achieved by evaluating the antiviral activity of *ortho* and *meta*‐OH derivatives (compounds **3** and **4**, respectively). Both compounds demonstrated comparable anti‐DENV‐2 activity to compound **2**, indicating that the position of the hydroxyl group on the aromatic ring is not a critical factor. However, the *meta* hydroxyl derivative **4** exhibited greater toxicity than the *ortho* and *para* analogues **3** and **2**. Of note, the unsubstituted benzyl analogue was previously reported to lack anti‐DENV‐2 activity, indicating that the hydroxyl substituent is essential for activity.^[^ ^21^ ^]^ Therefore, we sought to elucidate the function of the hydroxyl moiety and designed the synthesis of the methoxy derivative **5**. A complete loss of inhibition of DENV‐2 replication was observed, which highlights the potential essential role of the polarity and H‐bond donor property of the free hydroxyl group. Nevertheless, further data suggested that an H‐bond donor is not a prerequisite at this position. Indeed, nitrile derivative **6** and methylpyridine derivative **7** exhibited sub‐ and low‐micromolar EC_50_ values with no cellular toxicity observed (CC_50_ higher than 100 μM), which resulted in very promising selectivity index (SI > 100). In parallel, the replacement of the hydroxyl group of **2** with more acidic functionalities was investigated with the sulfonamide derivative **8** and the carboxyl derivative **9**. Both compounds exhibited EC_50_ values slightly lower than that of compound **2**, yet appeared to be more toxic, with a CC_50_ of 52.6 and 40.6 μM, respectively. The introduction of an aliphatic sulfonamide resulted in compound **10**, which exhibited not only potent anti‐DENV‐2 activity but also significant cellular cytotoxicity (CC_50_ of 32.9 μM).

At this stage, although some additional information about the SAR was gained, no notable enhancement in the anti‐DENV‐2 activity was observed following the modification of the substituents of the benzyl portion linked to the amide at the C‐4 position of the PBTZ scaffold. Therefore, our attention was directed toward the C‐2 position of the PBTZ core. Previously, modifications to the C‐2 position of PBTZs primarily involved the complete removal of the phenyl ring or its functionalization with various atoms or small groups.^[^ ^20^ ^]^ However, the anti‐DENV results ranged from a loss of antiviral efficacy to minimal improvements, with the most potent compounds never reaching values below the single‐digit micromolar range of anti‐DENV‐2 EC_50_. Therefore, in this study, we decided to investigate more drastic changes regarding the PBTZ C‐2 substituent. The initial investigation focused on the replacement of the C‐2 phenyl ring with a cyclopropyl, exploring its potential as a noncanonical aliphatic isoster. The resulting compounds **11** and **12** demonstrated anti‐DENV‐2 activity that was comparable to that of closely related phenyl analogues **2** and **8**. However, a comparison of the hydroxybenzyl pair revealed that the replacement of the phenyl with the cyclopropyl resulted in an increase in cell toxicity. We proceeded to introduce an amino bridge between the aromatic phenyl substituent at the C‐2 position and the PBTZ tricycle in order to increase the distance between the ring systems. The resulting compounds **13** and **14** yielded disparate outcomes. Indeed, a direct comparison between aniline derivative **13** and its close phenyl analogue **2** revealed a slight improvement in anti‐DENV‐2 activity, although this was accompanied by a slight increase in cytotoxicity. In contrast, a comparison between **14** and its closely related phenyl analogue **8** demonstrated a slight reduction in both anti‐DENV‐2 activity and cytotoxicity.

Additional modifications were attempted at the PBTZ C‐2 position, with the introduction of a pyridin‐3‐amine, a pyrimidin‐2‐amine, and a thiazol‐2‐amine moiety. The resulting compounds **15–20** demonstrated highly promising results, with EC_50_ values ranging from 50 to 280 nM. This finding indicated that C‐2 substituents exerted a significant impact on the anti‐DENV‐2 potency of this series of compounds. In two of the three cases, the presence of sulfonamide functionality resulted in derivatives that exhibited greater toxicity than their respective hydroxy analogues (**18** vs **17** and **20** vs **19**). This trend was not observed in the pyridine‐3‐amine analogues **16** and **15**, which exhibited CC_50_ values exceeding 100 μM against Huh‐7 cells. The thiazolyl analogue **19** exhibited the most promising anti‐DENV‐2 activity, with an EC_50_ value of 50 nM and a CC_50_ value of >100 μM, resulting in a SI value of >2000. It is noteworthy that compound **19** exhibited >30‐fold improvement in anti‐DENV‐2 potency relative to the initial hit **2**, thus establishing itself as the most potent PBTZ analogue identified to date as an anti‐DENV‐2 agent.

### 2.3 Pan‐Serotype anti‐DENV Activity and Investigation on the Mechanism of Action of Derivatives 15 and 19

**Figure 2:**
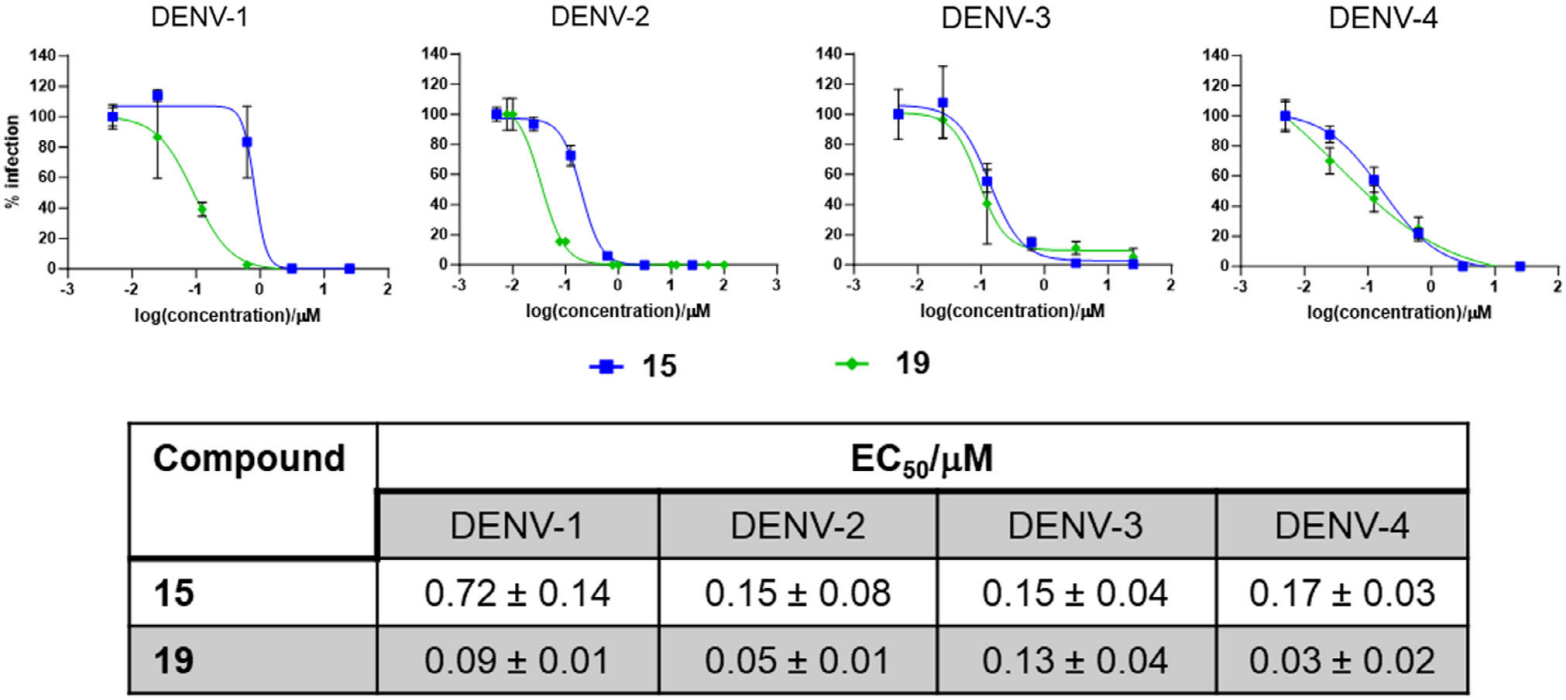
Dose‐dependent antiviral activity of compounds **15** and **19** against the four DENV serotypes (GenBank accessions: EU081230 (DENV‐1); EU081177 (DENV‐2), EU081190 (DENV‐3), and GQ398256 (DENV‐4)). Inset table summarized the effective concentration 50 (EC_50_) of the respective compounds against the different DENV serotype. Data are presented as average with standard deviation from two independent experiments.

At this stage, in view of the high potency observed for some novel PBTZ derivatives, we undertook an investigation into their potential as pan‐serotypes inhibitors of DENV. Consequently, the most promising derivatives, **15** and **19**, were subjected to testing against DENV‐1, −3, and −4 serotypes (**Figure** **2**). It is noteworthy that both compounds exhibited potent anti‐DENV activity against all serotypes, displaying nanomolar to low sub‐micromolar EC_50_ values. Notably, compound **19** was confirmed to be the most potent PBTZ in the series, exhibiting EC_50_ values ranging from 30 to 130 nM.

**Figure 3:**
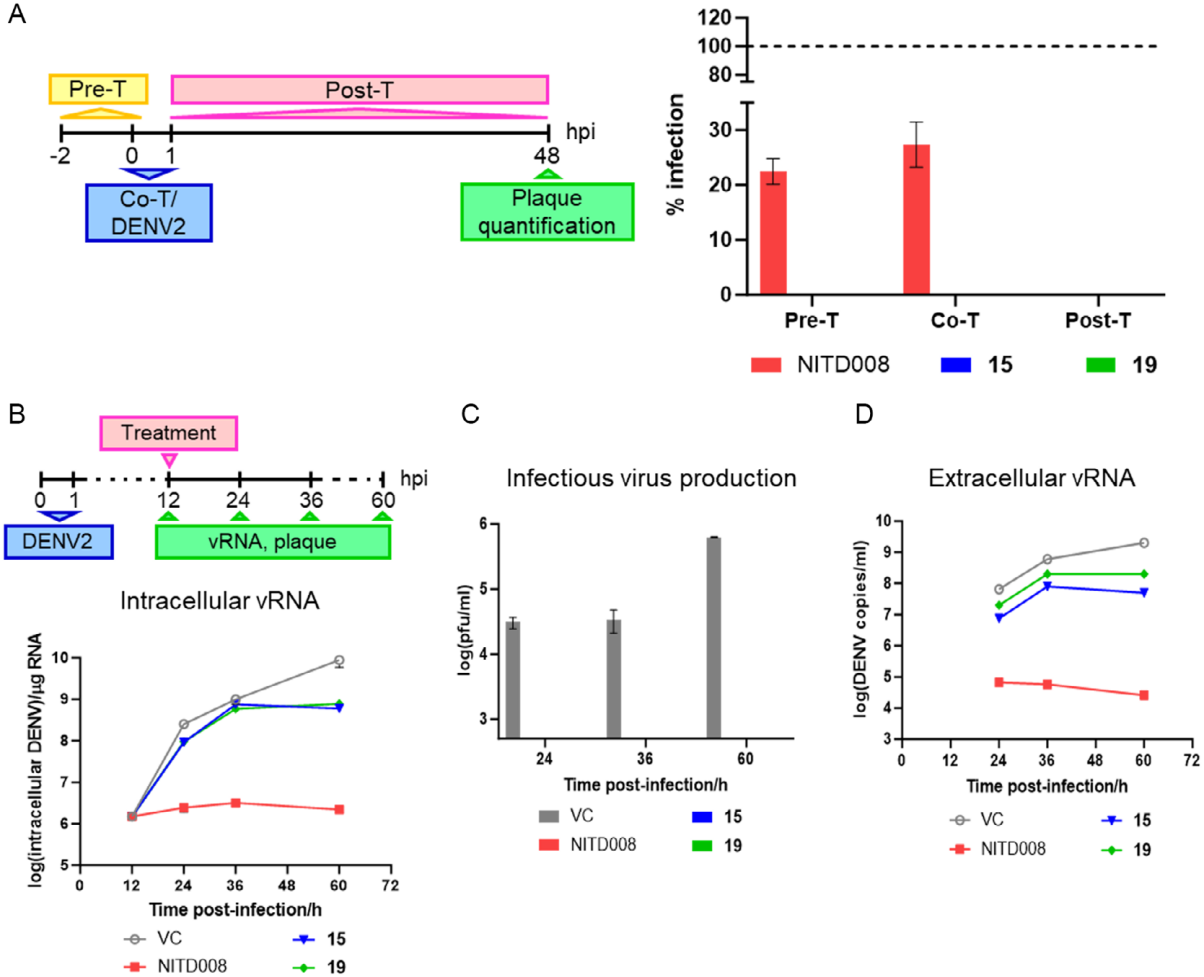
Effect of compounds 15 and 19 on virus replication cycle. A) Schematic showing time‐of‐drug‐addition assay experimental setup (left panel). Huh‐7 cells were either treated with the compounds 2 h prior to DENV‐2 infection (pretreatment; Pre‐T), or infected with DENV‐2 in the presence of the compound (cotreatment; Co‐T), or treated with the compound after the 1 h DENV‐2 infection (posttreatment; Post‐T). 10 μM of the compounds was used for treatment and the endpoint plaque quantification of the culture supernatants was assessed at 48 h postinfection. The bar graph on the right panel shows the percentage of infection of the treated samples to that of the untreated virus control for the various treatment conditions. B–D) Delayed synchronized time‐of‐drug‐addition assay in DENV‐2 infection (see schematic shown in (B). Huh‐7 cells were infected with DENV‐2 at MOI 1 and 10 μM of the compounds were added to the cells at 12 h postinfection. Viral RNA (vRNA) synthesis and infectious virus production were assessed at indicated timepoints postinfection after treatment. Line graph in (B) shows the intracellular vRNA replication profile after treatment with the compounds. C) Infectious virus production determined by standard BHK‐21 plaque assay. D) vRNA detected in the compound‐treated infected culture supernatants. Data are presented as average with standard deviation from two independent experiments.

Subsequently, we investigated whether the inhibitory effect of the two potent amide analogues **15** and **19** against DENV was associated with the inhibition of NS5 polymerase, the target identified for the first series of PBTZ analogues (series A in Figure 1).^[^ ^20^ ^]^ As expected, given the absence of free carboxylic functionality on the C‐4 amide substituent, both compounds did not inhibit both the de novo initiation or elongation activities of NS5 polymerase when tested at 30 μM (Figure S1A, Supporting Information). This finding validated the SAR information around the PBTZ class. To gain insights into the cellular antiviral mechanism, we first conducted a time of drug‐addition study (schematic of **Figure** **3**A) to ascertain which stage of the viral life cycle was susceptible to the compound inhibition. In contrast to the previously described nucleoside inhibitor NITD008,^[^ ^23^ ^]^ the 10 μM concentration of both compounds **15** and **19** demonstrated 90 to >99% virus inhibition across all treatment conditions (pre‐, co‐, and posttreatment), indicating that they potentially act at all stages of the viral life cycle (Figure 3A). However, a delayed synchronized posttreatment assay (12 h postinfection) employing compounds **15** and **19** at 10 μM yielded only a modest reduction in the intracellular viral RNA synthesis (2–2.5 fold) at 24 and 36 h postinfection in comparison with the untreated virus control (Figure 3B). In contrast, the nucleoside inhibitor NITD008 demonstrated consistent suppression of viral RNA replication throughout the course of treatment (Figure 3B). Nevertheless, no infectious virus could be detected by the standard plaque assay in the supernatants of the infected cells treated with PBTZ analogues **15** and **19** (Figure 3C). Given the unconventional mechanism of action, an investigation was conducted to ascertain whether the compounds might affect virion production by examining the amount of viral genome present in the infected culture supernatants. It is noteworthy that 10^7^–10^8^ viral genome copies/mL were detected in the treated culture supernatants, whereas the genome copies/mL in the NITD008‐treated culture supernatant were found to be negligibly low (close to the background threshold of 10^4^ genome copies/mL) (Figure 3D). Furthermore, infection of Vero cells with the resulting Huh‐7 infected supernatants from the delayed synchronized time‐of‐drug‐addition study (Figure 3B) demonstrated that the viruses from the compound‐treated culture supernatants were unable to replicate, in contrast to the untreated virus control culture supernatant (Figure S2, Supporting Information).

**Figure 4:**
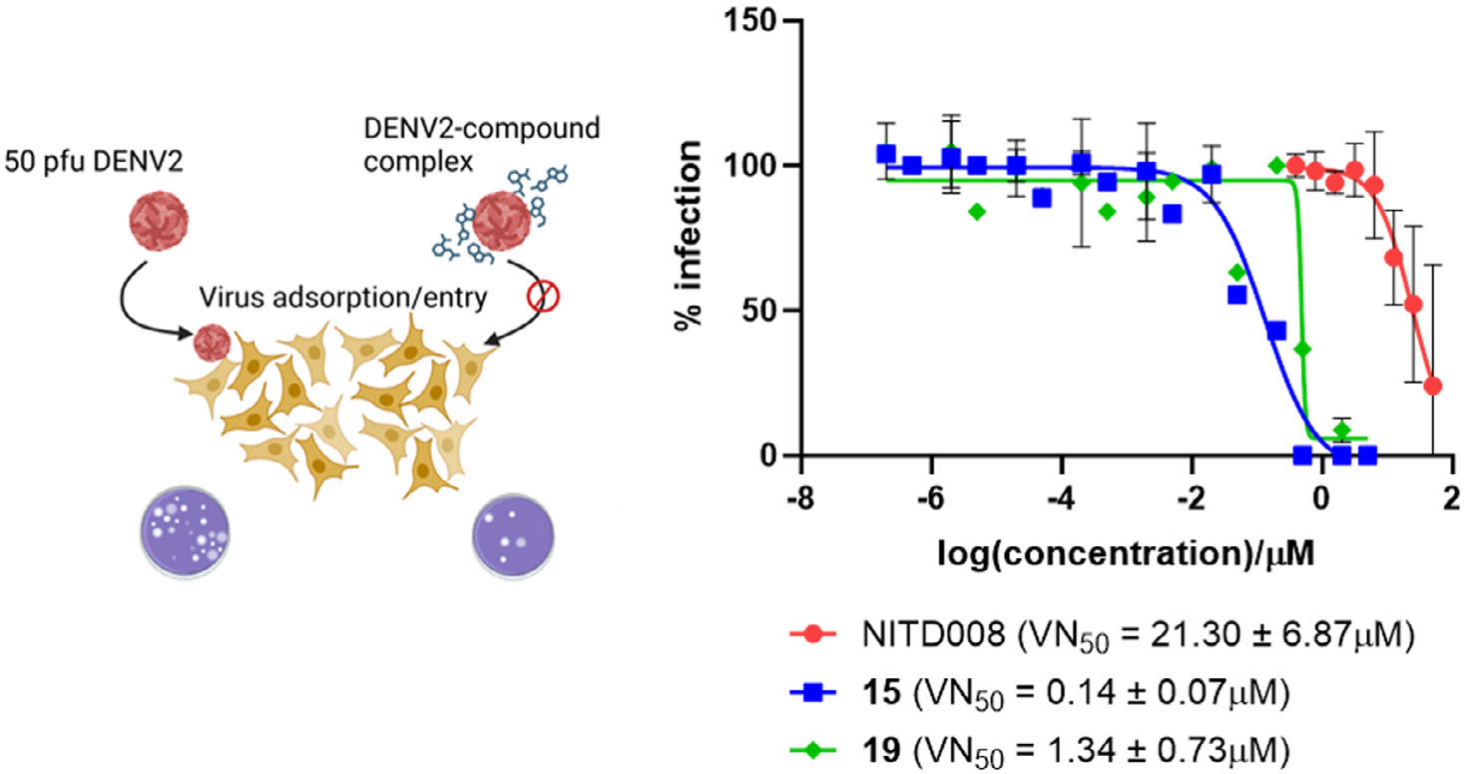
VN assay of PBTZ compounds **15** and **19**. Schematic showing the principle of compound‐mediated VN assay (top panel). Concentration‐dependent VN mediated by PBTZ compounds **15** and **19**, with the efficacy indicated in parentheses (bottom panel). The nucleoside inhibitor NITD008 was included as a negative control. Data are presented as average with standard deviation from two independent experiments.

Collectively, the data demonstrated that compounds **15** and **19** exert minimal influence on viral RNA synthesis. In contrast, an effect during the viral RNA packaging and secretion of the virions into the extracellular milieu can be postulated, which may explain the loss of infectivity of virions in a second infection cycle. In light of these findings, it can be proposed that both compounds possess anti‐DENV activity, potentially due to their ability to bind or modify the viral particle either prior to the establishment of infection or subsequent to the release of new particles during the infection cycle. To validate this hypothesis, a virus neutralization (VN) assay was conducted using PBTZ compounds **15** and **19** and NITD008 as a negative control (**Figure** **4**). The results demonstrated a degree of neutralizing activity for both compounds, with VN_50_ (concentration at which 50% of the virus is neutralized by the compound) values of 0.14 and 1.34 μM, respectively. In contrast, the polymerase nucleoside inhibitor NITD008 demonstrated a VN_50_ value of 21.30 μM. Therefore, although the discrepancy between VN_50_ and EC_50_ values precludes a definitive conclusion, these results indicate that compounds **15** and **19** possess the capacity to bind DENV virions and impede viral adsorption/entry, thereby substantiating their efficacy in pre‐ and cotreatments.

Overall, this behavior is analogous to that previously observed for the PBTZ analogue **1**, which belongs to the first class of PBTZ derivatives with a free carboxylic function on the C‐4 amide substituent.^[^ ^20^ ^]^ Although initially developed as a DENV polymerase inhibitor, with an IC_50_ of 7 μM (unpublished data), compound **1** acted by reducing the infectivity of the released virions from the first cycle of infection, without affecting the amount of viral RNA and particles. However, subsequent detailed analysis revealed that **1** also demonstrated notable virucidal activity (observed in pretreatment experiments) through an as‐yet unclear mechanism involving modification of the virion structure.^[^ ^24^ ^]^ Accordingly, although not affecting polymerase function, it can be postulated that the new PBTZ derivatives **15** and **19** may exhibit a similar mechanism of action to that observed in **1**. However, in comparison with the progenitor **1**, the new compounds **15** and **19** demonstrated a > 50‐fold enhancement in anti‐DENV‐2 activity.

In order to gain further insights into the mechanism of action of this PBTZ series of compounds, we conducted escape mutant experiments in which we serially passaged DENV‐2 in Vero cells using compound **15** at increasing concentrations, as previously described.^[^ ^25^ ^]^ Compound **15** treatment resulted in the induction of 19 single‐nucleotide variants (SNVs) with a variant frequency (*F*_*v*_) exceeding 10%. Of these, 13 unique SNVs (6 nonsynonymous and 7 synonymous changes) were not present in the DMSO‐treated virus (Figure S3, Supporting Information). Although the variant frequency of the SNVs in **15** treated samples was <50% (nonconsensus variants), it is noteworthy that mutation in E protein domain II (leucine to serine substitution in residue 277 which resides in the hinge region between domain I and II) was observed in the **15** treated virus. However, this mutation is not a common mutation observed for previously reported DENV E protein inhibitors,^[^ ^26^ ^]^ which also exhibited a preferential anti‐DENV activity during the early stage of infection.^[^ ^27^, ^28^ ^]^

In addition to the E protein SNV, the **15**‐treated virus also exhibited nonsynonymous SNVs in NS3 and NS4B (inset table of Figure S3, Supporting Information). These SNVs are situated in regions of the NS3 protein that interact with the NS4A‐2K‐4B precursor, and their functional consequences are linked to the formation of replication vesicle packets.^[^ ^29^ ^]^ However, it is expected that inhibitors of NS3 (both protease and helicase enzymatic activities) and of the interaction between NS3 and NS4A‐2K‐4B will affect viral replication in a manner that differs from what was observed for compounds **15** and **19**.^[^ ^29^, ^30^ ^]^ Furthermore, to exclude any potential inhibition of the helicase activity, the NS3 ATPase activity was evaluated for both compounds (Figure S1B, Supporting Information). The results demonstrated that neither compound exhibited any inhibitory effects on helicase activity, confirming that both compounds are not helicase inhibitors.

Consequently, the endeavor to generate escape mutants for the purpose of elucidating the mechanism of action of the PBTZ derivatives was not achieved. Based on the results obtained in this study, it can be posited with a reasonable degree of confidence that all PBTZ analogues share the same peculiar mechanism of action involving a target, which, to date, remains unknown. It is now evident that active PBTZ analogues demonstrate anti‐DENV activity by reducing the infectivity of newly formed viral particles without exerting an effect on either viral RNA synthesis or virion production and polymerase function.

### 2.4 Preliminary In Vitro PK Evaluation of Compound 19

Metabolism is a natural process that occurs in the body to convert drugs, toxins, and nutrients into more easily excretable derivates. However, molecules with a short half‐life are easily metabolized, so they may not remain in the body long enough to have a therapeutic effect. On the other hand, compounds with prolonged half‐lives are not metabolized at all and can accumulate in the body over time, leading to an increase in their concentration and consequently potential toxicity.

The key pharmacokinetic properties of compound **19**, namely, solubility, membrane permeability, plasma stability, and human liver metabolic stability, were determined in order to verify its suitability for in vivo studies.

One of the most common reasons for low drug bioavailability is related to poor water solubility, as a good solubility profile is crucial for drug candidates.^[^ ^31^ ^]^ Therefore, we determined the solubility profile of compound **19** in aqueous solutions and the solubility limit was identified at 42.68 μM with 1% of DMSO at room temperature (**Table** **2**).

**Table 2:** In vitro pharmacokinetic properties of compound **19**.

	PAMPA assayb)		Plasma stabilityc)		Human liver microsomal stability		
Kinetic solubilitya)	*P*_e_ [10^−6^ cm s^−1^]	Memb. retention	1 h	2 h	In vitro t^1^/_2_ [min]d)	CLint_in vitro_ [μL min^−1^ mg^−1^]e)	CLint_in vivo_ [mL min^−1^ Kg^−1^]f)
42.68 μM	4.98 ± 1.43	79.57 ± 4.97%	101.2 ± 1.7%	100.4 ± 2.7%	50.96	27.20	22.37

a) Determined in phosphate‐buffered saline (PBS) (pH 7.4) with 1% DMSO at room temperature.

b) Compound **19** was tested at 20 μM. Values for propranolol and furosemide are consistent with literature data.

c) (%) 100 ‐ [concentration at time points min /concentration at 0 min] ×100.

d) 0.693/*k*, where *k* is the slope of linear regression of the percentage parent compound remaining against time.

e) CLint_in vitro_: In vitro Intrinsic Clearance ln 2/t_1/2_ × [volume of incubation medium (μl)/microsomal protein in incubation (mg)].

f) CLint_in vivo_: In vivo Intrinsic Clearance, CL_int,micr_ × (mg microsome g^−1^ liver) × [liver mass (g)/body mass (kg)].

Membrane permeability was assessed exploiting the well‐validated parallel artificial membrane permeability assay (PAMPA), using a *l*‐α‐phosphatidylcholine as lipid membrane.^[^ ^32^, ^33^ ^]^ The highly permeable drug propranolol and poorly permeable drug furosemide were used as reference compounds to validate the assay. To take into account membrane retention, the apparent permeability coefficient (*P*_app_), calculated using Faller modification of the Sugano equation,^[^ ^34^ ^]^ has been converted to effective permeability coefficient (*P*_e_) using mass balance equations (Table 2).^[^ ^35^ ^]^

As predictable, lipophilic compound **19** (cLog*P* = 5.38) showed a high percentage of membrane retention (*R*_M_ = 79.57 ± 4.97%), probably due to the high aqueous boundary layer at the two sides of the membrane, which is nearly absent in vivo.^[^ ^36^ ^]^ Therefore, the high *P*_e_ value of 4.98 ± 1.43× 10^−6^ cm s^−1^, corrected for the *R*_M_, overestimates its real permeability.

Then, we evaluated the stability of compound **19** against degradation induced by hepatic metabolic enzymes by monitoring the loss of the test compound over time under CYP–UGT‐mediated metabolic pathways. Metabolic stability was assessed by adding UDPGA and NADPH for the activation of both CYPs and UGTs in the presence of alamethicin. The extent of hepatic metabolism was evaluated by monitoring the remaining percentage of parent compound at different time points (Figure S4, Supporting Information) allowing us to determine various pharmacokinetic parameters such as in vitro half‐life, intrinsic clearance in vitro (CLint_in vitro_), and intrinsic clearance in vivo (CLint_in vivo_) (Table 2).

In accordance with the classification system proposed by McNaney et al.^[^ ^37^ ^]^ compounds exhibiting in vitro intrinsic clearance values between 15 and 45 mL min^−1^ kg^−1^ are categorized as intermediate clearance compounds.

Compound **19** exhibited time‐dependent disappearance (Figure S4A,B, Supporting Information), leading to the formation of various metabolites (Figure S4C, Supporting Information). To investigate these products, we analyzed the metabolites generated after incubation with human liver microsomes (HLMs) using high‐resolution MS/MS data and advanced processing algorithms in the Compound Discoverer software. The metabolites were tentatively identified based on their accurate mass, fragmentation patterns, and retention times. LC–MS/MS analysis revealed that the primary metabolites of compound **19** were mono‐oxidized derivatives (Figure S5, Supporting Information). Specifically, the M1 and M2 metabolites exhibited a precursor ion [M − H]^+^ *at m/z* 563 (C_28_H_27_N_4_O_5_S_2_), indicating a 16 Da mass difference from the parent compound, consistent with a single oxidation reaction.

Further examination of the MS/MS spectra suggested that the main metabolic reactions involved the C‐2 hydroxylation of the cyclohexyl ring for the M1 metabolite (RT: 5.47 min, Figure S5A, Supporting Information), while the M2 metabolite (RT: 7.14 min, Figure S5B, Supporting Information) underwent hydroxylation of the benzylic carbon connected to the amide nitrogen atom and adjacent to the aromatic ring. Additionally, we observed a time‐dependent increase in the concentration of a glucuronate derivative (RT: 6.52 min, *m/z* 723), albeit at lower concentrations.

Finally, we evaluated the stability of compound **19** after incubation with human plasma for up to 120 min. Our results indicated that compound **19** remained highly stable throughout the incubation period (Table 2).

## 3 Conclusions

In this study, we employed a ligand‐based strategy to design and synthesize a novel set of PBTZ analogues, starting from the previously reported hit compound **2**, which has been demonstrated to possess inhibitory activity against DENV. Some of the new PBTZ derivatives exhibited high potency against DENV‐2, with EC_50_ values in the nanomolar range and a more than 30‐fold improvement in potency compared to **2**. Of particular note is the observation that the two most potent derivatives, **15** and **19**, retained similar nanomolar efficacy against all four DENV serotypes. The investigation into the mechanism of action revealed that the novel PBTZ derivatives exhibited activity throughout the entire treatment period, irrespective of whether the treatment was initiated before, during, or after the infection. However, analysis of infected cells treated with PBTZ analogues at 12 h postinfection revealed that the amount of intracellular and extracellular viral RNA was comparable to that of the untreated control. Nevertheless, the novel viral particles were unable to establish a new infection, as if the compounds had the ability to “sterilize” them. By integrating these novel data with the existing knowledge regarding the mechanism of action of previously reported PBTZ analogues, we postulate that PBTZ derivatives may function by binding to both preformed and newly formed viral particles, thereby impeding viral replication throughout the entire treatment period. This is also consistent with the data obtained from time‐of‐addition assays. To further illustrate the intriguing nature of this mechanism, it is noteworthy that the first PBTZ analogues also exhibited a comparable “sterilizing” property, despite their classification as polymerase inhibitors. Consequently, it can be posited that all PBTZ derivatives operate through an innovative mechanism of action involving a yet unidentified target.

To obtain further insights into this complex mechanism, we conducted escape mutant experiments in addition to the other experiments described in this work. However, no definitive conclusions could be drawn from these studies, as only a few poorly significant mutations were observed in different viral protein sequences, which could not be linked to the observed mechanism of action of PBTZ analogues.

In conclusion, we have identified the most potent PBTZ analogue to date (compound **19**), with an EC_50_ against DENV‐2 of 50 nM and a SI > 2074. This represents a significant advancement in terms of potency and selectivity compared to previously reported PBTZ analogues, which never reached EC_50_ values under the micromolar range. Although encouraging, the yet not‐ideal PK properties of compound **19**, especially regarding solubility and membrane permeability, hinder its progression to in vivo studies. Accordingly, further medicinal chemistry studies will be required to optimize the PK properties of this potent pan‐serotype anti‐DENV agent.

## 4 Experimental Section

4.1

4.1.1

##### Chemistry Section

Unless otherwise indicated, all starting materials were commercially available. Reagents and solvents were purchased from common suppliers and were used as received. Organic solutions were dried over anhydrous Na_2_SO_4_ and concentrated with an IKA RV 8 V rotary evaporator under reduced pressure. All reactions were routinely checked by TLC on silica gel 60_F254_ (Merck) and visualized by using UV and iodine. Purifications were conducted via flash column chromatography separations employing Merck silica gel 60 (mesh 230–400). Yields refer to purified products and are not optimized. ^1^H NMR and ^13^C NMR spectra were recorded at 400 and 100 MHz, respectively, with a Bruker Advance DRX‐400 instrument. The chemical shifts (δ) are reported in ppm relative to tetramethylsilane and calibrated using residual undeuterated solvent as an internal reference. The coupling constants (*J*) are reported in Hz. The spectra were acquired at 298 K. The data processing was performed with Bruker TopSpin 4.4.0 software, and the spectral data are consistent with the assigned structures. The purity (>95%) was revealed at 254 nm through HPLC analysis using a Jasco LC‐4000 instrument equipped with a UV–visible Diode Array Jasco MD‐4015 and C18 Column (Gemini, Phenomenex), 3 μm, 2 mm × 100 mm. The flow rate was 0.50 mL min^−1^. Method A: acquisition time = 15 min with a gradient consisting of acetonitrile (ACN) and water containing 0.1% formic acid, with a linear increase of ACN from 50 to 100% over 15 min. Method B: acquisition time = 15 min with a gradient consisting of ACN and water containing 0.1% formic acid, with a linear increase of ACN from 20 to 100% over 10 min followed by a 5 min period with 100% ACN. The resulting chromatograms were then analyzed using the ChromNAV 2.0 Chromatography Data System software. The retention time of each peak is expressed in minutes. The detection of HRMS detection was based on electrospray ionization (ESI) in positive or negative polarity, as indicated for each compound, using a QTOF Ion Mobility Agilent 6560 equipped with U(H)PLC 1290 Infinity II.

##### General Procedure A

Under N_2_ atmosphere, to a solution of acidic analogue (1 equiv.) in dry DMSO (3 mL per mmol), BOP (1.5 equiv.), Et_3_N (5 equiv.), and the appropriate amine (2 equiv.) were added, and the reaction was stirred at rt for the specified duration. Subsequently, the mixture was poured into ice/water, and the precipitate was filtered under vacuum to afford the desired compound as solid that was purified as indicated for each compound.

##### General Procedure B

Under N_2_ atmosphere, to a solution of acidic analogue (1 equiv.) in dry DMF (3 mL per mmol), EDCI·HCl (2 equiv.), HOBt·H_2_O (2 equiv.), DIPEA (3.5 equiv.), and the appropriate amine (2 equiv.) were added, and the reaction was stirred at rt for the specified duration. Subsequently, the mixture was poured into ice/water, and the precipitate was filtered under vacuum to afford the desired compound as solid that was purified as indicated for each compound.

##### General Procedure C

Following the creation of a vacuum within a three‐necked flask, under a N_2_ atmosphere, dry toluene (7 mL per mmol) was added to a mixture comprising the ester analogue (1 equiv.), the appropriate amine (3 equiv.), the ligand BINAP or Xantphos (0.03 equiv.), the catalyst Pd(OAc)_2_ or Pd_2_(dba)_3_ (0.03 equiv.), and the base NaO*t*Bu or K_2_CO_3_ (2 equiv.) and the reaction was stirred at reflux for the designed time. Subsequently, the mixture was filtered over Celite, and the filtrate was evaporated to dryness to give an oil that was purified as indicated for each compound.

##### General Procedure D

To a solution of ester analogue (1 equiv.) in MeOH (15 mL per mmol), 10% NaOH in H_2_O (7.5 mL per mmol) was added, and the reaction was stirred at 80 °C for the predefined time. Subsequently, the mixture was poured into ice/water, the pH was adjusted to 5 with 2N HCl, and the precipitate was filtered under vacuum to afford the desired compounds as solids, which were used as such for the next reactions.

##### 5‐(Cyclohexyloxy)‐2‐methyl‐1,3‐benzothiazole (22)

A solution of cyclohexyl *p*‐toluenesulfonate (8.13 g, 31.96 mmol) in dry DMF (8 mL) was dripped under N_2_ atmosphere to a suspension of 2‐methyl‐1,3‐benzothiazol‐5‐ol **21** (2.64 g, 15.98 mmol) and Cs_2_CO_3_ (31.24 g, 95.88 mmol) in dry DMF. The reaction mixture was stirred at 100 °C for 4 h. Subsequently, the mixture was poured into ice and water, and the resulting precipitate was filtered, yielding compound **22** with a 95% yield. ^1^H NMR (400 MHz, CDCl_3_): *δ* = 7.62 (d, *J* = 8.7 Hz, 1H, H‐7), 7.43 (d, *J* = 2.3 Hz, 1H, H‐4), 6.95 (dd, *J* = 2.4 and 8.7 Hz, 1H, H‐6), 4.30–4.23 (m, 1H, OCH), 2.78 (s, 3H, CH_3_), 2.04–1.95 (m, 2H, cyclohexyl‐CH_2_), 1.86–1.78 (m, 2H, cyclohexyl‐CH_2_), 1.62–1.54 (m, 4H, cyclohexyl‐CH_2_×2), 1.42–1.29 (m, 2H, cyclohexyl‐CH_2_).

##### Methyl 4‐[({[8‐(cyclohexyloxy)‐1‐oxo‐2‐phenyl‐1H‐pyrido[2,1‐b][1,3]benzothiazol‐4‐yl]carbonyl}amino)methyl]benzoic acid (25)

Following the general procedure A, starting from compound **24** ^[^ ^22^ ^]^ and using methyl 4‐aminobenzoate (time: 2 h), compound **25** was obtained after filtration and purification by flash chromatography column eluting with cyclohexane/EtOAc 70:30 as a yellow solid in 33% yield (0.14 g). ^1^H NMR (400 MHz, CDCl_3_): *δ* = 9.09 (d, *J* = 2.2 Hz, 1H, Ar‐H), 8.03 (d, *J* = 8.0 Hz, 2H, Ar‐H), 7.77 (s, 1H, Ar‐H), 7.69–7.61 (m, 3H, Ar‐H), 7.46–7.40 (m, 4H, Ar‐H), 7.36 (t, *J* = 7.0 Hz, 1H, Ar‐H), 7.15 (dd, *J* = 2.2 and 8.7 Hz, 1H, Ar‐H), 6.58 (t, *J* = 5.5 Hz, 1H, NH), 4.74 (d, *J* = 5.5 Hz, 2H, CH_2_), 4.49–4.40 (m, 1H, OCH), 3.91 (s, 3H, OCH_3_), 2.03–1.95 (m, 2H, cyclohexyl‐CH_2_), 1.83–1.77 (m, 2H, cyclohexyl‐CH_2_), 1.63–1.58 (m, 4H, cyclohexyl‐CH_2_×2), 1.46–1.38 (m, 2H, cyclohexyl‐CH_2_).

##### 8‐(Cyclohexyloxy)‐N‐(2‐hydroxybenzyl)‐1‐oxo‐2‐phenyl‐1H‐pyrido[2,1‐b][1,3]benzothiazole‐4‐carboxamide (3)

Following the general procedure B, starting from compound **24** ^[^ ^22^ ^]^ and using 4‐methoxybenzylamine (time: 3 h), compound **3** was obtained after filtration and purification by flash chromatography column eluting with CHCl_3_/MeOH 98:2 to 95:5 as a yellow solid in 31% yield (0.12 g). ^1^H NMR (400 MHz, DMSO‐*d*_*6*_): *δ* = 9.57 (s, 1H, OH), 9.09 (t, *J* = 5.6 Hz, 1H, CONH), 8.91 (d, *J* = 2.1 Hz, 1H, Ar‐H), 8.55 (s, 1H, Ar‐H), 7.93 (d, *J* = 8.6 Hz, 1H, Ar‐H), 7.83 (d, *J* = 6.6 Hz, 2H, Ar‐H), 7.47 (t, *J* = 7.7 Hz, 2H, Ar‐H), 7.37 (t, *J* = 7.3 Hz, 1H, Ar‐H), 7.25–7.16 (m, 2H, Ar‐H), 7.08 (t, *J* = 7.1 Hz, 1H, Ar‐H), 6.83 (d, *J* = 8.0 Hz, 1H, Ar‐H), 6.76 (t, *J* = 7.2 Hz, 1H, Ar‐H), 4.50–4.38 (m, 3H, NHCH_2_ and OCH), 2.01–1.93 (m, 2H, cyclohexyloxy‐CH_2_), 1.78–1.69 (m, 2H, cyclohexyloxy‐CH_2_), 1.58–1.25 (m, 6H, cyclohexyloxy‐CH_2_×3). ^13^C NMR (100 MHz, DMSO‐*d*_*6*_): *δ* = 167.65, 164.56, 161.37, 156.36, 151.81, 145.09, 139.02, 136.82, 133.94, 129.91, 129.88, 129.36, 128.44, 127.84, 123.14, 122.44, 121.05, 116.57, 107.62, 105.66, 75.75, 43.01, 31.61, 25.55, 23.50. HPLC: Method A; r_t_ 12.52 min. HRMS (ESI) calculated for C_31_H_28_N_2_O_4_S [M + H]^+^ 525.1843, found 525.1842.

##### 8‐(Cyclohexyloxy)‐N‐(3‐hydroxybenzyl)‐1‐oxo‐2‐phenyl‐1H‐pyrido[2,1‐b][1,3]benzothiazole‐4‐carboxamide (4)

Following the general procedure B, starting from compound **24** ^[^ ^22^ ^]^ and using 4‐methoxybenzylamine (time: 3 h), compound **4** was obtained after filtration and purification by flash chromatography column eluting with CHCl_3_/MeOH 97:3 as a yellow solid in 29% yield (0.11 g). ^1^H NMR (400 MHz, DMSO‐*d*_*6*_): *δ* = 9.34 (s, 1H, OH), 9.18 (t, *J* = 5.3 Hz, 1H, CONH), 8.93–8.88 (m, 1H, Ar‐H), 8.52 (s, 1H, Ar‐H), 7.93 (d, *J* = 8.7 Hz, 1H, Ar‐H), 7.83 (d, *J* = 7.5 Hz, 2H, Ar‐H), 7.47 (t, *J* = 7.6 Hz, 2H, Ar‐H), 7.38 (t, *J* = 7.1 Hz, 1H, Ar‐H), 7.22 (d, *J* = 7.4 Hz, 1H, Ar‐H), 7.13 (t, *J* = 7.6 Hz, 1H, Ar‐H), 6.80–6.76 (m, 2H, Ar‐H), 6.84 (d, *J* = 7.9 Hz, 1H, Ar‐H), 4.51–4.38 (m, 3H, NHCH_2_ and OCH), 2.02–1.92 (m, 2H, cyclohexyloxy‐CH_2_), 1.79–1.70 (m, 2H, cyclohexyloxy‐CH_2_), 1.59–1.25 (m, 6H, cyclohexyloxy‐CH_2_×3). ^13^C NMR (100 MHz, DMSO‐*d*_*6*_): *δ* = 164.36, 161.38, 157.88, 156.35, 151.71, 141.33, 139.04, 136.83, 133.89, 129.76, 129.36, 128.43, 127.81, 123.14, 122.41, 121.13, 118.42, 116.56, 114.58, 114.27, 107.67, 105.82, 75.75, 43.07, 31.62, 25.56, 23.50. HPLC: Method B; r_t_ 11.58 min. HRMS (ESI) calculated for C_31_H_28_N_2_O_4_S [M‐H]^−^ 523.1697, found 523.1686.

##### 8‐(Cyclohexyloxy)‐N‐(4‐methoxybenzyl)‐1‐oxo‐2‐phenyl‐1H‐pyrido[2,1‐b][1,3]benzothiazole‐4‐carboxamide (5)

Following the general procedure B, starting from compound **24** ^[^ ^22^ ^]^ and using 4‐methoxybenzylamine (time: 36 h), compound **5** was obtained after filtration and purification by flash chromatography column eluting with CHCl_3_/MeOH 99.5:0.5 as a yellow solid in 30% yield (0.11 g). ^1^H NMR (400 MHz, DMSO‐*d*_*6*_): *δ* = 9.15 (t, *J* = 5.8 Hz, 1H, CONH), 8.91 (d, *J* = 2.5 Hz, 1H, Ar‐H), 8.48 (s, 1H, Ar‐H), 7.93 (d, *J* = 8.7 Hz, 1H, Ar‐H), 7.81 (d, *J* = 7.3 Hz, 2H, Ar‐H), 7.47 (t, *J* = 7.4 Hz, 2H, Ar‐H), 7.37 (t, *J* = 7.3 Hz, 1H, Ar‐H), 7.29 (d, *J* = 8.7 Hz, 1H, Ar‐H), 7.23 (dd, *J* = 2.4 and 8.7 Hz, 1H, Ar‐H), 6.91 (d, *J* = 8.6 Hz, 2H, Ar‐H), 4.47–4.37 (m, 3H, NHCH_2_ and OCH), 3.73 (s, 3H, OCH_3_), 2.01–1.93 (m, 2H, cyclohexyloxy‐CH_2_), 1.78–1.70 (m, 2H, cyclohexyloxy‐CH_2_), 1.58–1.28 (m, 6H, cyclohexyloxy‐CH_2_×3). ^13^C NMR (100 MHz, DMSO‐*d*_*6*_): *δ* = 164.31, 161.38, 158.76, 156.35, 151.68, 139.03, 136.84, 133.92, 131.85, 129.37, 129.29, 128.42, 127.79, 123.14, 122.40, 121.16, 116.57, 114.20, 107.66, 105.87, 75.76, 55.54, 42.69, 31.61, 25.56, 23.50. HPLC: Method B; r_t_ 13.21 min. HRMS (ESI) calculated for C_32_H_30_N_2_O_4_S [M‐H]^−^ 537.1853, found 537.1844.

##### N‐(4‐cyanobenzyl)‐8‐(cyclohexyloxy)‐1‐oxo‐2‐phenyl‐1H‐pyrido[2,1‐b][1,3]benzothiazole‐4‐carboxamide (6)

Following the general procedure B, starting from compound **24** ^[^ ^22^ ^]^ and using 4‐(aminomethyl)benzonitrile hydrochloride (time: 6 h), compound **6** was obtained after filtration and purification by flash chromatography column eluting with CHCl_3_/MeOH 99.5:0.5 as a yellow solid in 41% yield (0.10 g). ^1^H NMR (400 MHz, DMSO‐*d*_*6*_): *δ* = 9.36 (t, *J* = 5.7 Hz, 1H, CONH), 8.96 (d, *J* = 2.3 Hz, 1H, Ar‐H), 8.55 (s, 1H, Ar‐H), 7.98 (d, *J* = 8.7 Hz, 1H, Ar‐H), 7.87 (d, *J* = 7.1 Hz, 2H, Ar‐H), 7.62 (d, *J* = 8.2 Hz, 2H, Ar‐H), 7.53 (t, *J* = 7.4 Hz, 2H, Ar‐H), 7.44 (t, *J* = 7.3 Hz, 1H, Ar‐H), 7.28 (dd, *J* = 2.4 and 8.7 Hz, 1H, Ar‐H), 4.67 (d, *J* = 5.6 Hz, 2H, NHCH_2_), 4.52–4.45 (m, 1H, OCH), 2.02–1.97 (m, 2H, cyclohexyloxy‐CH_2_), 1.85–1.75 (m, 2H, cyclohexyloxy‐CH_2_), 1.63–1.29 (m, 6H, cyclohexyloxy‐CH_2_×3). ^13^C NMR (100 MHz, DMSO‐*d*_*6*_): *δ* = 164.77, 161.38, 156.39, 151.87, 145.89, 139.03, 136.81, 133.96, 132.80, 129.37, 128.65, 128.45, 127.85, 123.18, 122.46, 121.00, 119.37, 116.59, 110.09, 107.64, 105.55, 75.76, 42.98, 31.61, 25.55, 23.50. HPLC: Method A; r_t_ 12.07 min. HRMS (ESI) calculated for C_32_H_27_N_3_O_3_S [M + H]^+^ 534.1846, found 534.1836.

##### 8‐(Cyclohexyloxy)‐1‐oxo‐2‐phenyl‐N‐(pyridin‐4‐ylmethyl)‐1H‐pyrido[2,1‐b][1,3]benzothiazole‐4‐carboxamide (7)

Following the general procedure B, starting from compound **24** ^[^ ^22^ ^]^ and using 4‐(aminomethyl)pyridine (time: 2 h), compound **7** was obtained after filtration and purification by flash chromatography column eluting with CHCl_3_/MeOH 99:1 to 97:3 as a yellow solid in 32% yield (0.08 g). ^1^H NMR (400 MHz, DMSO‐*d*_*6*_): *δ* = 9.29 (t, *J* = 5.7 Hz, 1H, CONH), 8.89 (d, *J* = 1.7 Hz, 1H, Ar‐H), 8.53–8.49 (m, 2H, Ar‐H), 7.91 (d, *J* = 8.5 Hz, 1H, Ar‐H), 7.83 (d, *J* = 7.4 Hz, 2H, Ar‐H), 7.48 (t, *J* = 7.6 Hz, 2H, Ar‐H), 7.42–7.34 (m, 2H, Ar‐H), 7.21 (dd, *J* = 2.0 and 8.7 Hz, 1H, Ar‐H), 4.56 (d, *J* = 5.4 Hz, 2H, NHCH_2_), 4.44–4.38 (m, 1H, OCH), 2.01–1.92 (m, 2H, cyclohexyloxy‐CH_2_), 1.78–1.69 (m, 2H, cyclohexyloxy‐CH_2_), 1.57–1.28 (m, 6H, cyclohexyloxy‐CH_2_×3). ^13^C NMR (100 MHz, DMSO‐*d*_*6*_): *δ* = 164.73, 161.37, 156.38, 15 186, 150.05, 148.90, 139.01, 136.82, 133.97, 129.36, 128.45, 127.84, 123.16, 122.74, 122.45, 120.99, 116.58, 107.61, 105.66, 75.75, 42.27, 31.61, 25.56, 23.51. HPLC: Method B; r_t_ 7.23 min. HRMS (ESI) calculated for C_30_H_27_N_3_O_3_S [M + H]^+^ 510.1846, found 510.1840.

##### N‐[4‐(aminosulfonyl)benzyl]‐8‐(cyclohexyloxy)‐1‐oxo‐2‐phenyl‐1H‐pyrido[2,1‐b][1,3]benzothiazole‐4‐carboxamide (8)

Following the general procedure A, starting from compound **24** ^[^ ^22^ ^]^ and using 4‐(aminomethyl)benzenesulfonamide acetate (time: 12 h), compound **8** was obtained after filtration and purification by flash chromatography column eluting with CHCl_3_/MeOH 95:5 as a yellow solid in 55% yield (0.11 g). ^1^H NMR (400 MHz, DMSO‐*d*_*6*_): *δ* = 9.29 (t, *J* = 5.8 Hz, 1H, CONH), 8.89 (d, *J* = 2.3 Hz, 1H, Ar‐H), 8.51 (s, 1H, Ar‐H), 7.92 (d, *J* = 8.7 Hz, 1H, Ar‐H), 7.85–7.76 (m, 4H, Ar‐H), 7.94 (d, *J* = 8.3 Hz, 2H, Ar‐H), 7.48 (t, *J* = 7.4 Hz, 2H, Ar‐H), 7.39 (t, *J* = 6.6 Hz, 1H, Ar‐H), 7.32 (bs, 2H, SO_2_NH_2_), 7.22 (dd, *J* = 2.4 and 8.8 Hz, 1H, Ar‐H), 4.59 (d, *J* = 5.6 Hz, 2H, NHCH_2_), 4.45–4.38 (m, 1H, OCH), 2.01–1.93 (m, 2H, cyclohexyloxy‐CH_2_), 1.78–1.69 (m, 2H, cyclohexyloxy‐CH_2_), 1.58–1.28 (m, 6H, cyclohexyloxy‐CH_2_×3). ^13^C NMR (100 MHz, DMSO‐*d*_*6*_): *δ* = 164.58, 161.38, 156.37, 151.80, 144.00, 143.18, 139.01, 136.82, 133.97, 129.37, 128.45, 128.18, 127.83, 126.22, 123.15, 122.44, 121.03, 116.58, 107.62, 105.65, 75.75, 42.92, 32.61, 25.55, 23.50. HPLC: Method B; r_t_ 11.21 min. HRMS (ESI) calculated for C_31_H_29_N_3_O_5_S_2_ [M + H]^+^ 588.1622, found 558.16343.

##### 4‐[({[8‐(Cyclohexyloxy)‐1‐oxo‐2‐phenyl‐1H‐pyrido[2,1‐b][1,3]benzothiazol‐4‐yl]carbonyl}amino)methyl]benzoic acid (9)

A solution of **25** (0.18 mmol, 0.10 g) in a mixture of dioxane (5.00 mL) and 1N LiOH in H_2_O (0.88 mL) was stirred a rt for 16 h. Subsequently, the reaction mixture was concentrated under vacuum in order to remove the dioxane, and then poured into ice/water. The pH was adjusted to 3 with 2N HCl, and the mixture was extracted with EtOAc (x3). The organic phase was subjected to a brine wash, followed by drying with Na_2_SO_4_ and evaporation to dryness, resulting in the formation of a yellow solid. Following crystallization by EtOAc, compound **9** was obtained as a yellow solid in 79% yield. ^1^H NMR (400 MHz, DMSO‐*d*_*6*_): *δ* = 12.92–12.83 (bs, 1H, CO_2_H), 9.28 (t, *J* = 5.7 Hz, 1H, CONH), 8.89 (d, *J* = 2.3 Hz, 1H, Ar‐H), 8.51 (s, 1H, Ar‐H), 7.95–7.89 (m, 3H, Ar‐H), 7.82 (d, *J* = 7.5 Hz, 2H, Ar‐H), 7.51–7.44 (m, 4H, Ar‐H), 7.38 (t, *J* = 7.2 Hz, 1H, Ar‐H), 7.21 (dd, *J* = 2.3 and 8.7 Hz, 1H, Ar‐H), 4.60 (d, *J* = 5.5 Hz, 2H, NHCH_2_), 4.45–4.37 (m, 1H, OCH), 2.02–1.92 (m, 2H, cyclohexyloxy‐CH_2_), 1.79–1.68 (m, 2H, cyclohexyloxy‐CH_2_), 1.58–1.25 (m, 6H, cyclohexyloxy‐CH_2_×3). ^13^C NMR (100 MHz, DMSO‐*d*_*6*_): *δ* = 167.65, 164.56, 161.37, 156.36, 151.81, 145.09, 139.02, 136.82, 133.94, 129.91, 129.88, 129.36, 128.44, 127.84, 123.14, 122.44, 121.05, 116.57, 107.62, 105.67, 75.75, 43.01, 31.61, 25.55, 23.50. HPLC: Method B; r_t_ 11.25 min. HRMS (ESI) calculated for C_32_H_28_N_2_O_5_S [M + H]^+^ 553.1792, found 553.1788.

##### N‐[2‐(aminosulfonyl)ethyl]‐8‐(cyclohexyloxy)‐1‐oxo‐2‐phenyl‐1H‐pyrido[2,1‐b][1,3]benzothiazole‐4‐carboxamide (10)

Following the general procedure B, starting from compound **24** ^[^ ^22^ ^]^ and using 2‐aminoethanesulfonamide hydrochloride (time: 6 h), compound **10** was obtained after filtration and purification by flash chromatography column eluting with CHCl_3_/MeOH 97:3 as a yellow solid in 38% yield (0.10 g). ^1^H NMR (400 MHz, DMSO‐*d*_*6*_): *δ* = 8.92–8.78 (m, 2H, Ar‐H and CONH), 8.38 (s, 1H, Ar‐H), 7.94 (d, *J* = 8.7 Hz, 1H, Ar‐H), 7.80 (d, *J* = 7.4 Hz, 2H, Ar‐H), 7.48 (t, *J* = 7.3 Hz, 2H, Ar‐H), 7.39 (t, *J* = 7.1 Hz, 1H, Ar‐H), 7.22 (dd, *J* = 1.6 and 7.0 Hz, 1H, Ar‐H), 6.97 (bs, 2H, SO_2_NH_2_), 4.47–4.38 (m, 1H, OCH), 3.74–3.66 (m, 2H, NHCH_2_), 3.28 (t, *J* = 6.7 Hz, 2H, CH_2_SO_2_‐), 2.02–1.91 (m, 2H, cyclohexyloxy‐CH_2_), 1.79–1.69 (m, 2H, cyclohexyloxy‐CH_2_), 1.59–1.21 (m, 6H, cyclohexyloxy‐CH_2_×3). ^13^C NMR (100 MHz, DMSO‐*d*_*6*_): *δ* = 164.57, 161.34, 156.39, 151.66, 138.99, 136.80, 133.94, 129.33, 128.48, 127.87, 123.20, 122.41, 120.97, 116.60, 107.64, 105.64, 75.76, 54.09, 35.27, 31.61, 25.56, 23.50. HPLC: acquisition time = 15 min with a gradient consisting of ACN and water containing 0.1% formic acid, with a linear increase of ACN from 30 to 100% over 15 min; r_t_ 11.21 min. HRMS (ESI) calculated for C_26_H_27_N_3_O_5_S_2_ [M + H]^+^ 526.1465, found 526.1460.

##### Ethyl 2‐bromo‐8‐(cyclohexyloxy)‐1‐oxo‐1H‐pyrido[2,1‐b][1,3]benzothiazole‐4‐carboxylate (28)

To a suspension of **27** ^[^ ^22^ ^]^ (0.48 g, 1.28 mmol) in AcOH (6 mL per mmol), Br_2_ (0.08 mL, 1.54 mmol) was added dropwise and then the reaction was stirred at rt for 30 min. Subsequently, 10% Na_2_SO_3_ solution in H_2_O (50 mL) was added to quench the excess of Br_2_ and the precipitate was filtered under vacuum to afford compound **28** as an orange solid in 85% yield (0.49). ^1^H NMR (400 MHz, CDCl_3_): *δ* = 9.01 (d, *J* = 4.2 Hz, 1H, Ar‐H), 8.47 (s, 1H, Ar‐H), 7.64 (d, *J* = 8.7 Hz, 1H, Ar‐H), 7.18 (dd, *J* = 2.4 and 8.7 Hz, 1H, Ar‐H), 4.49–4.40 (m, 3H, OCH_2_CH_3_ and OCH), 2.07–2.01 (m, 2H, cyclohexyl‐CH_2_), 1.82–1.76 (m, 2H, cyclohexyl‐CH_2_), 1.60–1.52 (m, 3H, cyclohexyl‐CH_2_ and cyclohexyl‐CH_2_ × ^1^/_2_), 1.49–1.31 (m, 6H, OCH_2_CH_3_, cyclohexyl‐CH_2_ and cyclohexyl‐CH_2_ × ^1^/_2_).

##### Ethyl 8‐(cyclohexyloxy)‐2‐cyclopropyl‐1‐oxo‐1H‐pyrido[2,1‐b][1,3]benzothiazole‐4‐carboxylate (29)

Following the creation of a vacuum within a three‐necked flask, under a N_2_ atmosphere, dry toluene (4 mL per mmol) was added to a mixture comprising the analogue **28** (0.10 g, 0.22 mmol), cyclopropyl boronic acid (0.09 g, 0.44 mmol), Pd(OAc)_2_ (0.001 g, 0.004 mmol), S‐Phos (0.003 g, 0.007 mmol) and K_3_PO_4_ (0.14 g, 0.67 mmol) and the reaction was stirred at reflux for 2 h. Subsequently, the mixture was filtered over Celite, and the filtrate was evaporated to dryness to afford compound **29** as a yellow solid in 86% yield (0.08 g). ^1^H NMR (400 MHz, CDCl_3_): *δ* = 9.01 (d, *J* = 2.3 Hz, 1H, Ar‐H), 7.59 (s, 1H, Ar‐H), 7.50 (d, *J* = 7.4 Hz, 1H, Ar‐H), 7.03 (dd, *J* = 2.2 and 7.5 Hz, 1H, Ar‐H), 4.41–4.32 (m, 3H, OCH_2_CH_3_ and OCH), 2.13–2.07 (m, 1H, CH), 1.98–19.91 (m, 2H, cyclohexyl‐CH_2_), 1.78–1.71 (m, 2H, cyclohexyl‐CH_2_), 1.57–1.50 (m, 2H, cyclohexyl‐CH_2_), 1.49–1.28 (m, 5H, cyclohexyl‐CH_2_ and OCH_2_CH_3_), 0.99–0.93 (m, 2H, cyclopropyl‐CH_2_), 0.66–0.61 (m, 2H, cyclopropyl‐CH_2_).

##### Ethyl 2‐anilino‐8‐(cyclohexyloxy)‐1‐oxo‐1H‐pyrido[2,1‐b][1,3]benzothiazole‐4‐carboxylate (30)

Following the general procedure C, starting from compound **28** and using aniline (ligand: BINAP; catalyst: Pd(OAc)_2_; base: NaO*t*Bu; time: 7 h), after purification by trituration with Et_2_O, compound **30** was obtained as a yellow solid in 46% yield (0.14 g). ^1^H NMR (400 MHz, CDCl_3_): *δ* = 9.02 (d, *J* = 6.3 Hz, 1H, Ar‐H), 7.93 (s, 1H, Ar‐H), 7.59 (d, *J* = 8.6 Hz, 1H, Ar‐H), 7.31 (t, *J* = 7.3 Hz, 2H, Ar‐H), 7.26 (d, *J* = 7.6 Hz, 2H, Ar‐H), 7.11 (dd, *J* = 2.4 and 8.7 Hz, 1H, Ar‐H), 7.07 (t, *J* = 7.4 Hz, 1H, Ar‐H), 6.82 (s, 1H, NH), 4.46–4.40 (m, 3H, OCH_2_CH_3_ and OCH), 2.07–2.01 (m, 2H, cyclohexyl‐CH_2_), 1.85–1.77 (m, 2H, cyclohexyl‐CH_2_), 1.71–1.58 (m, 4H, cyclohexyl‐CH_2_×2), 1.51–1.45 (m, 5H, cyclohexyl‐CH_2_ and OCH_2_CH_3_).

##### Ethyl 8‐(cyclohexyloxy)‐1‐oxo‐2‐(pyridin‐3‐ylamino)‐1H‐pyrido[2,1‐b][1,3]benzothiazole‐4‐carboxylate (31)

Following the general procedure C, starting from compound **28** and using 3‐aminopyridine (ligand: Xantphos; catalyst: Pd_2_(dba)_3_; base: K_2_CO_3_; time: 1 h), after purification by trituration with Et_2_O, compound **31** was obtained as a yellow solid in 85% yield (1.73 g). ^1^H NMR (400 MHz, DMSO‐*d*_*6*_): *δ* = 8.86–8.83 (m, 1H, Ar‐H), 8.50–8.45 (m, 1H, Ar‐H), 8.28 (s, 1H, NH), 8.11–8.07 (m, 1H, Ar‐H), 7.95–7.91 (m, 1H, Ar‐H), 7.77 (s, 1H, Ar‐H), 7.54 (d, *J* = 6.9 Hz, 1H, Ar‐H), 7.31–7.27 (m, 1H, Ar‐H), 7.23–7.18 (d, *J* = 8.0 Hz, 1H, Ar‐H), 4.45–4.36 (m, 3H, OCH_2_CH_3_ and OCH), 2.02–1.97 (m, 2H, cyclohexyl‐CH_2_), 1.79–1.72 (m, 2H, cyclohexyl‐CH_2_), 1.61–1.54 (m, 4H, cyclohexyl‐CH_2_×2), 1.47–1.35 (m, 5H, cyclohexyl‐CH_2_ and OCH_2_CH_3_).

##### Ethyl 8‐(cyclohexyloxy)‐1‐oxo‐2‐(pyrimidin‐2‐ylamino)‐1H‐pyrido[2,1‐b][1,3]benzothiazole‐4‐carboxylate (32)

Following the general procedure C, starting from compound **28** and using 3‐aminopyridine (ligand: Xantphos; catalyst: Pd_2_(dba)_3_; base: K_2_CO_3_; time: 24 h), after purification by trituration with Et_2_O, compound **32** was obtained as a yellow solid in 84% yield (0.44 g). ^1^H NMR (400 MHz, DMSO‐*d*_*6*_): *δ* = 9.03 (s, 1H, Ar‐H), 8.81 (d, *J* = 1.8 Hz, 1H, Ar‐H), 8.59 (d, *J* = 4.5 Hz, 2H, Ar‐H), 8.29 (s, 1H, NH), 7.96 (d, *J* = 8.7 Hz, 1H, Ar‐H), 7.22 (d, *J* = 8.5 Hz, 1H, Ar‐H), 6.98 (t, *J* = 4.7 Hz, 1H, Ar‐H), 4.45–4.34 (m, 3H, OCH_2_CH_3_ and OCH), 2.04–1.96 (m, 2H, cyclohexyl‐CH_2_), 1.79–1.70 (m, 2H, cyclohexyl‐CH_2_), 1.61–1.48 (m, 4H, cyclohexyl‐CH_2_×2), 1.47–1.38 (m, 5H, cyclohexyl‐CH_2_ and OCH_2_CH_3_).

##### Ethyl 8‐(cyclohexyloxy)‐1‐oxo‐2‐(1,3‐thiazol‐2‐ylamino)‐1H‐pyrido[2,1‐b][1,3]benzothiazole‐4‐carboxylate (33)

Following the general procedure C, starting from compound **28** and using 3‐aminopyridine (ligand: Xantphos; catalyst: Pd_2_(dba)_3_; base: K_2_CO_3_; time: 4 h), after purification by trituration with Et_2_O, compound **33** was obtained as a yellow solid in 90% yield (1.81 g). ^1^H NMR (400 MHz, CDCl_3_): *δ* = 8.98 (s, 1H, Ar‐H), 8.89 (d, *J* = 2.3 Hz, 1H, Ar‐H), 8.21 (bs, 1H, NH), 7.51 (d, *J* = 8.6 Hz, 1H, Ar‐H), 7.29 (d, *J* = 3.8 Hz, 1H, Ar‐H), 7.07 (dd, *J* = 2.4 and 8.7 Hz, 1H, Ar‐H), 6.69 (d, *J* = 3.7 Hz, 1H, Ar‐H), 4.47–4.29 (m, 3H, OCH_2_CH_3_ and OCH), 2.03–1.91 (m, 2H, cyclohexyl‐CH_2_), 1.79–1.70 (m, 2H, cyclohexyl‐CH_2_), 1.65–1.48 (m, 6H, cyclohexyl‐CH_2_×3), 1.41 (t, *J* = 7.1 Hz, 3H, OCH_2_CH_3_).

##### 8‐(Cyclohexyloxy)‐2‐cyclopropyl‐1‐oxo‐1H‐pyrido[2,1‐b][1,3]benzothiazole‐4‐carboxylic acid (34)

Following the general procedure D, starting from compound **39** (time: 1 h), after crystallization by a mixture of Et_2_O/EtOH (3:1), compound **34** was obtained as a yellow solid in 75% yield (0.63 g). ^1^H NMR (400 MHz, DMSO‐*d*_*6*_): *δ* = 13.31 (s, 1H, CO_2_H), 8.89–8.84 (m, 1H, Ar‐H), 7.92 (d, *J* = 8.7 Hz, 1H, Ar‐H), 7.56 (s, 1H, Ar‐H), 7.21 (d, *J* = 8.7 Hz, 1H, Ar‐H), 4.47–4.40 (m, 1H, OCH), 2.11–2.07 (m, 1H, cyclopropyl‐CH), 2.03–1.95 (m, 2H, cyclohexyl‐CH_2_), 1.79–1.71 (m, 2H, cyclohexyl‐CH_2_), 1.61–1.30 (m, 6H, cyclohexyl‐CH_2_×3), 0.97–0.81 (m, 2H, cyclopropyl‐CH_2_), 0.72–0.67 (m, 2H, cyclopropyl‐CH_2_).

##### 2‐Anilino‐8‐(cyclohexyloxy)‐1‐oxo‐1H‐pyrido[2,1‐b][1,3]benzothiazole‐4‐carboxylic acid (35)

Following the general procedure D, starting from compound **30** (time: 4 h), compound **35** was obtained as a yellow solid in 86% yield (0.32 g). ^1^H NMR (400 MHz, DMSO‐*d*_*6*_): *δ* = 13.58 (s, 1H, CO_2_H), 8.83 (d, *J* = 1.9 Hz, 1H, Ar‐H), 7.83 (s, 1H, Ar‐H), 7.79 (d, *J* = 8.7 Hz, 1H, Ar‐H), 7.73 (s, 1H, NH), 7.28 (t, *J* = 8.1 Hz, 2H, Ar‐H), 7.23 (d, *J* = 7.7 Hz, 2H, Ar‐H), 7.15 (dd, *J* = 2.1 and 8.8 Hz, 1H, Ar‐H), 6.81 (t, *J* = 7.1 Hz, 1H, Ar‐H), 4.41–4.35 (m, 1H, OCH), 2.03–1.95 (m, 2H, cyclohexyl‐CH_2_), 1.77–1.73 (m, 2H, cyclohexyl‐CH_2_), 1.59–1.26 (m, 6H, cyclohexyl‐CH_2_×3).

##### 8‐(Cyclohexyloxy)‐1‐oxo‐2‐(pyridin‐3‐ylamino)‐1H‐pyrido[2,1‐b][1,3]benzothiazole‐4‐carboxylic acid (36)

Following the general procedure D, starting from compound **31** (time: 4 h), compound **36** was obtained as a yellow solid in 90% yield (0.29 g). ^1^H NMR (400 MHz, DMSO‐*d*_*6*_): *δ* = 13.81 (s, 1H, CO_2_H), 8.89–8.84 (m, 1H, Ar‐H), 8.53–8.47 (m, 1H, Ar‐H), 8.12–7.92 (m, 3H, Ar‐H and NH), 7.90–7.77 (m, 1H, Ar‐H), 7.53–7.47 (m, 1H, Ar‐H), 7.37–7.27 (m, 1H, Ar‐H), 7.25–7.19 (m, 1H, Ar‐H), 4.42–4.37 (m, 1H, OCH), 2.05–1.98 (m, 2H, cyclohexyl‐CH_2_), 1.80–1.72 (m, 2H, cyclohexyl‐CH_2_), 1.63–1.32 (m, 6H, cyclohexyl‐CH_2_×3).

##### 8‐(Cyclohexyloxy)‐1‐oxo‐2‐(pyrimidin‐2‐ylamino)‐1H‐pyrido[2,1‐b][1,3]benzothiazole‐4‐carboxylic acid (37)

Following the general procedure D, starting from compound **32** (time: 4 h), compound **37** was obtained as a yellow solid in 99% yield (0.46 g). ^1^H NMR (400 MHz, DMSO‐*d*_*6*_): *δ* = 13.52 (s, 1H, CO_2_H), 9.30 (s, 1H, Ar‐H), 8.81 (s, 1H, NH), 8.59 (d, *J* = 4.5 Hz, 2H, Ar‐H), 8.24 (s, 1H, Ar‐H), 7.89 (d, *J* = 8.6 Hz, 1H, Ar‐H), 7.22 (d, *J* = 8.5 Hz, 1H, Ar‐H), 6.97–6.92 (m, 1H, Ar‐H), 4.47–4.41 (m, 1H, OCH), 2.03–1.97 (m, 2H, cyclohexyl‐CH_2_), 1.79–1.72 (m, 2H, cyclohexyl‐CH_2_), 1.60–1.32 (m, 6H, cyclohexyl‐CH_2_×3).

##### 8‐(Cyclohexyloxy)‐1‐oxo‐2‐(1,3‐thiazol‐2‐ylamino)‐1H‐pyrido[2,1‐b][1,3]benzothiazole‐4‐carboxylic acid (38)

To a solution of **33** (0.13 g, 0.27 mmol) in dioxane (15 mL), 1N LiOH in H_2_O (1.35 mL, 1.35 mmol) was added, and the reaction was stirred at 50 °C for 24 h. Subsequently, the mixture was poured into ice/water, the pH was adjusted to 5 with 2N HCl and the precipitate was filtered under vacuum to afford compound **38** in 70% yield (0.08 g). ^1^H NMR (400 MHz, DMSO‐*d*_*6*_): *δ* = 13.45 (bs, 1H, CO_2_H), 10.19 (s, 1H, NH), 9.28 (s, 1H, Ar‐H), 8.88 (s, 1H, Ar‐H), 7.92 (d, *J* = 8.5 Hz, 1H, Ar‐H), 7.38 (s, 1H, Ar‐H), 7.22 (d, *J* = 7.7 Hz, 1H, Ar‐H), 6.98 (s, 1H, Ar‐H), 4.45–4.37 (m, 1H, OCH), 2.07–1.99 (m, 2H, cyclohexyl‐CH_2_), 1.81–1.74 (m, 2H, cyclohexyl‐CH_2_), 1.61–1.25 (m, 6H, cyclohexyl‐CH_2_×3).

##### 8‐(Cyclohexyloxy)‐2‐cyclopropyl‐N‐(4‐hydroxybenzyl)‐1‐oxo‐1H‐pyrido[2,1‐b][1,3]benzothiazole‐4‐carboxamide (11)

Following the general procedure B, starting from compound **34** and using 4‐hydroxybenzylamine (time: 12 h), compound **11** was obtained after filtration and purification by flash chromatography column eluting with CHCl_3_/MeOH 98:2 as a yellow solid in 52% yield (0.20 g). ^1^H NMR (400 MHz, DMSO‐*d*_*6*_): *δ* = 9.30 (s, 1H, OH), 8.96 (t, *J* = 5.9 Hz, 1H, CONH), 8.91 (d, *J* = 2.3 Hz, 1H, Ar‐H), 7.86 (d, *J* = 8.7 Hz, 1H, Ar‐H), 7.82 (s, 1H, Ar‐H), 7.20–7.14 (m, 3H, Ar‐H), 6.73 (d, *J* = 8.5 Hz, 1H, Ar‐H), 4.35–4.37 (m, 3H, NHCH_2_ and OCH), 2.16–2.08 (m, 1H, cyclopropyl‐CH), 2.02–1.94 (m, 2H, cyclohexyloxy‐CH_2_), 1.80–1.71 (m, 2H, cyclohexyloxy‐CH_2_), 1.60–1.28 (m, 6H, cyclohexyloxy‐CH_2_×3), 0.96–0.90 (m, 2H, cyclopropyl‐CH_2_), 0.81–0.76 (m, 2H, cyclopropyl‐CH_2_). ^13^C NMR (100 MHz, DMSO‐*d*_*6*_): *δ* = 164.26, 162.90, 156.80, 156.26, 149.19, 138.59, 130.13, 129.21, 128.41, 126.24, 122.93, 121.10, 116.38, 115.52, 107.38, 105.05, 75.80, 42.66, 31.68, 25.58, 23.58, 10.99, 7.99. HPLC: Method B; r_t_ 9.75 min. HRMS (ESI) calculated for C_28_H_28_N_2_O_4_S [M‐H]^−^ 487.1697, found 487.1703.

##### N‐[4‐(aminosulfonyl)benzyl]‐8‐(cyclohexyloxy)‐2‐cyclopropyl‐1‐oxo‐1H‐pyrido[2,1‐b][1,3]benzothiazole‐4‐carboxamide (12)

Following the general procedure B, starting from compound **34** and using 4‐(aminomethyl)benzenesulfonamide acetate (time: 36 h), compound **12** was obtained after filtration and purification by flash chromatography column eluting with CHCl_3_/MeOH 98:2 as a yellow solid in 13% yield (0.06 g). ^1^H NMR (400 MHz, DMSO‐*d*_*6*_): *δ* = 9.14 (t, *J* = 5.7 Hz, 1H, CONH), 8.90 (d, *J* = 1.7 Hz, 1H, Ar‐H), 7.88–7.76 (m, 4H, Ar‐H), 7.51 (d, *J* = 8.0 Hz, 2H, Ar‐H), 7.31 (bs, 2H, SO_2_NH_2_), 7.18 (dd, *J* = 1.7 and 8.7 Hz, 1H, Ar‐H), 4.57 (d, *J* = 5.1 Hz, 2H, NHCH_2_), 4.46–4.36 (m, 1H, OCH), 2.18–2.09 (m, 1H, cyclopropyl‐CH), 2.03–1.95 (m, 2H, cyclohexyloxy‐CH_2_), 1.79–1.70 (m, 2H, cyclohexyloxy‐CH_2_), 1.59–1.27 (m, 6H, cyclohexyloxy‐CH_2_×3), 0.99–0.91 (m, 2H, cyclopropyl‐CH_2_), 0.82–0.75 (m, 2H, cyclopropyl‐CH_2_). ^13^C NMR (100 MHz, DMSO‐*d*_*6*_): *δ* = 164.58, 162.91, 156.31, 149.40, 144.09, 143.16, 138.58, 128.48, 128.12, 126.38, 126.23, 122.98, 120.96, 116.41, 107.37, 104.79, 75.81, 70.26, 40.84, 31.67, 25.58, 23.56, 11.01, 7.99. HPLC: Method B; r_t_ 9.24 min. HRMS (ESI) calculated for C_28_H_29_N_3_O_5_S_2_ [M‐H]^−^ 550.1476, found 550.1476.

##### 2‐Anilino‐8‐(cyclohexyloxy)‐N‐(4‐hydroxybenzyl)‐1‐oxo‐1H‐pyrido[2,1‐b][1,3]benzothiazole‐4‐carboxamide (13)

Following the general procedure A, starting from compound **35** and using 4‐hydroxybenzylamine (time: 1 h), compound **13** was obtained after filtration and purification by flash chromatography column eluting with CHCl_3_/MeOH 98:2 as a yellow solid in 12% yield (0.03 g). ^1^H NMR (400 MHz, DMSO‐*d*_*6*_): *δ* = 9.30 (s, 1H, OH), 9.06 (t, *J* = 5.6 Hz, 1H, CONH), 8.89 (d, *J* = 1.9 Hz, 1H, Ar‐H), 8.10 (s, 1H, Ar‐H), 7.87–7.78 (m, 2H, Ar‐H and NH), 7.33–7.26 (m, 4H, Ar‐H), 7.19–7.12 (m, 3H, Ar‐H), 6.91–6.85 (m, 1H, Ar‐H), 6.71 (d, *J* = 8.5 Hz, 1H, Ar‐H), 4.41–4.35 (m, 3H, OCH and NHCH_2_), 2.05–1.96 (m, 2H, cyclohexyloxy‐CH_2_), 1.79–1.71 (m, 2H, cyclohexyloxy‐CH_2_), 1.59–1.27 (m, 6H, cyclohexyloxy‐CH_2_×3). ^13^C NMR (100 MHz, DMSO‐*d*_*6*_): *δ* = 164.63, 159.01, 156.80, 156.17, 143.35, 141.13, 138.52, 130.30, 129.68, 129.27, 127.80, 123.10, 121.30, 120.65, 117.71, 116.55, 115.52, 113.54, 107.08, 105.27, 75.89, 42.82, 31.77, 25.64, 23.77. HPLC: acquisition time = 20 min with an isocratic elution consisting of 70% ACN and 30% water containing 0.1% formic acid; r_t_ 13.05 min. HRMS (ESI) calculated for C_31_H_29_N_3_O_4_S [M + H]^+^ 540.1952, found 540.1927.

##### N‐[4‐(aminosulfonyl)benzyl]‐2‐anilino‐8‐(cyclohexyloxy)‐1‐oxo‐1H‐pyrido[2,1‐b][1,3]benzothiazole‐4‐carboxamide (14)

Following the general procedure A, starting from compound **35** and using 4‐(aminomethyl)benzenesulfonamide acetate (time: 6 h), compound **14** was obtained after filtration and purification by flash chromatography column eluting with CHCl_3_/MeOH 95:5 as a yellow solid in 5% yield (0.02 g). ^1^H NMR (400 MHz, DMSO‐*d*_*6*_): *δ* = 9.24–9.19 (m, 1H, CONH), 8.91–8.87 (m, 1H, NH), 8.12–8.08 (m, 1H, Ar‐H), 7.87–7.75 (m, 4H, Ar‐H), 7.53–7.47 (m, 2H, Ar‐H), 7.33–7.25 (m, 6H, Ar‐H and SO_2_NH_2_), 7.17 (d, *J* = 8.7 Hz, 1H, Ar‐H), 6.92–6.86 (m, 1H, Ar‐H), 4.55 (d, *J* = 5.0 Hz, 2H, NHCH_2_), 4.43–4.35 (m, 1H, OCH), 2.05–1.96 (m, 2H, cyclohexyloxy‐CH_2_), 1.81–1.71 (m, 2H, cyclohexyloxy‐CH_2_), 1.60–1.33 (m, 6H, cyclohexyloxy‐CH_2_×3). ^13^C NMR (100 MHz, DMSO‐*d*_*6*_): *δ* = 164.88, 158.92, 156.16, 144.16, 143.20, 143.08, 141.19, 138.43, 129.63, 128.07, 127.86, 126.18, 123.03, 121.11, 120.70, 117.79, 116.53, 113.17, 107.07, 104.92, 75.87, 42.96, 31.69, 25.56, 23.67. HPLC: Method B; r_t_ 10.22 min. HRMS (ESI) calculated for C_31_H_30_N_4_O_5_S_2_ [M‐H]^−^ 601.1585, found 601.15871.

##### 8‐(Cyclohexyloxy)‐N‐(4‐hydroxybenzyl)‐1‐oxo‐2‐(pyridin‐3‐ylamino)‐1H‐pyrido[2,1‐b][1,3]benzothiazole‐4‐carboxamide (15)

Following the general procedure B, starting from compound **36** and using 4‐hydroxybenzylamine (time: 48 h), compound **15** was obtained after filtration and purification by flash chromatography column eluting with CHCl_3_/MeOH 97:3 as a yellow solid in 65% yield (1.20 g). ^1^H NMR (400 MHz, DMSO‐*d*_*6*_): *δ* = 9.27 (s, 1H, OH), 9.04 (t, *J* = 5.6 Hz, 1H, CONH), 8.89 (d, *J* = 2.4 Hz, 1H, Ar‐H), 8.54 (d, *J* = 2.8 Hz, 1H, Ar‐H), 8.12 (s, 1H, Ar‐H), 8.07 (d, *J* = 2.5 Hz, 1H, Ar‐H), 8.04 (s, 1H, NH), 7.85 (d, *J* = 5.8 Hz, 1H, Ar‐H), 7.58 (dd, *J* = 1.2 and 8.2 Hz, 1H, Ar‐H), 7.27 (dd, *J* = 4.6 and 8.2 Hz, 1H, Ar‐H), 7.18 (dd, *J* = 2.4 and 8.7 Hz, 1H, Ar‐H), 7.13 (d, *J* = 8.4 Hz, 2H, Ar‐H), 6.71 (d, *J* = 8.5 Hz, 2H, Ar‐H), 4.41–4.35 (m, 3H, OCH and NHCH_2_), 2.04–1.95 (m, 2H, cyclohexyloxy‐CH_2_), 1.80–1.71 (m, 2H, cyclohexyloxy‐CH_2_), 1.59–1.46 (m, 3H, cyclohexyloxy‐CH_2_ and cyclohexyloxy‐CH_2_ × ½), 1.44–1.34 (m, 2H, cyclohexyloxy‐CH_2_), 1.34–1.25 (m, 1H, cyclohexyloxy‐CH_2_ × ½). ^13^C NMR (100 MHz, DMSO‐*d*_*6*_): *δ* = 164.44, 158.92, 156.76, 156.18, 142.51, 151.23, 140.18, 139.98, 138.46, 130.15, 129.18, 126.85, 124.24, 123.68, 123.06, 121.25, 116.58, 115.48, 115.26, 107.06, 105.05, 75.89, 42.75, 31.70, 25.55, 23.67. HPLC: Method B; r_t_ 6.13 min. HRMS (ESI) calculated for C_30_H_28_N_4_O_4_S [M + H]^+^ 541.1904, found 541.1905.

##### N‐[4‐(aminosulfonyl)benzyl]‐8‐(cyclohexyloxy)‐1‐oxo‐2‐(pyridin‐3‐ylamino)‐1H‐pyrido[2,1‐b][1,3]benzothiazole‐4‐carboxamide (16)

Following the general procedure A, starting from compound **36** and using 4‐(aminomethyl)benzenesulfonamide acetate (time: 2 h), compound **16** was obtained after filtration and purification by flash chromatography column eluting with CHCl_3_/MeOH 97:3 as a yellow solid in 18% yield (0.03 g). ^1^H NMR (400 MHz, DMSO‐*d*_*6*_): *δ* = 9.22 (t, *J* = 5.6 Hz, 1H, CONH), 8.88 (d, *J* = 2.2 Hz, 1H, Ar‐H), 8.57 (s, 1H, NH), 8.14–8.10 (m, 3H, Ar‐H), 7.86–7.78 (m, 3H, Ar‐H), 7.64 (d, *J* = 8.1 Hz, 1H, Ar‐H), 7.51 (d, *J* = 8.2 Hz, 2H, Ar‐H), 7.33–7.30 (m, 3H, Ar‐H and SO_2_NH_2_), 7.19 (dd, *J* = 2.2 and 8.2 Hz, 1H, Ar‐H), 4.56 (d, *J* = 5.4 Hz, 2H, NHCH_2_), 4.41–4.36 (m, 1H, OCH), 1.99–1.96 (m, 2H, cyclohexyloxy‐CH_2_), 1.75–1.72 (m, 2H, cyclohexyloxy‐CH_2_), 1.54–1.23 (m, 6H, cyclohexyloxy‐CH_2_×3). ^13^C NMR (100 MHz, DMSO‐*d*_*6*_): *δ* = 164.72, 158.89, 156.20, 144.09, 143.11, 142.78, 140.96, 140.26, 139.65, 138.41, 128.09, 126.82, 126.19, 124.37, 124.04, 123.07, 121.09, 116.58, 115.24, 107.03, 104.75, 75.88, 42.93, 31.68, 25.55, 23.67. HPLC: Method B; r_t_ 5.74 min. HRMS (ESI) calculated for C_30_H_29_N_5_O_5_S_2_ [M + H]^+^ 604.1674, found 604.1683.

##### 8‐(cyclohexyloxy)‐N‐(4‐hydroxybenzyl)‐1‐oxo‐2‐(pyrimidin‐2‐ylamino)‐1H‐pyrido[2,1‐b][1,3]benzothiazole‐4‐carboxamide (17)

Following the general procedure B, starting from compound **37** and using 4‐hydroxybenzylamine (time: 18 h), compound **17** was obtained after filtration and purification by flash chromatography column eluting with CHCl_3_/MeOH 97:3 as a yellow solid in 62% yield (0.05 g). ^1^H NMR (400 MHz, DMSO‐*d*_*6*_): *δ* = 9.29 (s, 1H, OH), 9.01 (t, *J* = 5.8 Hz, 1H, CONH), 8.90 (s, 1H, Ar‐H), 8.84 (d, *J* = 2.3 Hz, 1H, Ar‐H), 8.53 (d, *J* = 4.8 Hz, 2H, Ar‐H), 8.42 (s, 1H, NH), 7.88 (d, *J* = 8.7 Hz, 1H, Ar‐H), 7.21 (dd, *J* = 2.4 and 8.7 Hz, 1H, Ar‐H), 7.17 (d, *J* = 8.4 Hz, 2H, Ar‐H), 6.91 (t, *J* = 4.8 Hz, 1H, Ar‐H), 6.72 (d, *J* = 8.5 Hz, 2H, Ar‐H), 4.43–4.40 (m, 3H, OCH and NH*CH*_*2*_), 2.00–1.95 (m, 2H, cyclohexyloxy‐CH_2_), 1.76–1.73 (m, 2H, cyclohexyloxy‐CH_2_), 1.57–1.23 (m, 6H, cyclohexyloxy‐CH_2_×3). ^13^C NMR (100 MHz, DMSO‐*d*_*6*_): *δ* = 164.52, 160.50, 158.77, 158.65, 156.75, 156.26, 144.68, 138.43, 130.24, 129.26, 123.65, 123.13, 121.73, 121.10, 116.52, 115.46, 113.45, 107.42, 105.15, 75.84, 42.85, 31.65, 25.56, 23.57. HPLC: Method B; r_t_ 9.87 min. HRMS (ESI) calculated for C_29_H_27_N_5_O_4_S [M‐H]^−^ 540.1711, found 540.1714.

##### N‐[4‐(aminosulfonyl)benzyl]‐8‐(cyclohexyloxy)‐1‐oxo‐2‐(pyrimidin‐2‐ylamino)‐1H‐pyrido[2,1‐b][1,3]benzothiazole‐4‐carboxamide (18)

Following the general procedure B, starting from compound **37** and using 4‐(aminomethyl)benzenesulfonamide acetate (time: 24 h), compound **18** was obtained after filtration and purification by flash chromatography column eluting with CHCl_3_/MeOH 97:3 to 95:5 as a yellow solid in 14% yield (0.04 g). ^1^H NMR (400 MHz, DMSO‐*d*_*6*_): *δ* = 9.20 (t, *J* = 5.7 Hz, 1H, CONH), 8.95 (s, 1H, Ar‐H), 8.84 (d, *J* = 1.4 Hz, 1H, Ar‐H), 8.55 (d, *J* = 4.7 Hz, 1H, NH), 8.44 (s, 1H, Ar‐H), 7.88 (d, *J* = 8.7 Hz, 1H, Ar‐H), 7.80 (d, *J* = 8.1 Hz, 2H, Ar‐H), 7.53 (d, *J* = 8.1 Hz, 2H, Ar‐H), 7.31 (m, 2H, SO_2_NH_2_), 7.22 (dd, *J* = 1.5 and 8.7 Hz, 1H, Ar‐H), 4.59 (d, *J* = 5.4 Hz, 2H, NHCH_2_), 4.44–4.40 (m, 1H, OCH), 1.99–1.96 (m, 2H, cyclohexyloxy‐CH_2_), 1.75–1.72 (m, 2H, cyclohexyloxy‐CH_2_), 1.54–1.23 (m, 6H, cyclohexyloxy‐CH_2_×3). ^13^C NMR (100 MHz, DMSO‐*d*_*6*_): *δ* = 164.88, 160.45, 158.80, 158.61, 156.31, 144.76, 144.14, 143.11, 138.42, 128.13, 126.20, 123.77, 123.17, 121.31, 120.98, 116.55, 113.54, 107.42, 104.88, 75.94, 43.08, 31.65, 25.55, 23.57. HPLC: Method B; r_t_ 9.35 min. HRMS (ESI) calculated for C_29_H_28_N_6_O_5_S_2_ [M‐H]^−^ 603.1490, found 603.1493.

##### 8‐(Cyclohexyloxy)‐N‐(4‐hydroxybenzyl)‐1‐oxo‐2‐(1,3‐thiazol‐2‐ylamino)‐1H‐pyrido[2,1‐b][1,3]benzothiazole‐4‐carboxamide (19)

Following the general procedure B, starting from compound **38** and using 4‐hydroxybenzylamine (time: 6 h), compound **19** was obtained after filtration and purification by flash chromatography column eluting with CHCl_3_/MeOH 98:2 as a yellow solid in 63% yield (0.16 g). ^1^H NMR (400 MHz, DMSO‐*d*_*6*_): *δ* = 10.00 (s, 1H, NH), 9.28 (s, 1H, OH), 9.09 (s, 1H, Ar‐H), 8.93 (t, *J* = 5.8 Hz, 1H, CONH), 8.89 (d, *J* = 2.4 Hz, 1H, Ar‐H), 7.86 (d, *J* = 8.7 Hz, 1H, Ar‐H), 7.32 (d, *J* = 3.7 Hz, 1H, Ar‐H), 7.21–7.16 (m, 3H, Ar‐H), 6.95 (d, *J* = 3.7 Hz, 1H, Ar‐H), 6.71 (d, *J* = 8.5 Hz, 2H, Ar‐H), 4.42–4.39 (m, 3H, OCH and NHCH_2_), 2.00–1.95 (m, 2H, cyclohexyloxy‐CH_2_), 1.77–1.73 (m, 2H, cyclohexyloxy‐CH_2_), 1.59–1.31 (m, 6H, cyclohexyloxy‐CH_2_×3). ^13^C NMR (100 MHz, DMSO‐*d*_*6*_): *δ* = 164.88, 164.21, 157.80, 156.72, 156.18, 142.27, 139.05, 138.50, 130.32, 129.32, 125.85, 123.10, 121.03, 117.18, 116.60, 115.42, 110.39, 106.91, 105.42, 75.91, 42.90, 31.73, 25.56, 23.73. HPLC: Method B; r_t_ 9.75 min. HRMS (ESI) calculated for C_28_H_26_N_4_O_4_S_2_ [M‐H]^−^ 545.1322, found 545.1325.

##### N‐[4‐(aminosulfonyl)benzyl]‐8‐(cyclohexyloxy)‐1‐oxo‐2‐(1,3‐thiazol‐2‐ylamino)‐1H‐pyrido[2,1‐b][1,3]benzothiazole‐4‐carboxamide (20)

Following the general procedure B, starting from compound **38** and using 4‐(aminomethyl)benzenesulfonamide acetate (time: 6 h), compound **20** was obtained after filtration and purification by flash chromatography column eluting with CHCl_3_/MeOH 97:3 to 95:5 as a yellow solid in 62% yield (0.–3 g). ^1^H NMR (400 MHz, DMSO‐*d*_*6*_): *δ* = 10.06 (s, 1H, NH), 9.16 (s, 1H, Ar‐H), 8.91 (t, *J* = 5.9 Hz, 1H, CONH), 7.90 (d, *J* = 6.5 Hz, 1H, Ar‐H), 7.85 (d, *J* = 6.7 Hz, 2H, Ar‐H), 7.53 (d, *J* = 7.3 Hz, 1H, Ar‐H), 7.35–7.30 (m, 3H, Ar‐H and SO_2_NH_2_), 7.19 (dd, *J* = 2.3 and 8.8 Hz, 1H, Ar‐H), 6.97 (d, *J* = 2.5 Hz, 1H, Ar‐H), 4.57 (d, *J* = 5.6 Hz, 2H, NHCH_2_), 4.42–4.35 (m, 1H, OCH), 2.01–1.99 (m, 2H, cyclohexyloxy‐CH_2_), 1.77–1.73 (m, 2H, cyclohexyloxy‐CH_2_), 1.59–1.37 (m, 6H, cyclohexyloxy‐CH_2_×3). ^13^C NMR (100 MHz, DMSO‐*d*_*6*_): *δ* = 165.23, 164.15, 157.79, 156.22, 144.25, 143.07, 142.40, 139.05, 138.50, 128.16, 126.15, 125.97, 123.14, 120.90, 116.82, 116.63, 110.49, 106.90, 105.14, 75.92, 43.14, 31.73, 25.56, 23.73. HPLC: Method B; r_t_ 9.32 min. HRMS (ESI) calculated for C_28_H_27_N_5_O_5_S_3_ [M + H]^+^ 610.1247, found 610.1244.

##### Biological Section: Cell Lines and Viruses

Huh‐7 cells (human hepatocarcinoma cells, ATCC) and Vero cells (Green African monkey kidney fibroblast cells, ATCC) were cultured in DMEM medium (Gibco) with 10% (v/v) fetal bovine serum (FBS), 4.5 g L^−1^ glucose and 1% (v/v) penicillin–streptomycin (P/S) at 37 °C in 5% CO_2_. BHK‐21 (baby hamster kidney fibroblast cells, ATCC) were maintained in RPMI 1640 medium (Gibco) supplemented with 10% FBS and 1% P/S at 37 °C in 5% CO_2_. C6/36, an *A. albopictus* cell line (ATCC), was maintained in RPMI 1640 medium containing 10% FBS, 25 mM HEPES, and 1% P/S at 28 °C in the absence of CO_2_. All cell lines used in this study had been tested negative for mycoplasma.

DENV‐1‐4 (GenBank accessions: EU081230; EU081177, EU081190, and GQ398256, respectively) used in this study were obtained from the Early Dengue infection and outcome (EDEN) study.^[^ ^38^ ^]^ The viruses were expanded in C6/36 cells, titered in BHK‐21 cells, and stored at −80 °C until further use for infection. Institutional approval to work with DENV has been granted by Duke‐NUS Medical School.

##### DENV‐2 In Vitro *Polymerase Assays*

The *de novo* initiation and elongation assays were performed exactly as described previously.^[^ ^21^ ^]^ 30 μM of the compounds were added to the reaction mix to test the inhibitory effect of the compounds on polymerase activity. The effect of the compounds on the DENV‐2 polymerase activity is presented as percentage of fluorescence of the enzyme with the compound to that of the enzyme without the compound.

##### DENV‐2 In Vitro *NS3 ATPase Assay*

The NS3 ATPase assay was conducted as previously described with slight modifications.^[^ ^39^ ^]^ Briefly, a 10 × ATPase buffer was prepared as follows: 500 mM Tris‐HCl pH 7.5, 20 mM MgCl_2_, 15 mM DTT, 2.5 μg/mL BSA, and 0.5% Tween‐20. The protein, RNA, and compounds were prepared and diluted in a 1 × ATPase buffer. Purified DENV2 CF_18_NS3 at a concentration of 5 nM was preincubated with 10 μg mL^−1^ of Poly U RNA and 30 μM of compounds in a 96‐well half area transparent plate for 15 min at 37 °C. The reaction was initiated by the addition of 100 μM ATP and incubated at 37 °C for 30 min. The reaction was stopped by adding 10 μL of malachite green and incubating at room temperature for 5–10 min. Absorbance was measured at 635 nm.

##### Cell Viability Assay

The cytotoxicity of the compounds in Huh‐7 cells was evaluated using CellTiter Glo Luminescent Assay (Promega) kit according to manufacturer's instructions. Briefly, Huh‐7 cells were treated with the compounds at indicated concentrations for 48 h. Cell viability is presented as percentage of luminescence derived from treated sample to that of the untreated cell control.

##### Virus Inhibition Assay

Huh‐7 cells were infected with DENV at MOI (Multiplicity of Infection) 0.3 for 1 h, followed by treatment with the compounds either at a single concentration of 10 μM or a range of indicated concentrations (dose‐dependent response) for 48 h. Supernatants were collected, clarified, and subjected to plaque quantification by standard BHK‐21 plaque assay.^[^ ^40^ ^]^ The virus inhibition level at single concentration 10 μM is presented as percentage of virus reduction of the treated samples to that of the untreated virus control (Table 1) while the antiviral efficacy of the compounds (EC_50_: concentration at which the virus infection is reduced by 50%) was determined by the sigmoidal dose response curve of the percentage of infection (virus titer of treated sample to that of the mocked treated virus control) against concentration in GraphPad Prism v.10 (GraphPad Software Inc., San Diego, CA).

##### Time of Drug‐Addition Assay

Time of drug‐addition assay was performed as shown in the schematic in Figure 3A ^[^ ^41^ ^]^ to examine the infection stage at which the compounds exert its antiviral activity. Briefly Huh‐7 cells are either pre‐exposed to 10 μM of the indicated compounds for 2 h prior to DENV‐2 infection (pretreatment; Pre‐T) or treated with the compounds during (cotreatment; Co‐T) or after the 1 h infection (posttreatment; Post‐T). DENV‐2 infection was performed at MOI 1 and posttreatment was performed for 48 h. Supernatants were collected, clarified, and subjected to plaque quantification. The percentage infection is presented as the virus titer of treated samples to that of the untreated virus control.

To assess the inhibitory effect of the compounds on viral RNA replication, a delayed time of drug‐addition study was performed as shown in the schematic in Figure 3B. Huh‐7 cells were infected with DENV‐2 at MOI 1 and at 12 h postinfection, the infected cells were treated with 10 μM of the indicated compounds. At 24, 36, and 60 h postinfection, supernatants were harvested for plaque quantification while the cells were washed once with PBS prior to lysing with TRizol (Invitrogen) for total intracellular viral RNA quantification by real‐time quantitative PCR (real‐time qPCR).

##### RNA Extraction and Real‐Time Quantitative PCR (Real‐Time qPCR)

Total RNA was isolated from the cell lysates using TRizol extraction method. 500 ng of the extracted RNA was used for cDNA synthesis using Improm II Reverse Transcription System (Promega) according to manufacturer's instructions. 40 ng of the cDNA was then used for real‐time qPCR by SYBR green supermix for detection viral RNA with DENV‐2 specific primers and housekeeping actin primers as described elsewhere.^[^ ^42^ ^]^ DENV‐2 EDEN 3295 (GenBank accession: EU081177) plasmid was used to generate the standard curve for quantification of absolute viral genome copies and the intracellular viral RNA expression is reported as actin‐normalized absolute genome copies per microgram total RNA.

##### Virus Neutralization Assay

50 pfu of DENV‐2 was coincubated with increasing concentrations of the compounds on ice for 1 h to form the virus‐compound complex. The virus‐compound mixture was subsequently used to infect BHK‐21 cells for 1 h at 37 °C followed by overlaying with 0.8% carboxylmethylcellulose. The infected cells were further incubated at 37 °C for 5 days, fixed with 4% formaldehyde and stained with 1% crystal violet for visualization of plaques. The VN is presented as a percentage of the viral plaque numbers of treated samples to that of the mocked treated virus control against compound concentration to determine the efficacy using the sigmoidal dose‐response regression in GraphPad Prism v.10 (GraphPad Software Inc., San Diego, CA).

##### In vitro *Resistance Selection (IVRS)*

In vitro resistance selection (IVRS) against the selected indicated compound **15** was performed as described elsewhere^[^ ^25^ ^]^ with slight modifications. Briefly Vero cells were infected with DENV‐2 at MOI 1 followed by treatment with increasing concentrations of **15** from 0.1 to 6 μM. The resultant passaged virus inoculums from each IVRS passage were collected after 3–4 days postinfection when cytopathic effect was observed and subsequently used for the next round of infection in Vero cells followed by treatment with the same aforementioned concentration range. This infection and treatment cycle was repeated for 12 passages.

##### Virus Next‐Generation Sequencing

Viral RNA from the Passage 12 IVRS viral supernatants was extracted using QIAamp Viral RNA Mini kit (Qiagen) following manufacturer's instructions. The extracted viral RNA was used to construct Next‐Generation Sequencing (NGS) libraries using the NEBNext ultra II directional RNA Library Prep kit for Illumina (New England Biolabs) following manufacturer's instructions. The quality of the libraries was assessed by DNA1000 Bioanalyzer chip (Agilent Technologies) prior to paired‐end sequencing on the Illumina MiSeq (2 × 250 bp) sequencer in Duke‐NUS Genome Biology Facility. A typical MiSeq coverage for each nucleotide position ranges from 8000 to 10 000 reads/nucleotide and samples with an overall read depth less than 4000 were excluded from variant call analysis. Sample replicates were pooled and aligned to DENV‐2 EDEN 3295 (GenBank accession: EU081177) for variant calling rather than averaging the individual replicates as previously described.^[^ ^43^ ^]^ Variant allele frequency was analyzed by lofreq software as previously described.^[^ ^44^ ^]^

##### Pharmacokinetic Properties Determination: Determination of Kinetic Solubility

A calibration curve was constructed for the test compound. From a 20 mM stock solution of the compound in DMSO, three dilutions were performed to obtain solutions of the compound at 200, 50, and 10 μM, with a final ratio of DMSO to PBS solution (pH 7.4) of 1:1. Absorbance was measured for each concentration in duplicate in a 96‐well plate (200 μL per well) (Corning 96‐well UV‐Transparent) using the microplate reader Infinite M200 Pro TECAN, and the wavelength of maximum absorption (*λ*_max_) was determined. The calibration curve was constructed by plotting the absorbance at *λ*_max_ against the concentration, and the linear regression was determined using the linear regression calculator provided by GraphPad Prism 10.0 software (GraphPad Software Inc., San Diego, CA). A solution of buffer and DMSO in a 1:1 ratio was used as blank.

10 μL of the test compound was added to 990 μL of buffer (PBS_7.4_) and the mixture was mixed at 1000 rpm for 24 h at 25 °C. Subsequently, the sample was subjected to centrifugation at 10 000 rpm at 25 °C for 10 min, after which 100 μL of the supernatant were collected and diluted with 100 μL of DMSO (final ratio DMSO/buffer PBS_7.4_ 1:1). The absorbance at λ_max_ was then measured in a 96‐well plate (200 μL per well). The measured values were corrected for the corresponding dilution factor. Compound was tested in triplicate.

##### In Vitro *Membrane Permeability and Metabolism*

All additives and mobile phases were LCMS grade and purchased from Merck (Milan, Italy).

##### Instrumentation and LC–MS/MS Conditions

LC**–**MS/MS analysis was performed on a Vanquis UHPLC system connected online to a Orbitrap Exploris 120 mass spectrometer (Thermo Fisher Scientific, Bremen, Germany) equipped with a heated electrospray ionization probe (HESI II).

The chromatographic separation was performed on a Kinetex 2.6 μm Evo C18 100 Å column (100 × 2.1 mm, Phenomenex, Bologna, Italy) employing as mobile phases: A) H_2_O and B) ACN, both acidified with 0.1% v/v formic acid with the following gradient: 0.01–10.00 min, 5–95% B; isocratic to 95% B for 1 min; 11.01–11.50 min, 95–5% B; followed by 1.5 min for re‐equilibration of the analytical column. The flow rate of mobile phases and column oven were set at 0.4 mL min^−1^ and 40 °C, respectively.

The ESI was operated in positive mode. Full MS parameters: Orbitrap resolution: 60 000; scan range (*m/z*): 100‐1500; RF lens (%): 70; normalized AGC target (%): 200; maximum injection time (ms): 200. Data‐dependent MS/MS: Orbitrap resolution 15 000; isolation window (*m/z*): 2; collision energy type: normalized; HCD collision energy (%): 30. Ion source parameters: Sheath gas pressure: 60 arbitrary units; auxiliary gas flow: 15 arbitrary units; sweep gas: 2 arbitrary units; ion transfer tube temp. (°C): 300; vaporizer temp. (°C): 300; spray voltage, +3.4 kV, −3.0 kV.

For the calibration curve, the primary stock solutions were prepared in DMSO. Intermediate and working standard solutions were prepared by serial dilution of the stock solutions in methanol to obtain necessary concentrations. Tolbutamide was used as the internal standard (IS, 1 μM). The analytical method was validated for a concentration range of 0.125–20 μM, demonstrating linearity with a correlation coefficient (*R* ^2^) of ≥0.996. The limits of detection (LOD) and quantification (LOQ) were determined using the standard deviation (SD) of the response and the slope of the calibration curve, applying the factors 3.3 and 10, respectively. Specifically, LOD was calculated as 0.029 μM and LOQ as 0.089 μM.

##### Parallel Artificial Membrane Permeability Assay

Donor solutions (100 μM) for reference compounds (furosemide, propranolol) and donor solution (20 μM) for compound **19** were prepared from DMSO stock solutions diluted with 1X PBS, pH 7.4, 20% DMSO final concentration. The artificial lipid membrane solutions for PAMPA were prepared by dissolving phosphatidylcholine in dodecane at 1% (w/v). The 96‐well microfilter plates (MultiScreen‐IP, 0.45 μm, catalogue no. MAIPN4550) and the 96‐well microtiter plate (MultiScreen‐acceptor, catalogue no. MSSACCEPTOR) were used as donor and acceptor compartments. Filters of donor plates were coated with 5 μL of freshly prepared artificial membrane solutions. After applying the lipids, acceptor plate wells were charged with 300 μL of 1X PBS, pH 7.4, 20% DMSO solution. Donor filter plates, charged with 150 μL of donor solutions, were placed onto the acceptor plates. The resulting sandwich was incubated at room temperature for 8 h under stirring on Heidolph TITRAMAX 100 (600 rpm). Then, the sandwich plates were separated, and concentrations were determined by LC–MS/MS analysis.

All compounds were tested in triplicate, and apparent (*P*_app_, Log*P*_app_) and effective (*P*_e_, Log*P*_e_) permeability values, as well as the *R*_M_ values, were reported as mean ± standard deviation. The apparent permeability value, *P*_app_, was calculated based on the Faller modification of the Sugano equation^[^ ^34^ ^]^ Equation (1):

(1) Papp=−[ (VDVR)(VD+VR)*At] * ln(1−r)

*V*_R_ is the volume of the acceptor compartment (0.300 cm^3^), *V*_D_ is the donor volume (0.150 cm^3^), *A* is the accessible filter area (0.266 cm^2^), *t* is the incubation time in seconds, and *r* is the ratio of acceptor and equilibrium solution concentrations.

The effective permeability value, *P*_e_, was calculated by Equation (2):^[^ ^35^ ^]^

(2) $$P_{e}-\frac{2.303\;V_{D}}{At}\;\left(\frac{V_{A}}{V_{D}+V_{A}}\right)\text{ Log }\left[1-\left(\frac{V_{A}+V_{D}}{V_{D}\left(1-R_{M}\right)}\right)\;\frac{C_{\text{A }\left(t\right)}}{C_{D\left(0\right)}}\right]$$

*C*_D_ *(t)* and C_A_(t) are the compound concentrations in the donor and acceptor compartments, respectively, at time *t*, and *C*_D_ *(0)* is the compound concentration in the donor compartment at time 0. *R*_M_ is the membrane retention factor, calculated using Equation (3):

(3) $$R_{M}=1-\frac{C_{\text{D }\left(t\right)}}{C_{D\left(0\right)}}-\frac{V_{A}}{V_{D}}\frac{C_{\text{A }\left(t\right)}}{C_{\text{D }\left(0\right)}}$$

##### Microsomal Stability

The liver microsomal stability assay of compound **19** was conducted as previously described.^[^ ^45^ ^]^ Briefly, HLMs (Thermo Fisher Scientific, Bremen, Germany) were preincubated with alamethicin. The reaction was initiated by adding a mixture containing NADPH and UDPGA co‐factors. The incubation was carried out 37 °C for 15, 30, 45, and 60 min in a Thermomixer comfort (Eppendorf, Hamburg, Germany). The reaction was stopped by the addition of ice‐cold methanol containing IS. Finally, the samples were centrifuged at 14 000 rpm at 4 °C for 7 min (Eppendorf microcentrifuge 5424, Hamburg, Germany) and the supernatants were injected into the LC–MS. The control at 0 min was established by adding the organic solvent immediately after incubation with microsomes. Testosterone was employed as positive control, while the negative control involved incubation without cofactors for up to 60 min. The extent of metabolism is expressed as the percentage of turnover of the parent compound. All experiments were performed in triplicate.

The results were expressed in terms of in vitro microsome half‐life (*t*_1/2_), in vitro intrinsic clearance (CLint_in vitro_), and in vivo intrinsic clearance (CLint_in vivo_).

The in vitro half‐life (*t*_1/2_) was calculated using the expression *t*_1/2_ = 0.693/*b*, where *b* is the slope found in the linear fit of the natural logarithm of the fraction remaining of the parent compound versus incubation time.

The in vitro intrinsic clearance was calculated as CLint_in vitro_ = (1000) × (0.693/*t*_1/2_)/0.5.

In vitro clearance was scaled to the intrinsic in vivo clearance (CLint_in vivo_) using human physiology‐based scaling factor (PBSF): CLint_in vivo_ = CLint_in vitro_ × PBSF (microsome protein/gram liver: 32 × gram liver/kg b.w.: 25.7).^[^ ^46^ ^]^

##### Plasma Stability Assay

Human plasma was equilibrated to 37 °C, and biotransformation was initiated by adding compound **19**. At each specified time point (0, 60, and 120 min), the test compound was extracted with ice‐cold methanol to halt degradation, with the IS added during the quenching phase. The concentration of the test compound was then quantified using LC–MS. The percentage of the remaining test compound (relative to the 0 min time point) at each time point was reported. All experiments were performed in duplicate. Procaine (low stability) and procainamide (high stability) were used as controls.

## Conflict of Interest

The authors declare no conflict of interest.

## Supporting information

Supplementary Material

## Data Availability

The data that support the findings of this study are available from the corresponding author upon reasonable request.
