# Supplementary material for: New Pyridobenothiazolone Derivatives Display Nanomolar Pan‐Serotype Anti‐Dengue Virus Activity
Source: ChemMedChem. 2025 Apr 21;20(13):e202500163. doi: 10.1002/cmdc.202500163 (PMC12221118; doi:10.1002/cmdc.202500163)
Supplement: Supplementary file 1 — Supplementary Material [file CMDC-20-e202500163-s001.pdf]

Supporting Information

New pyridobenothiazolone derivatives display nanomolar pan-serotype anti-DENV activity

Tommaso Felicetti,[a,1,]* Chin Piaw Gwee,[b,1] Kitti Wing Ki Chan,[b,1,]* Giacomo Pepe,[c] Ciro Milite,[c] Pietro Campiglia,[c] Satoru Watanabe,[b,] Muhammad Danial Bin Mohd Mazlan,[b] Stefano Sabatini,[a] Serena Massari,[a] Oriana Tabarrini,[a] Gianluca Sbardella,[c] Subhash G. Vasudevan,[b,d] Giuseppe Manfroni.[a]

[a]Dipartimento di Scienze Farmaceutiche, Università degli Studi di Perugia, Via Del Liceo, 1-06123, Perugia, Italy

[b]Program in Emerging Infectious Diseases, Duke-NUS Medical School, 169857, Singapore

[c] Dipartimento di Farmacia, Università degli Studi di Salerno, Via Giovanni Paolo II, 132, 84084 Fisciano, Italy

[d]Institute for Biomedicine and Glycomics, Griffith University, Queensland, 4222, Australia

[1]Co-first authors

*Corresponding authors. Email addresses: T.F.: tommaso.felicetti@unipg.it. K.W.K.C.: kitti.chan@duke-nus.edu.sg.

Table of Contents

Figure S1. *In vitro* biochemical inhibitory activities of PBTZ compounds **15** and **19** S2

Figure S2. Viral RNA replication profile of the resultant compound-treated DENV-2 on Vero cells S2

Figure S3. Next-generation-sequencing (NGS) of the serially passaged compound **15** treated virus S3

Figures S4. Compound **19** exhibited time-dependent disappearance after incubation with human liver microsomes S4

Figures S5. MS^2 spectra of metabolites of compound **19** S4

Figures S6–S41. ^1H NMR and ^{13}C NMR spectra of compounds **3–20** S5-S22

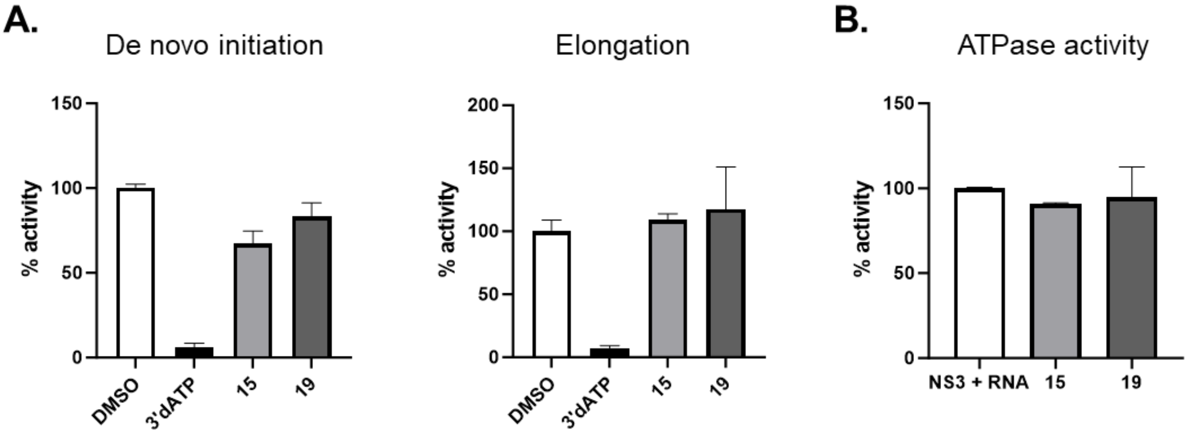

Figure S1. ***In vitro*** **biochemical inhibitory activities of PBTZ compounds 15 and 19. (A)** Compound inhibition against DENV2 NS5 polymerase *de novo* initiation (left panel) or elongation (right panel) activities. **(B)** Compound inhibition against DENV2 NS3 ATPase activity. Bar graphs are presented as percentage enzyme activity with respect to the no compound control tabulated from 2 independent experiments.

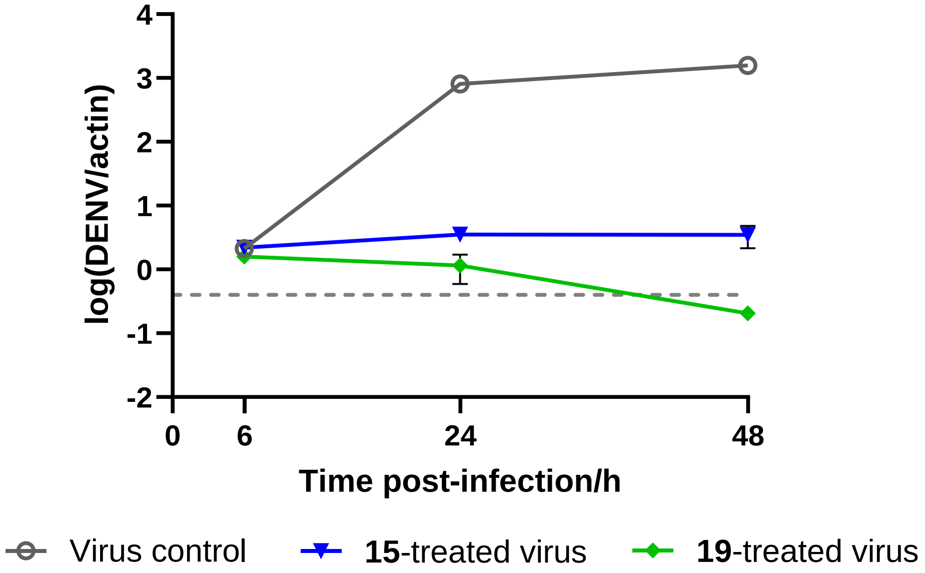

Figure S2. Viral RNA replication profile of the resultant compound-treated DENV-2 on Vero cells. The viral supernatants obtained at 36 h post-infection from the delayed time-of-addition assay (Figure 3C) was used to infect Vero cells and the kinetics of the intracellular viral RNA replication was measured using real-time RT-PCR until 48 h post-infection. Data are presented as mean with standard deviation from 2 independent experiments.

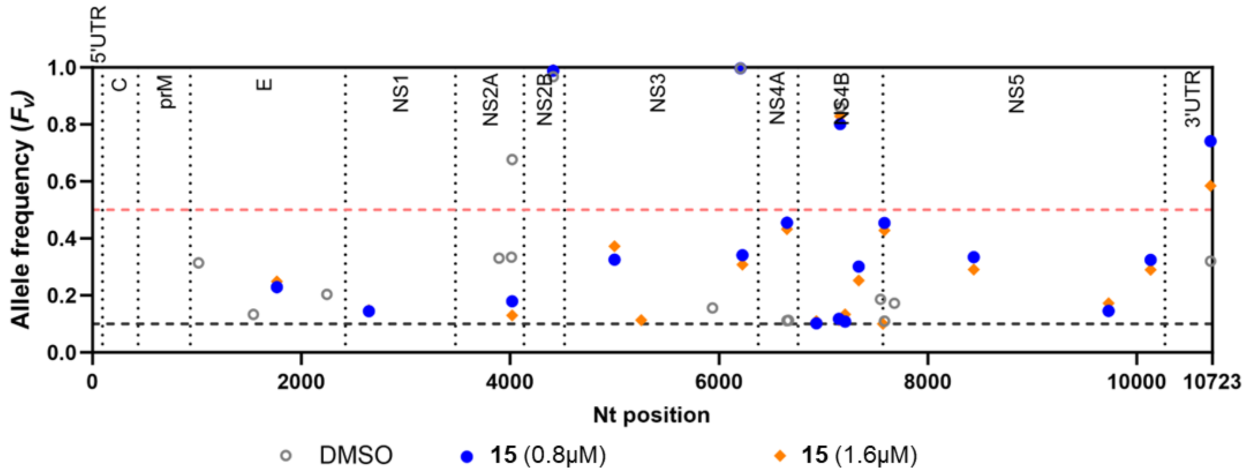

Gene	Nucleotide change	Amino acid change	Mean F_v/%
E	T1766C	L277S	23.9
NS3	C5000T	T159I	35.0
	C5254T	P244S	10.6
	A6224G	K567R	32.5
NS4B	T6931A	S36T	10.6
	C7148T	T108I	11.5
	G7568A	R248K	6.6

Figure S3. Next-generation-sequencing (NGS) of the serially passaged compound 15 treated virus. Vero cells were infected with DENV-2 and treated with increasing concentrations of **15**. The subsequent virus inoculum was serially passaged in Vero cells for 12 passages in the presence of increasing compound concentrations. At the passage 12, virus was subjected to paired-end 2 × 250bp MiSeq (Illumina) to determine low frequency single nucleotide variants (SNVs) using lofreq package.[28] Shown is a scatter plot of all SNVs with variant frequency (F_v) ≥ 10% that are presented in the DMSO-treated (grey open circle symbol) or **15**-treated (blue solid circle or orange solid diamond symbols) viruses plotted along the viral genome. The grey dotted line indicates the threshold F_v of 10% while the red dotted line represents the F_v of 50%. Inset table summarizes the unique non-synonymous SNVs that are only present in the **15**-treated virus and its associated F_v. Data presented is from 2 replicates and the indicated F_v in the inset table is tabulated from variant call analysis performed on the merged replicated alignment files instead of averaging the individual frequencies.

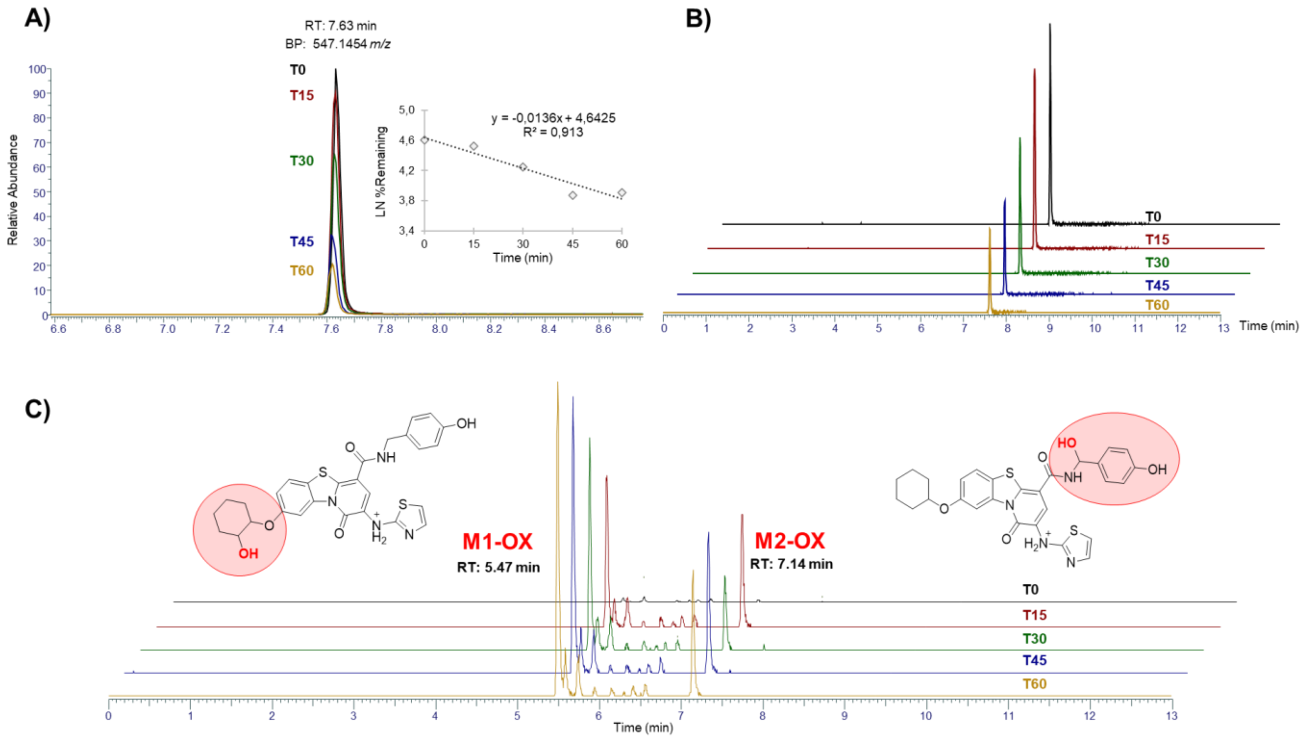

Figure S4. Compound 19 exhibited time-dependent disappearance after incubation with human liver microsomes. (A) zoomed and (B) overlay chromatograms of parent compound; (C) LC-MS profile of the identified metabolites at different time points.

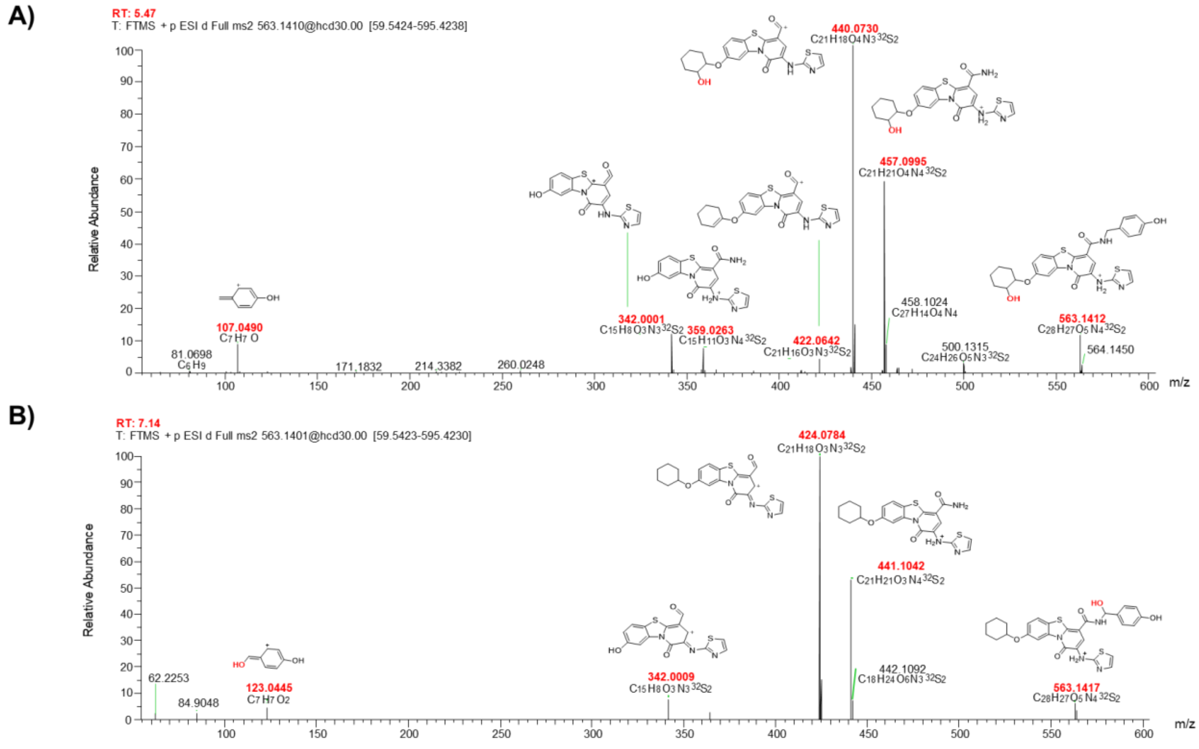

Figure S5. MS^2 spectra illustrating the fragmentation pathways for M1-OX (A), and M2-OX (B) metabolites of compound **19**.

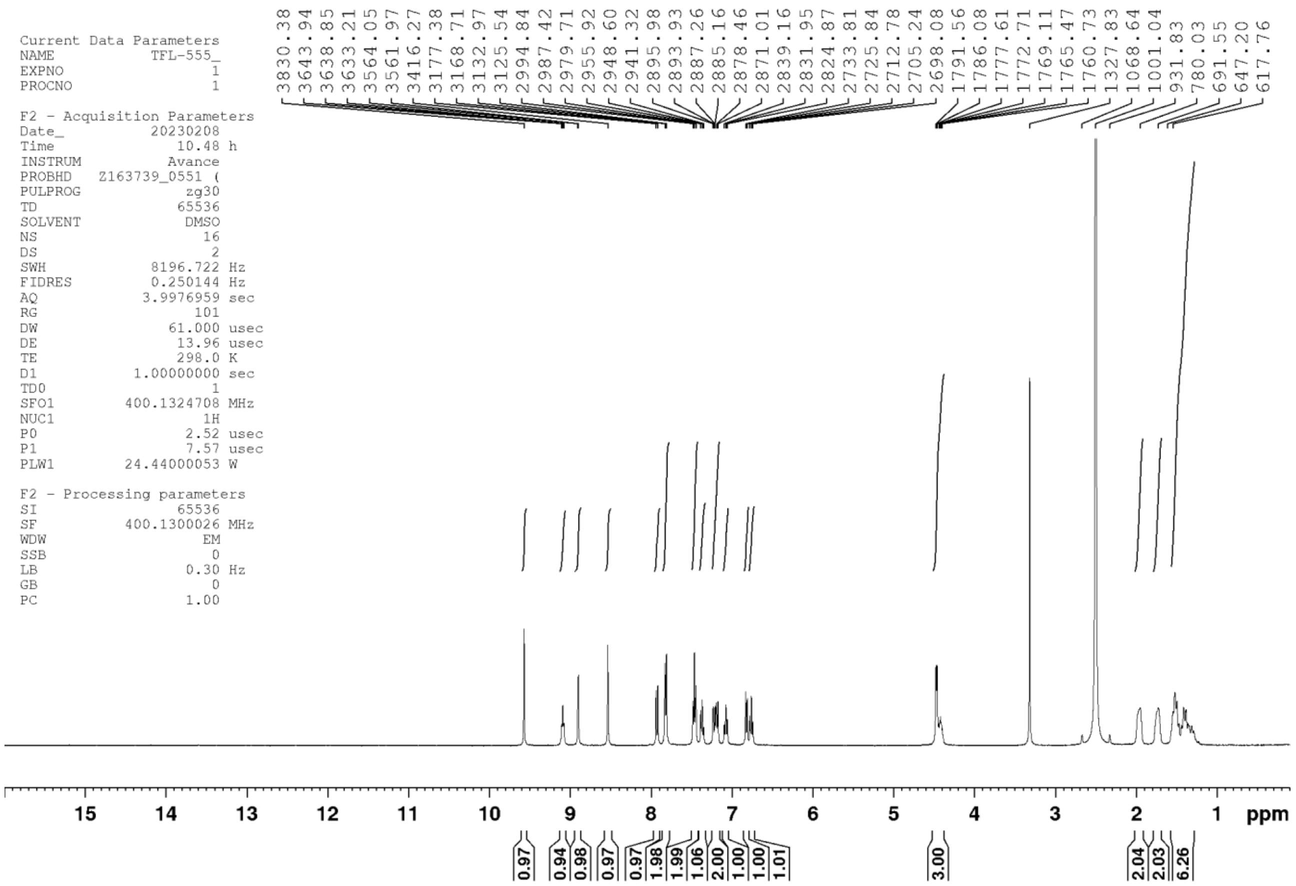

Figure S6. ^{1}H NMR spectrum of compound **3**.

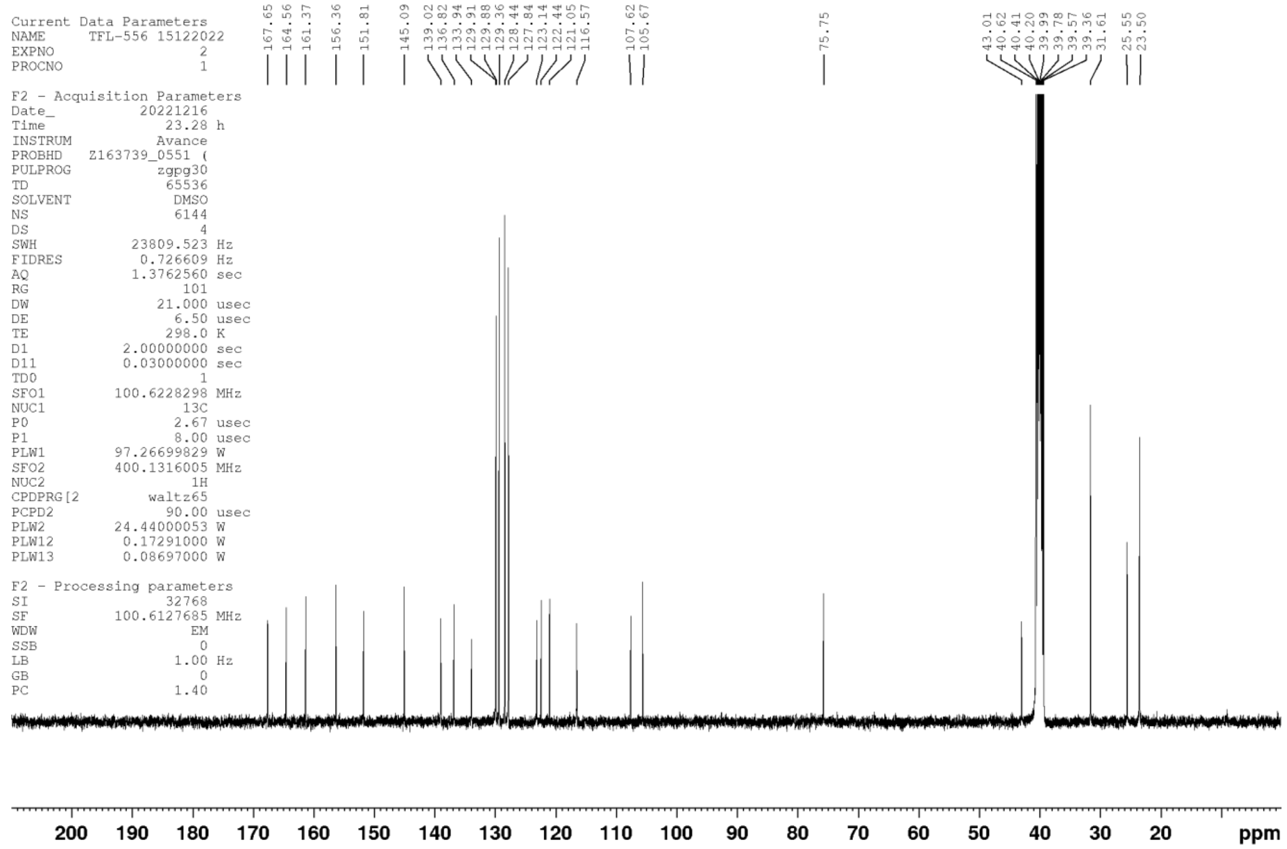

Figure S7. ^{13}C NMR spectrum of compound **3**.

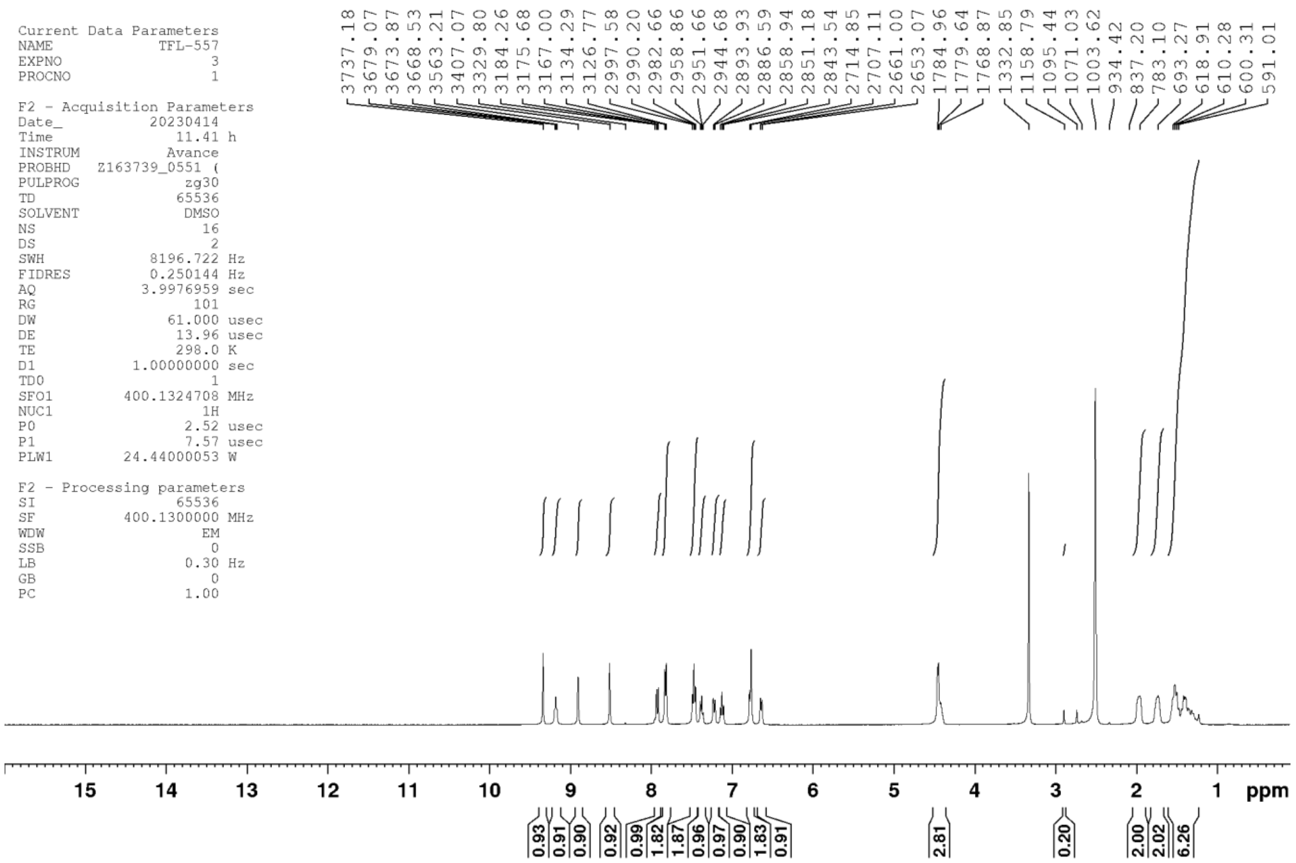

Figure S8. ^{1}H NMR spectrum of compound **4**.

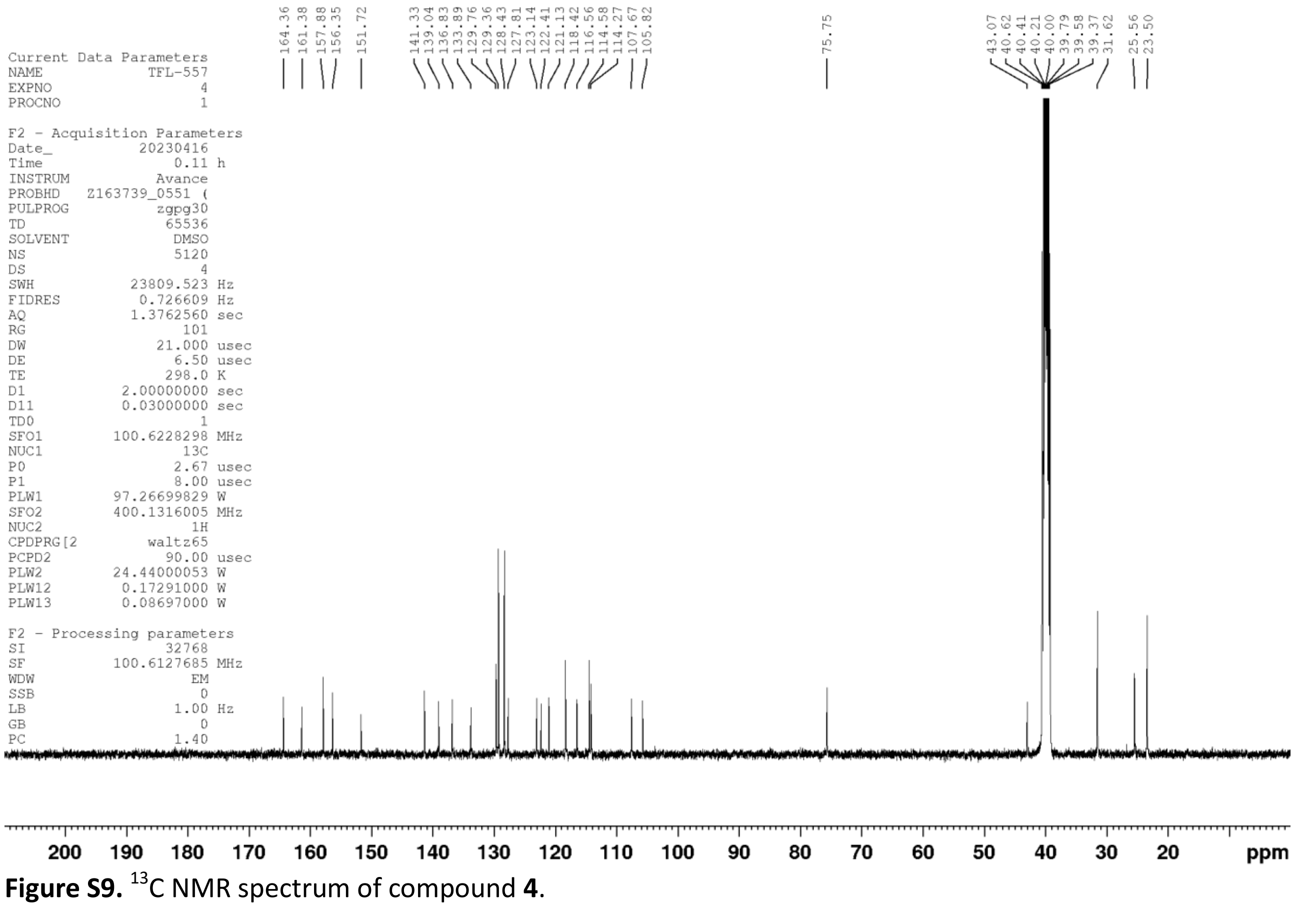

Figure S9. ^{13}C NMR spectrum of compound **4**.

Figure S10. ^{1}H NMR spectrum of compound **5**.

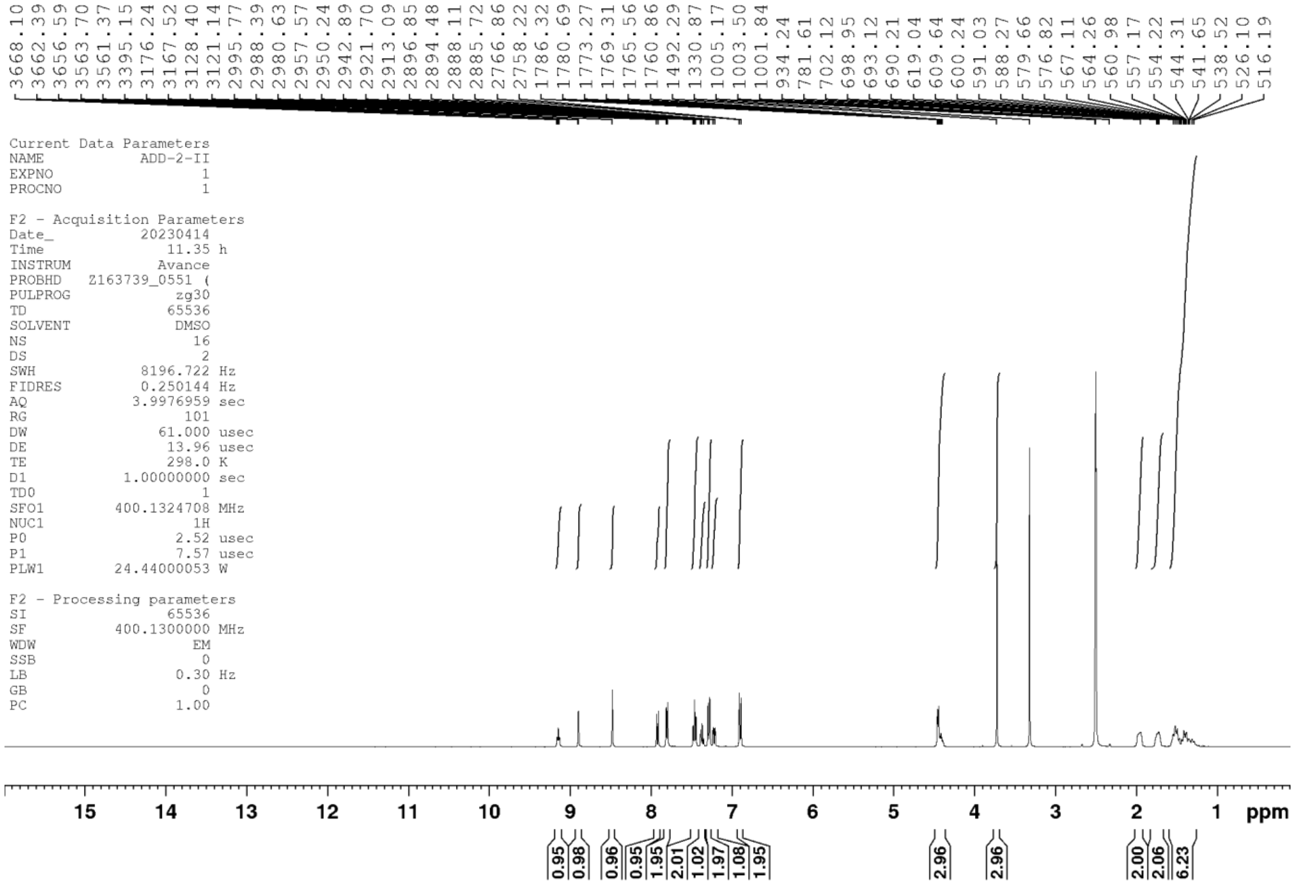

Figure S11. ^{1}H NMR spectrum of compound **5**.

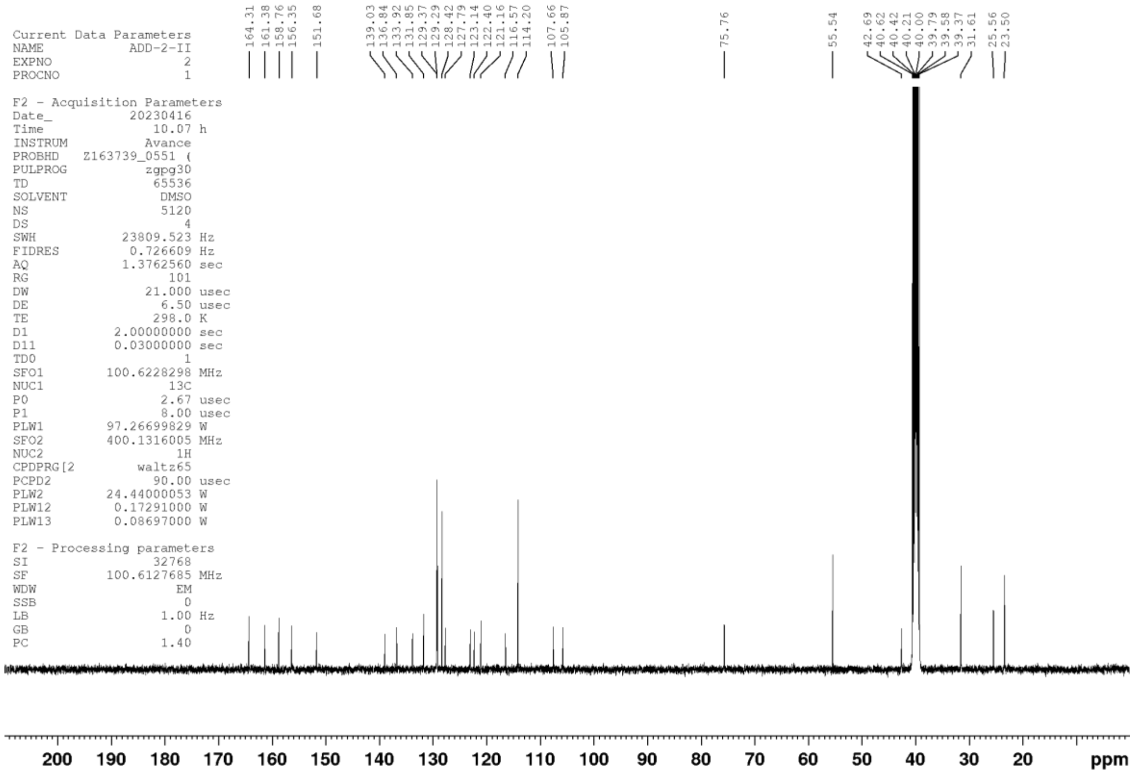

Figure S12. ^{13}C NMR spectrum of compound **5**.

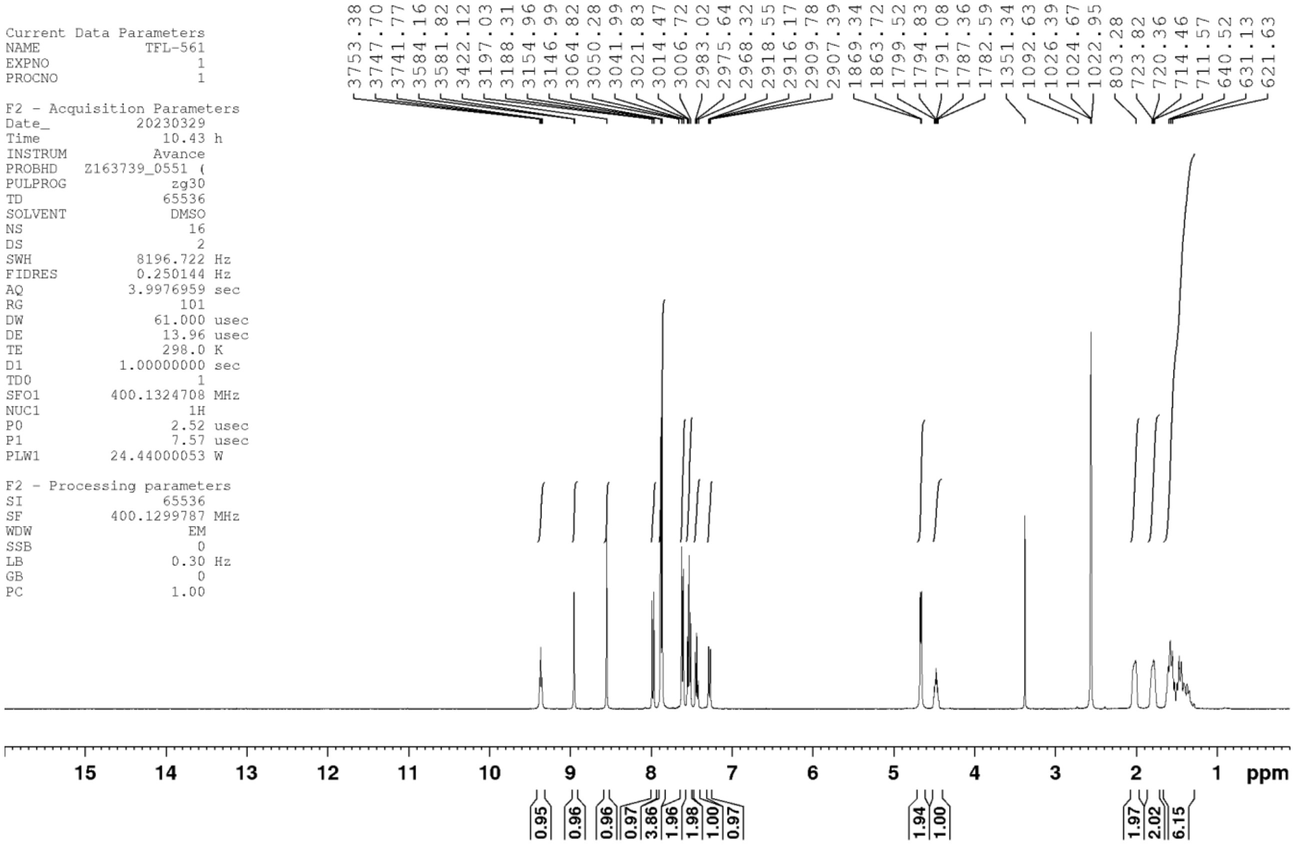

Figure S13. ^{1}H NMR spectrum of compound **6**.

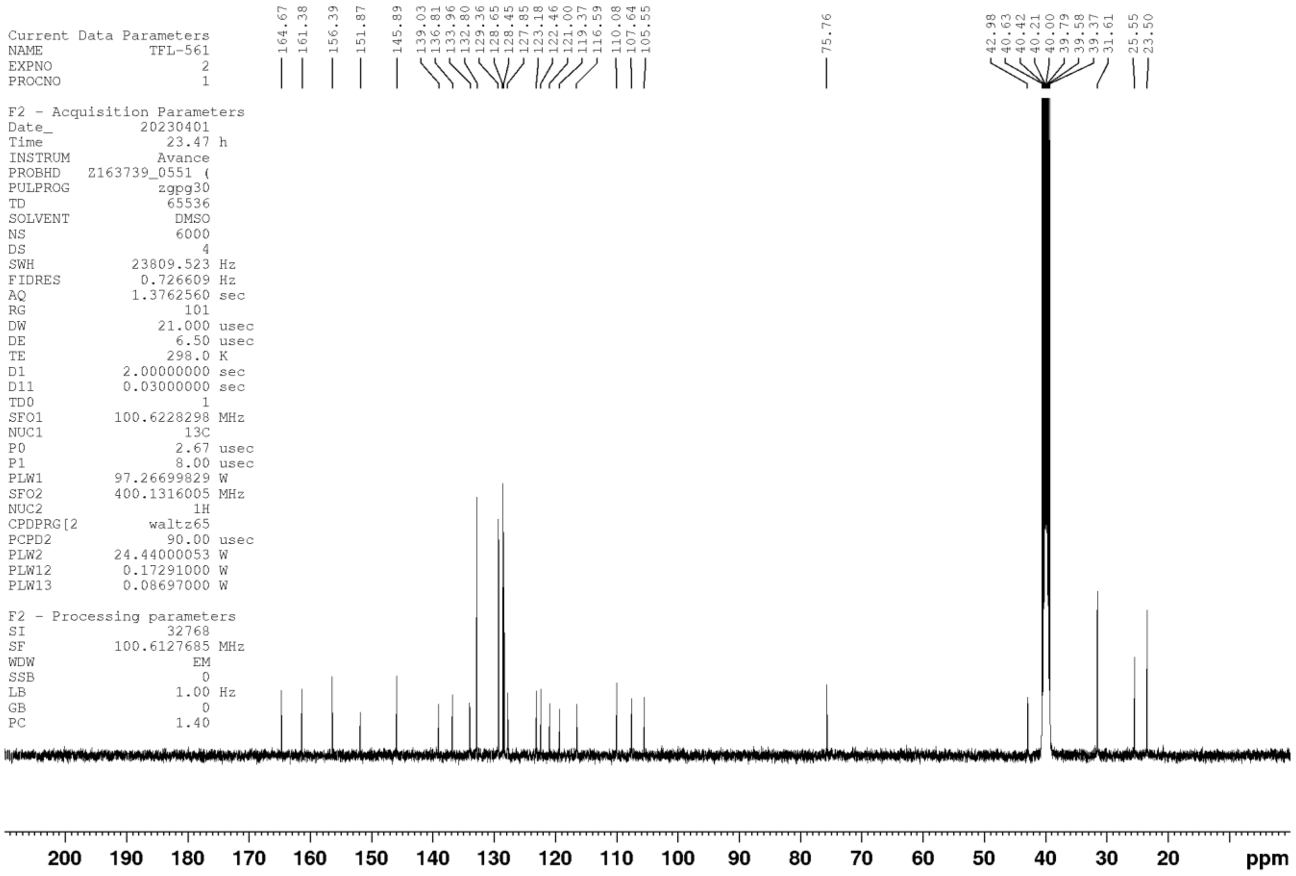

Figure S14. ^{13}C NMR spectrum of compound **6**.

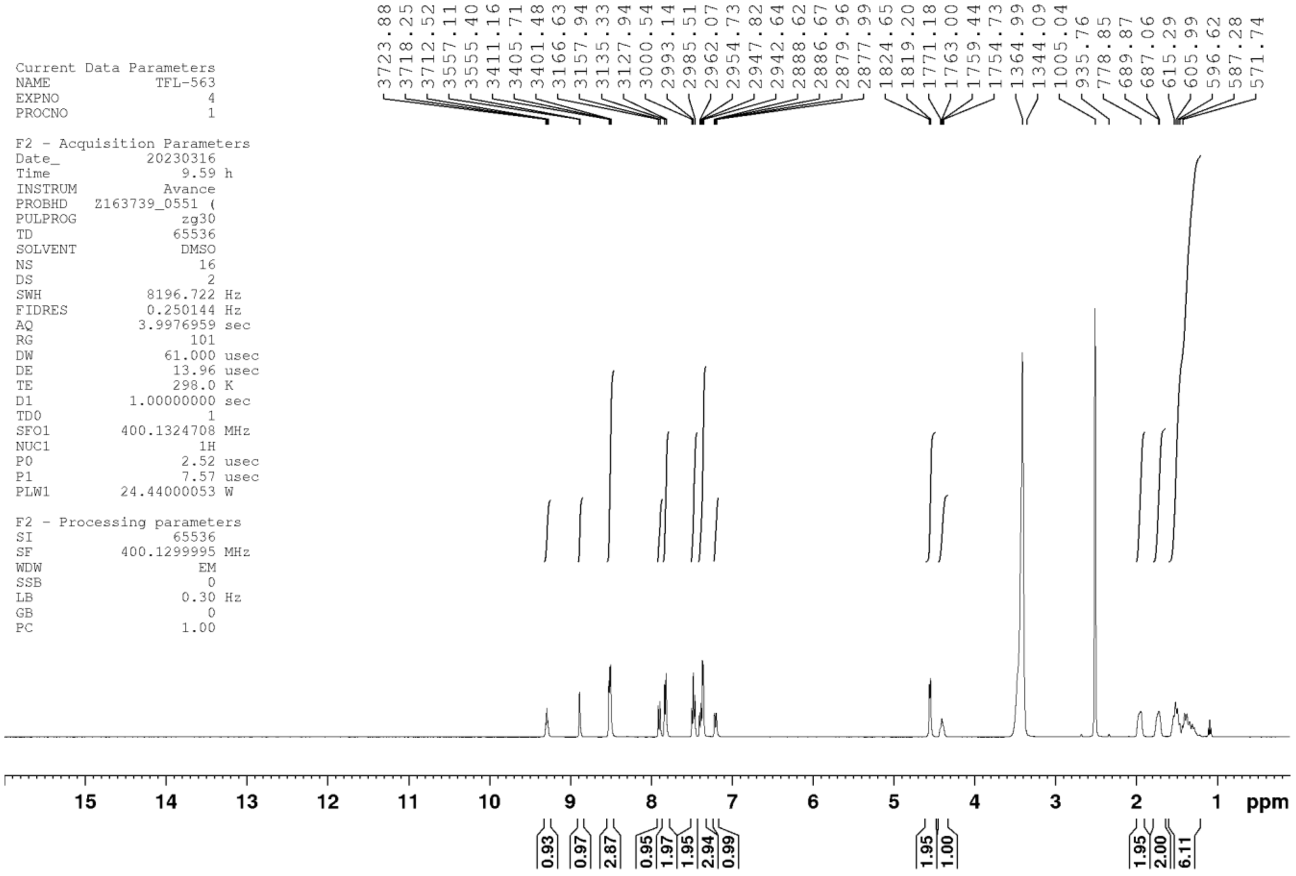

Figure S15. ^{1}H NMR spectrum of compound **7**.

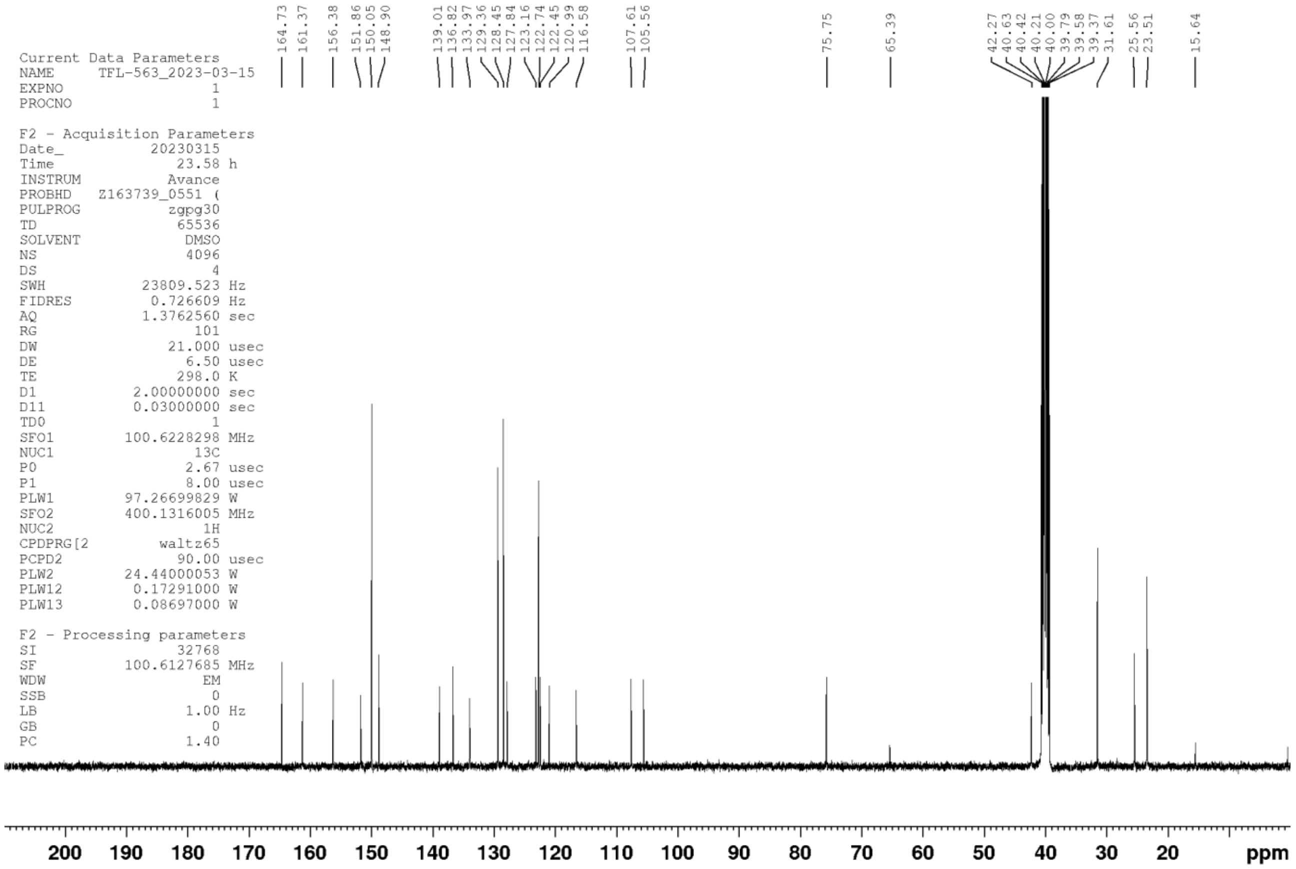

Figure S16. ^{13}C NMR spectrum of compound **7**.

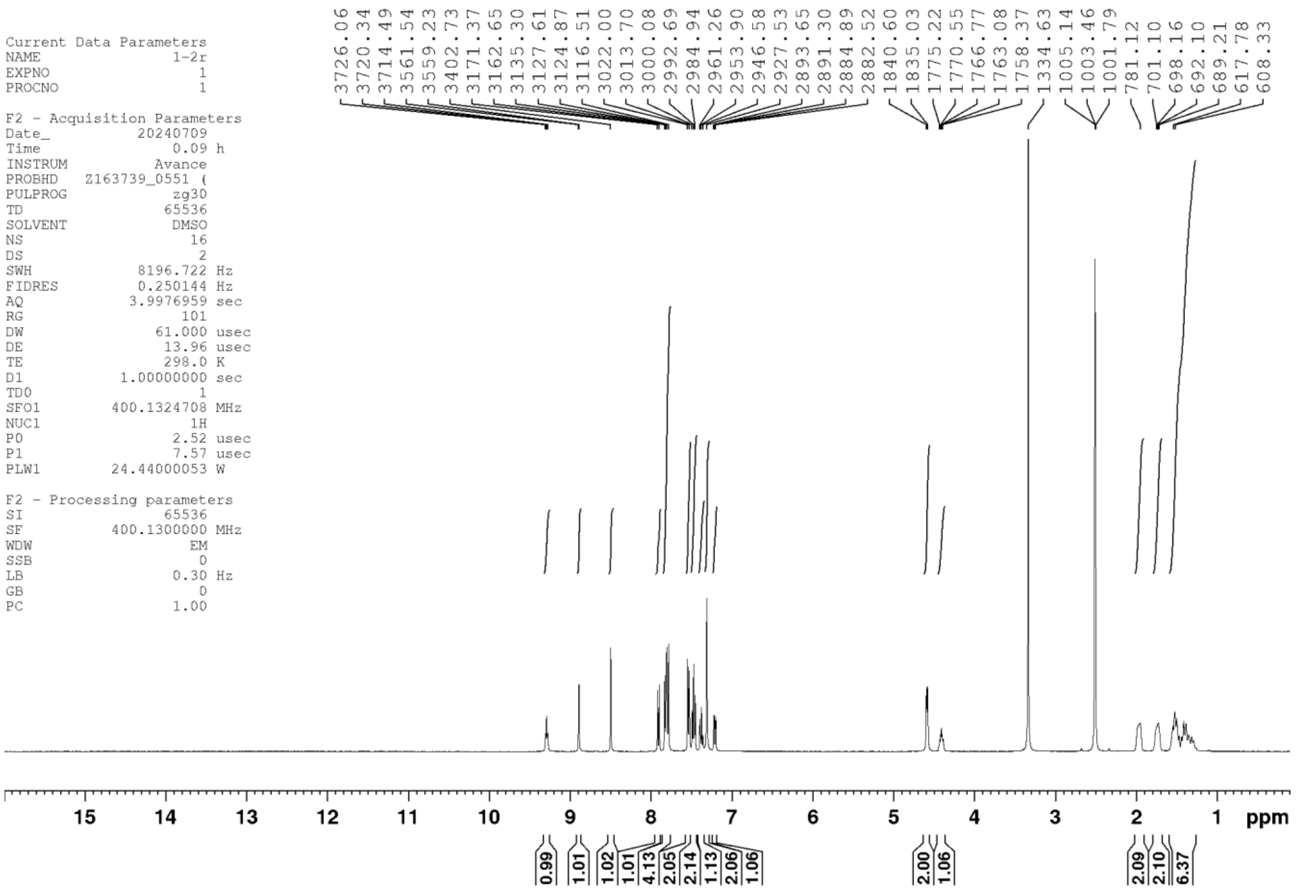

Figure S17. ^{1}H NMR spectrum of compound **8**.

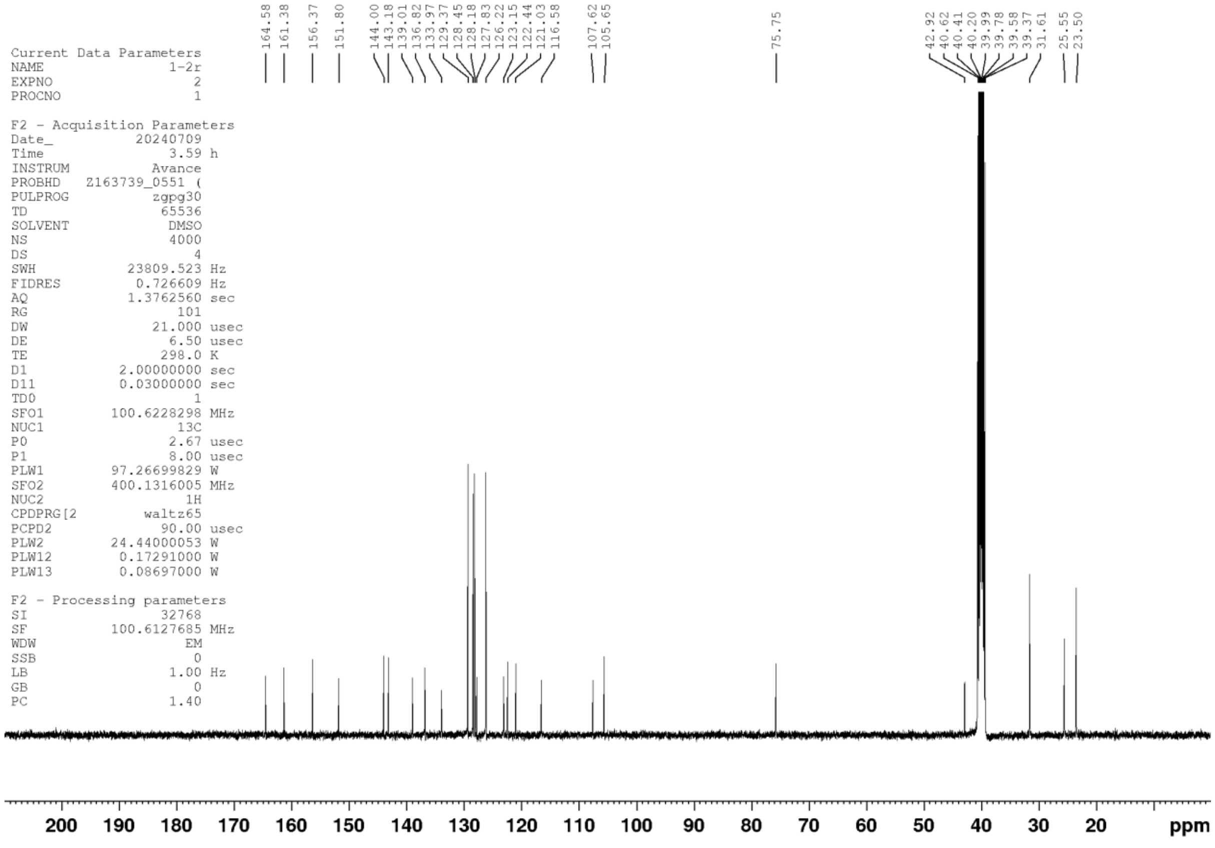

Figure S18. ^{13}C NMR spectrum of compound **8**.

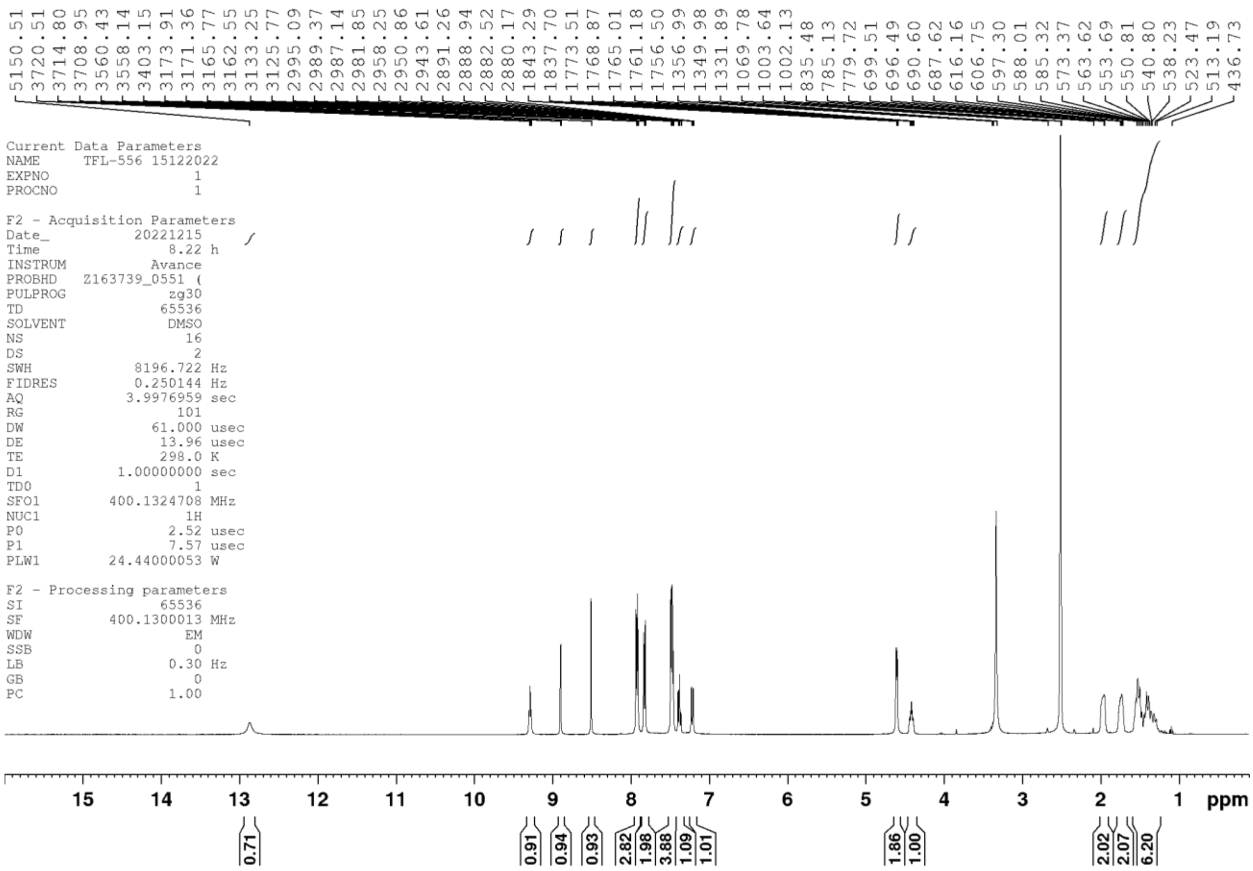

Figure S19. ^{1}H NMR spectrum of compound **9**.

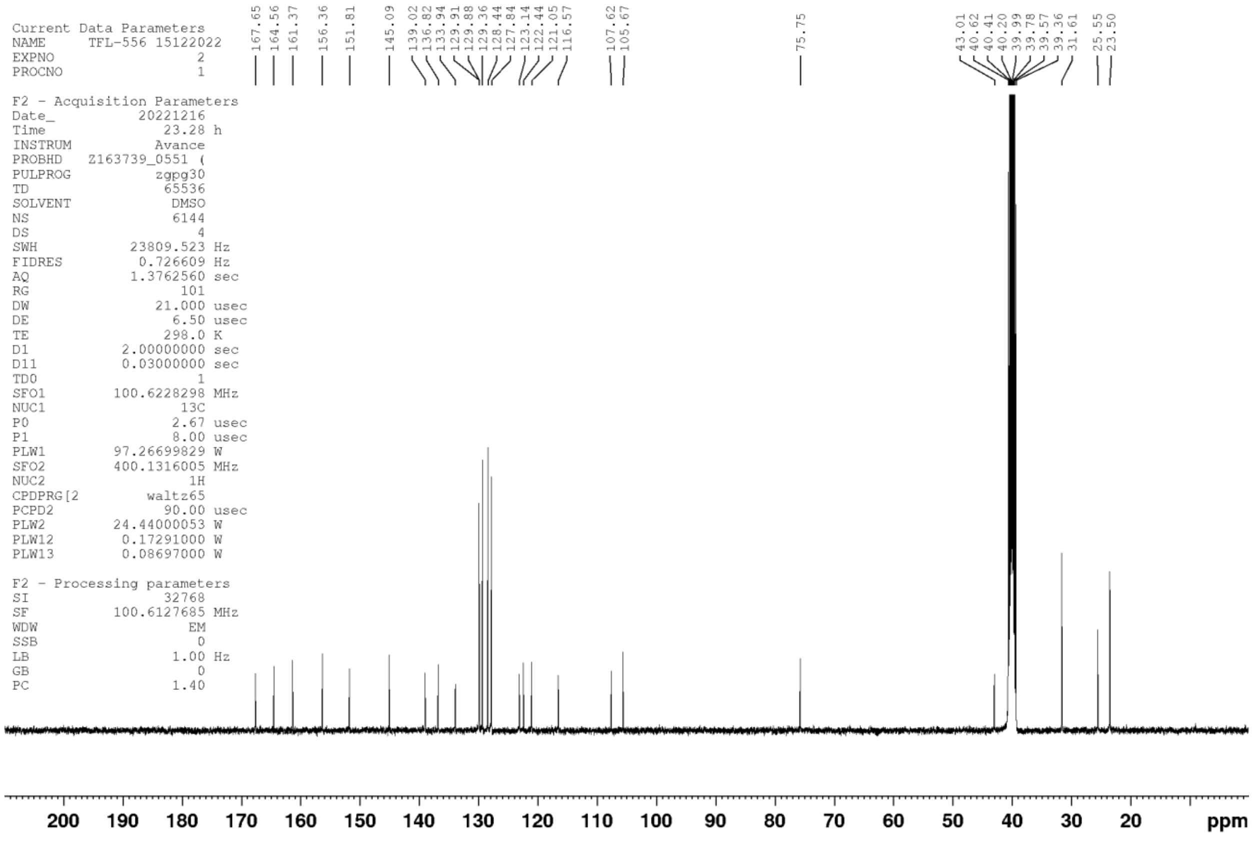

Figure S20. ^{13}C NMR spectrum of compound **9**.

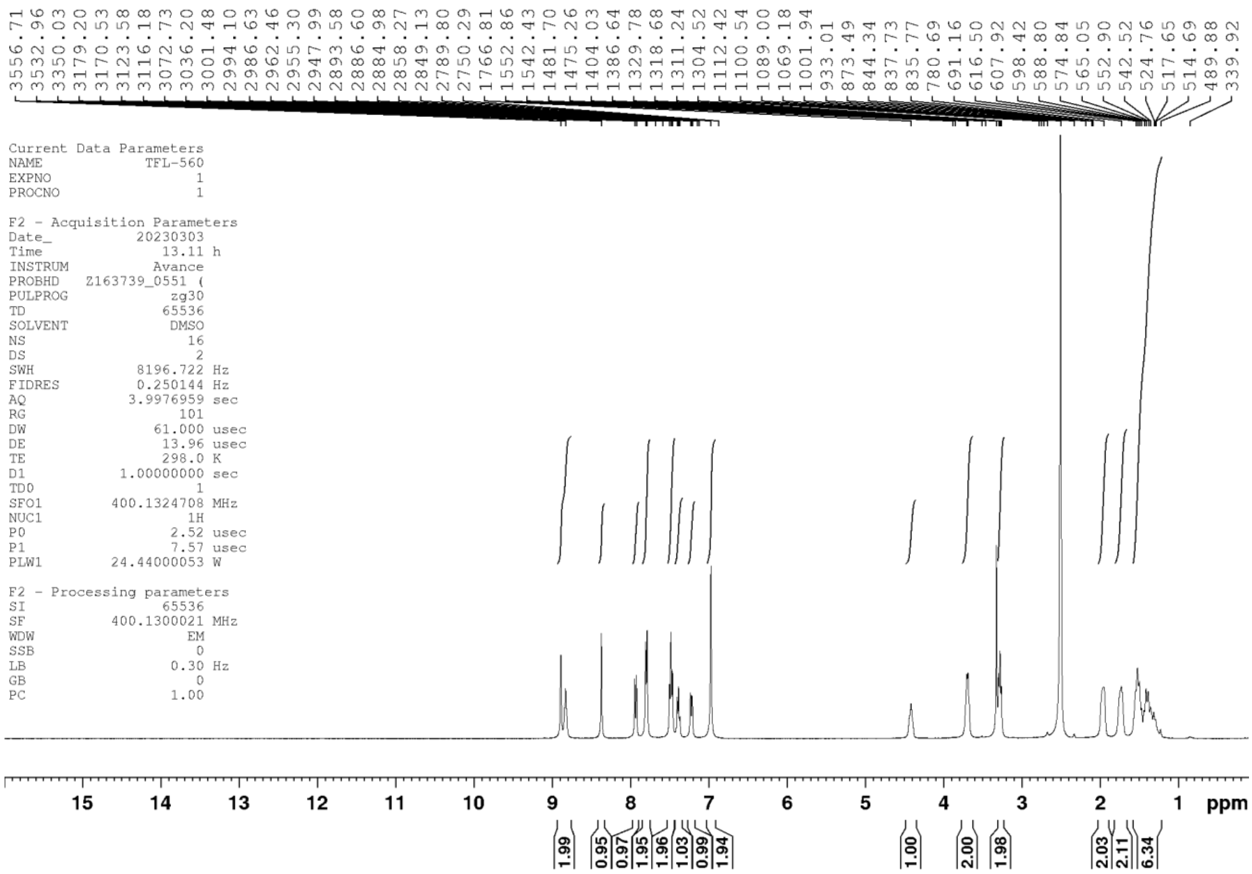

Figure S21. ^{1}H NMR spectrum of compound **10**.

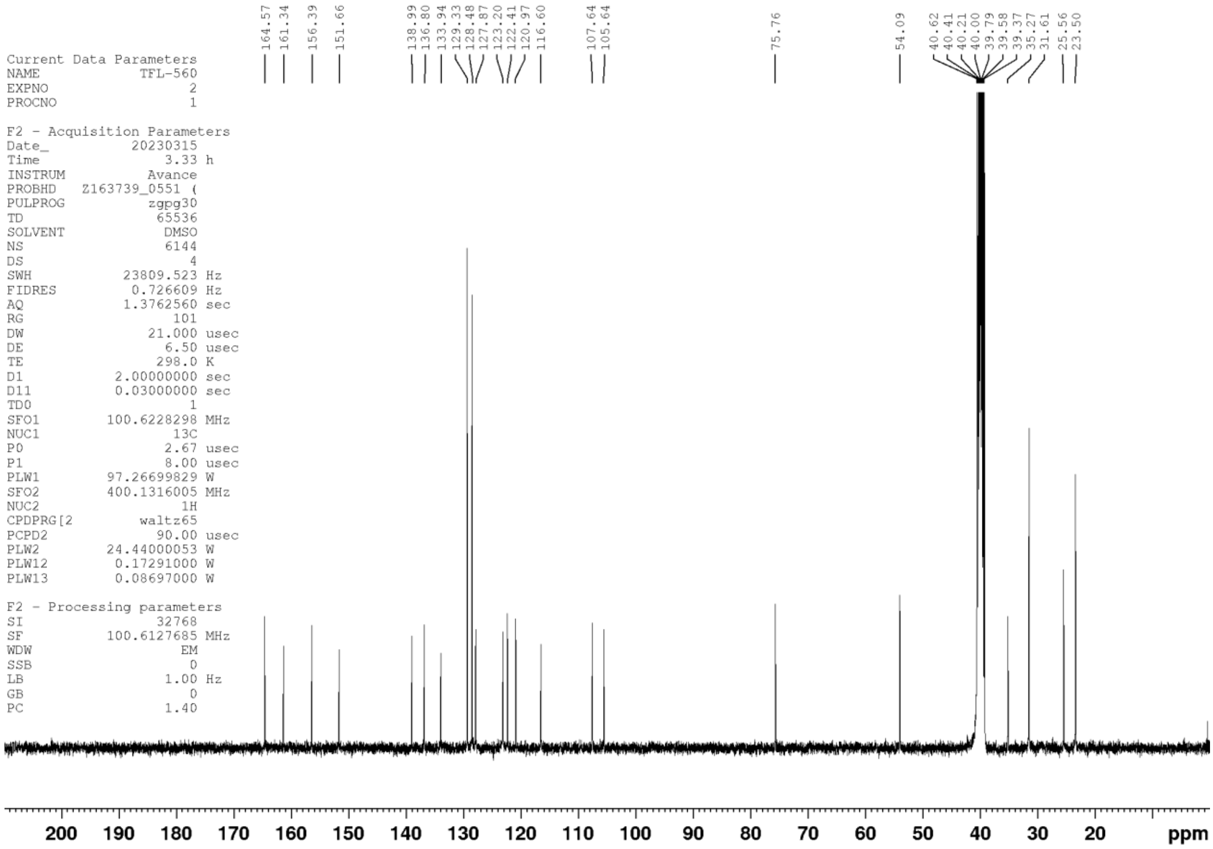

Figure S22. ^{13}C NMR spectrum of compound **10**.

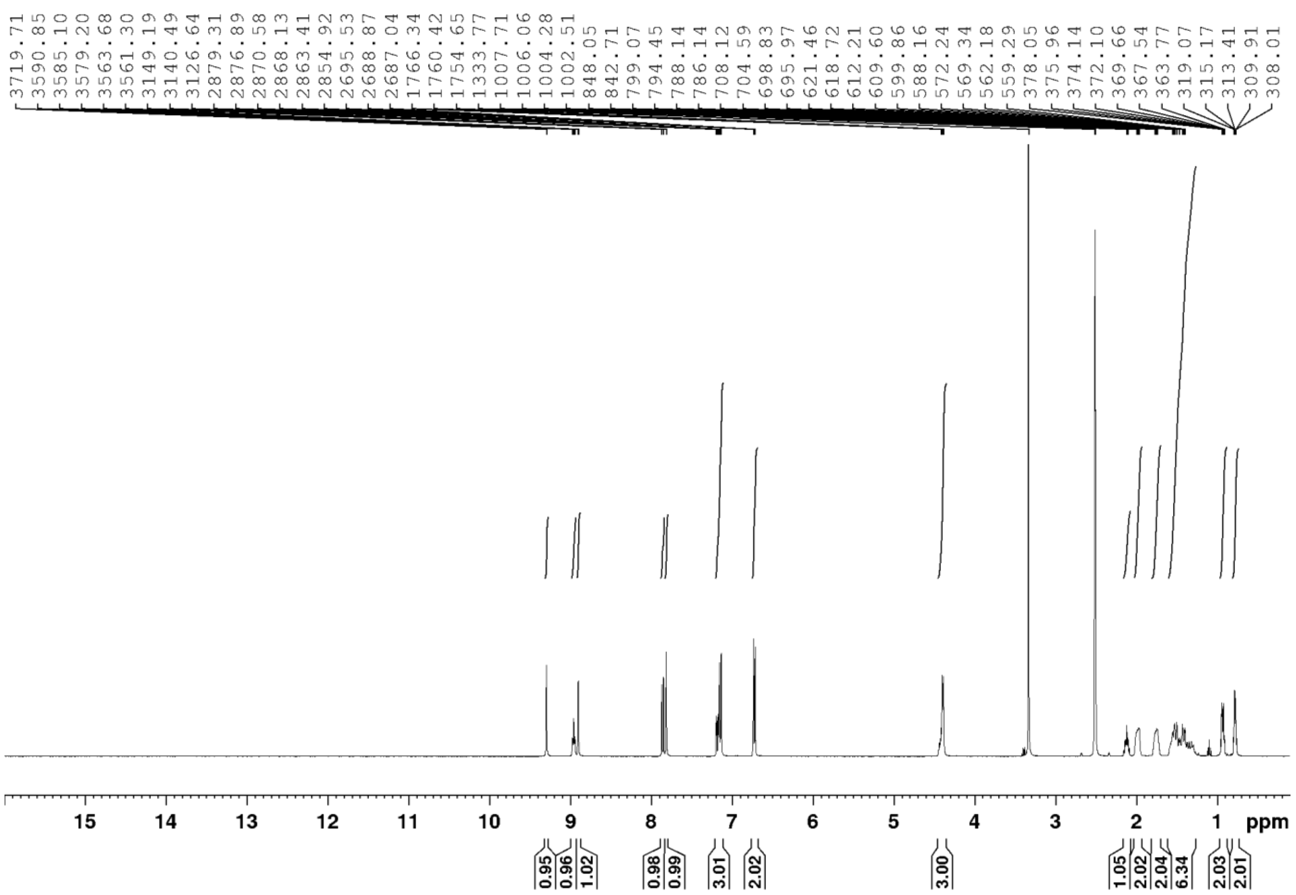

Figure S23. ^{1}H NMR spectrum of compound **11**.

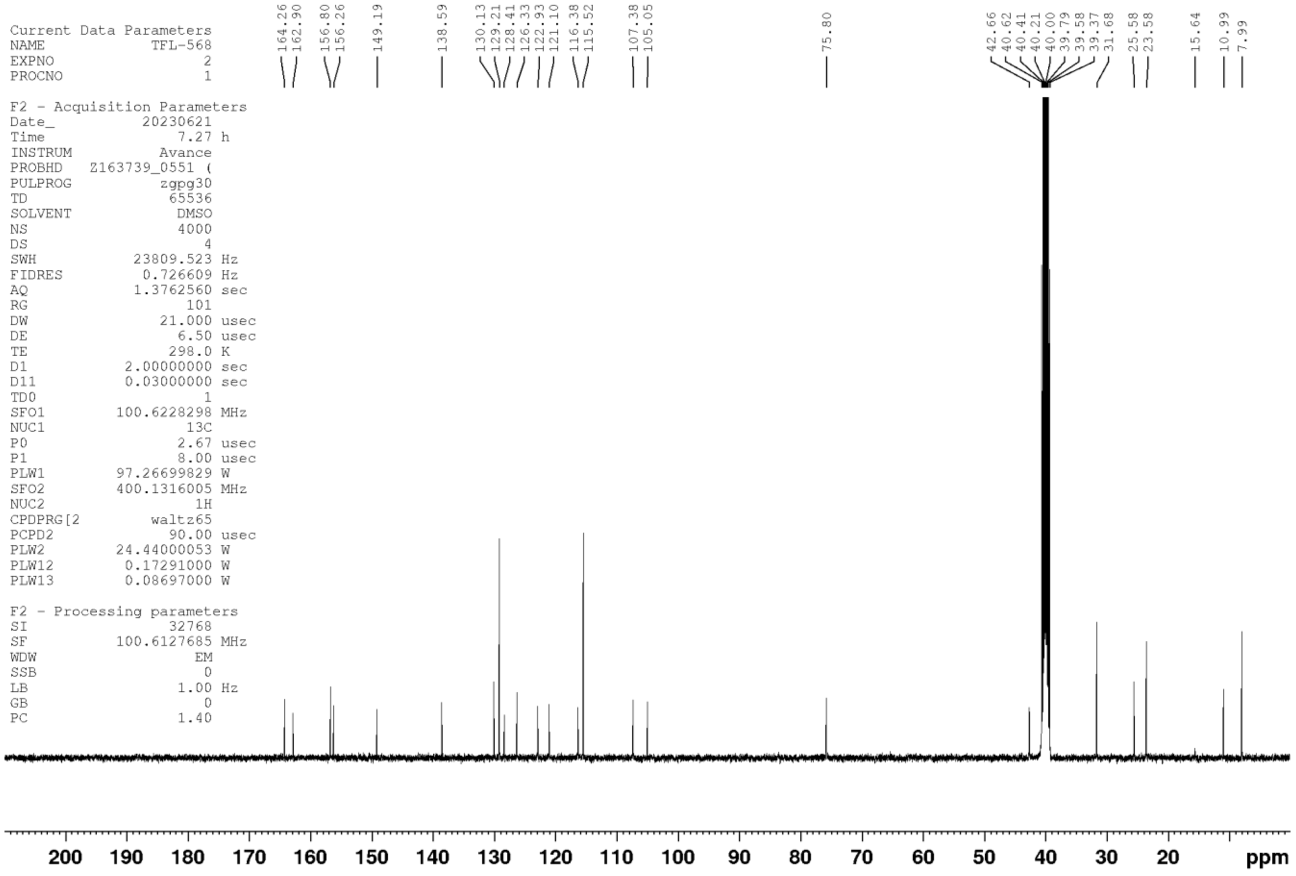

Figure S24. ^{13}C NMR spectrum of compound **11**.

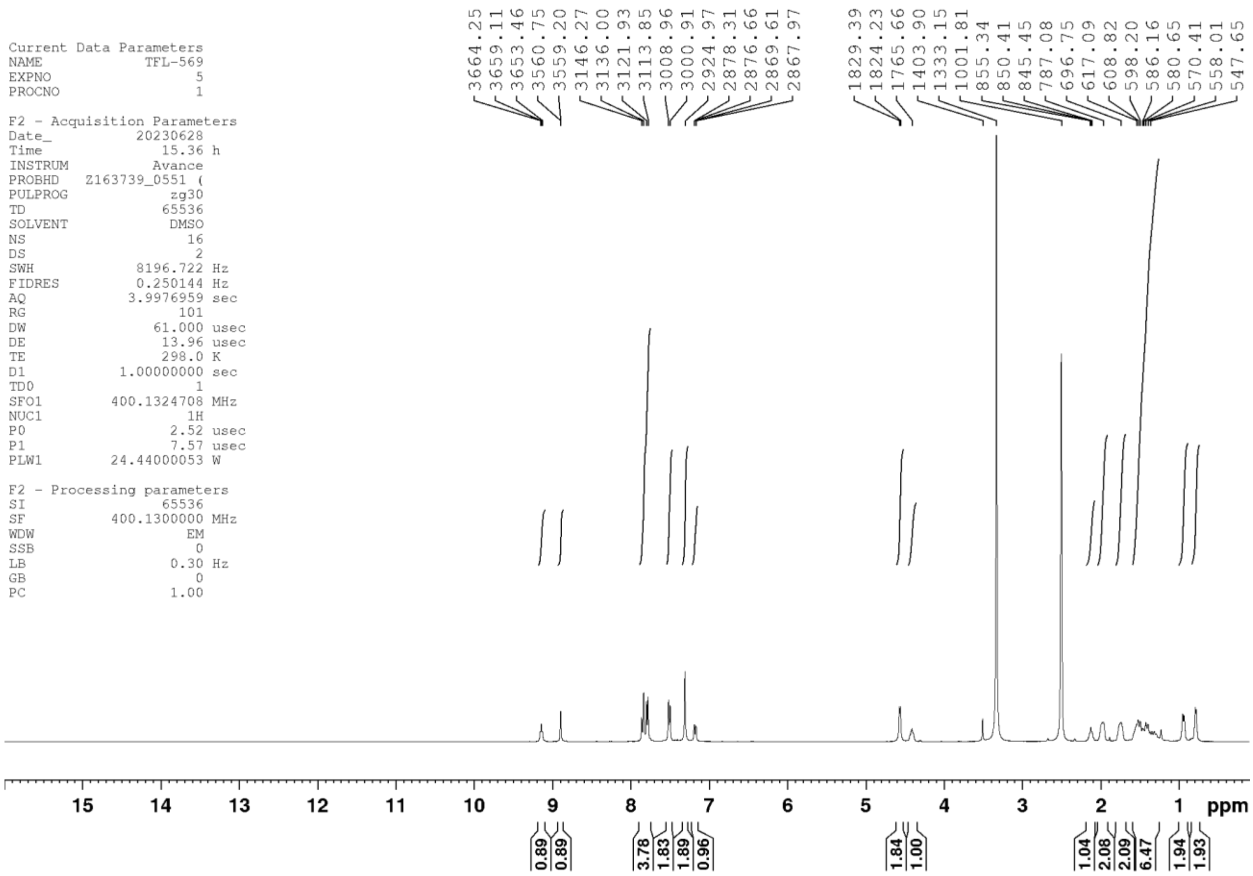

Figure S25. ^{1}H NMR spectrum of compound **12**.

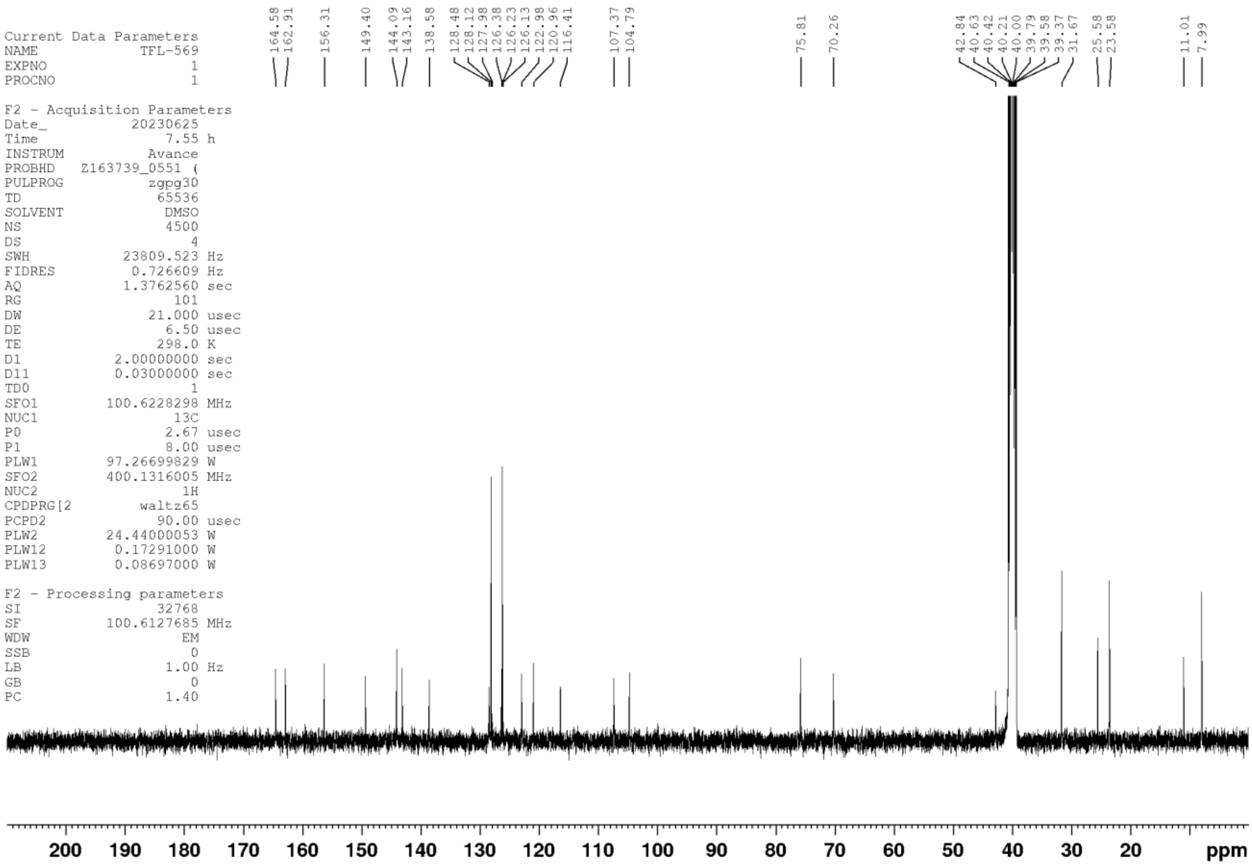

Figure S26. ^{13}C NMR spectrum of compound **12**.

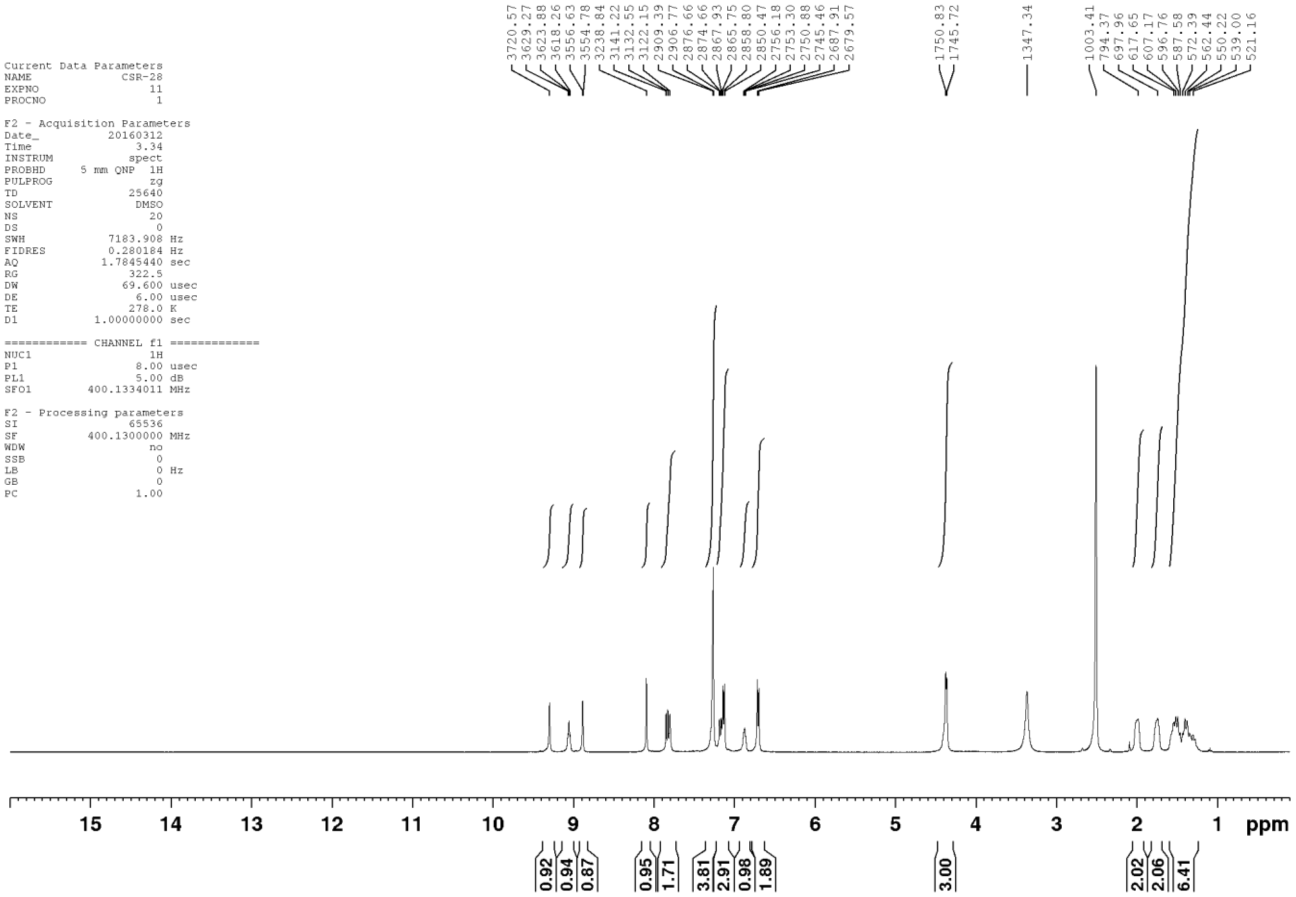

Figure S27. ^{1}H NMR spectrum of compound **13**.

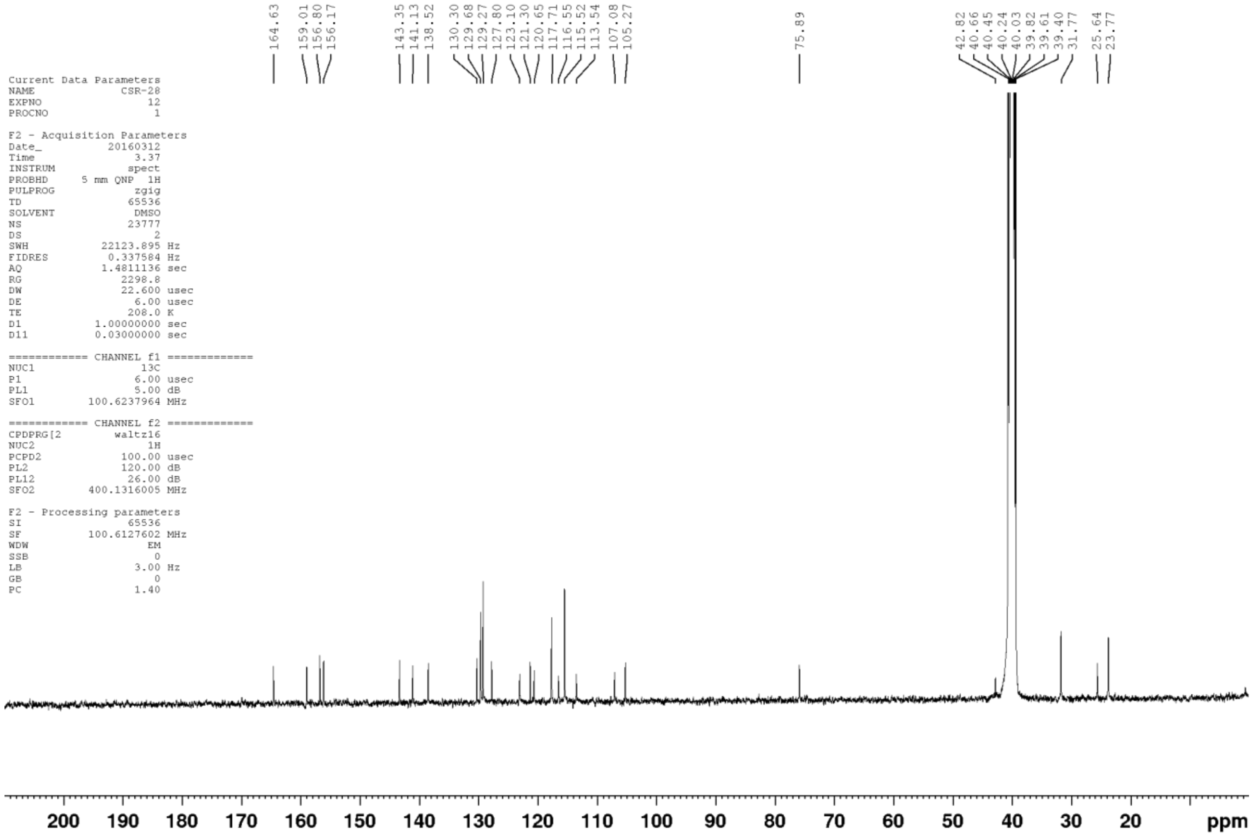

Figure S28. ^{13}C NMR spectrum of compound **13**.

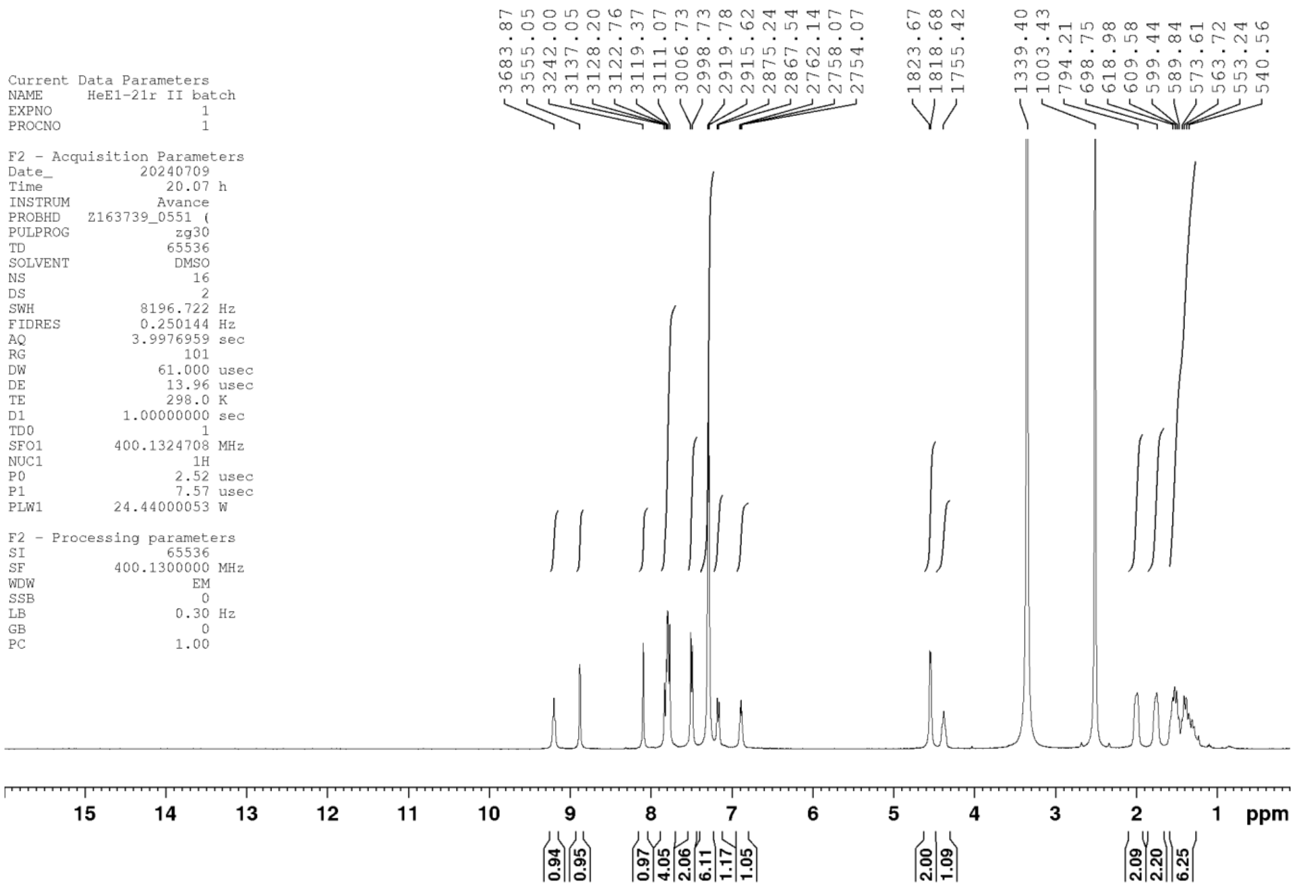

Figure S29. ^{1}H NMR spectrum of compound **14**.

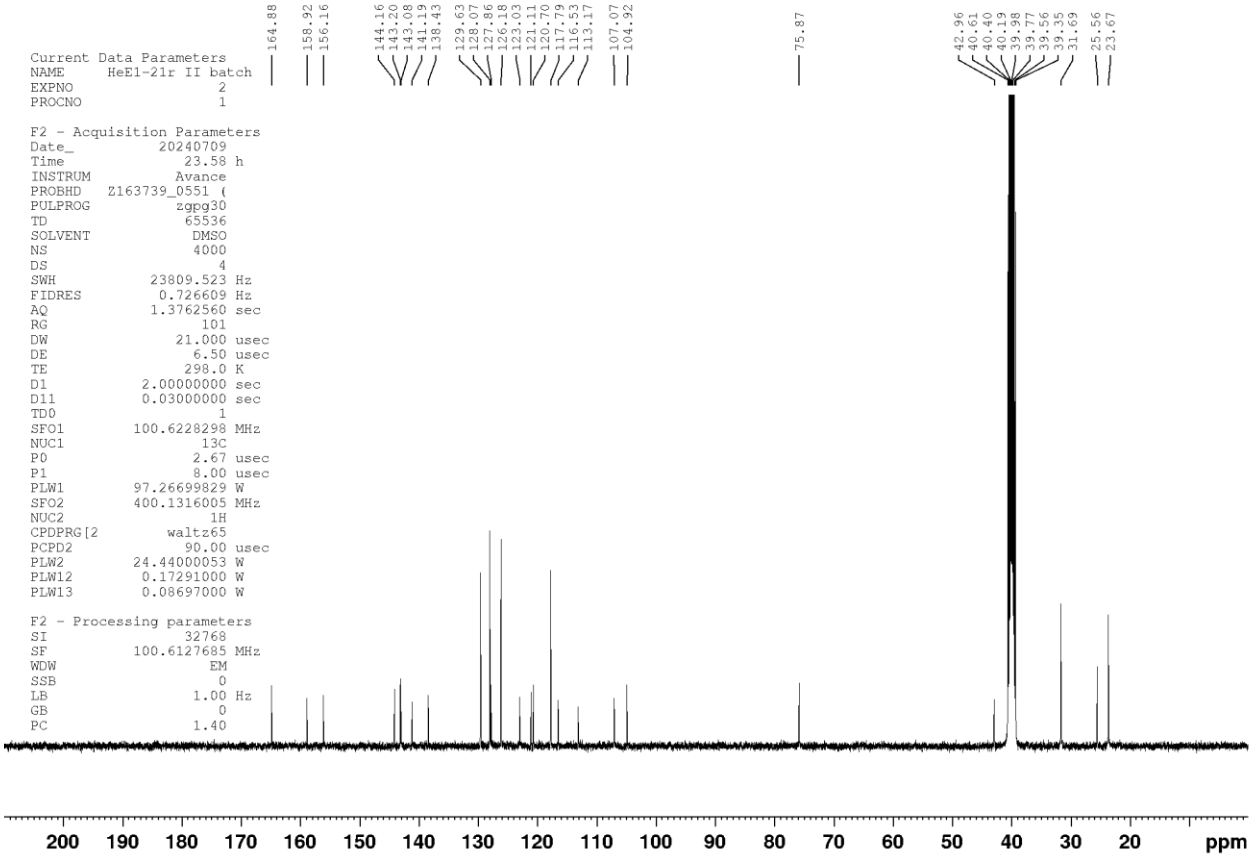

Figure S30. ^{13}C NMR spectrum of compound **14**.

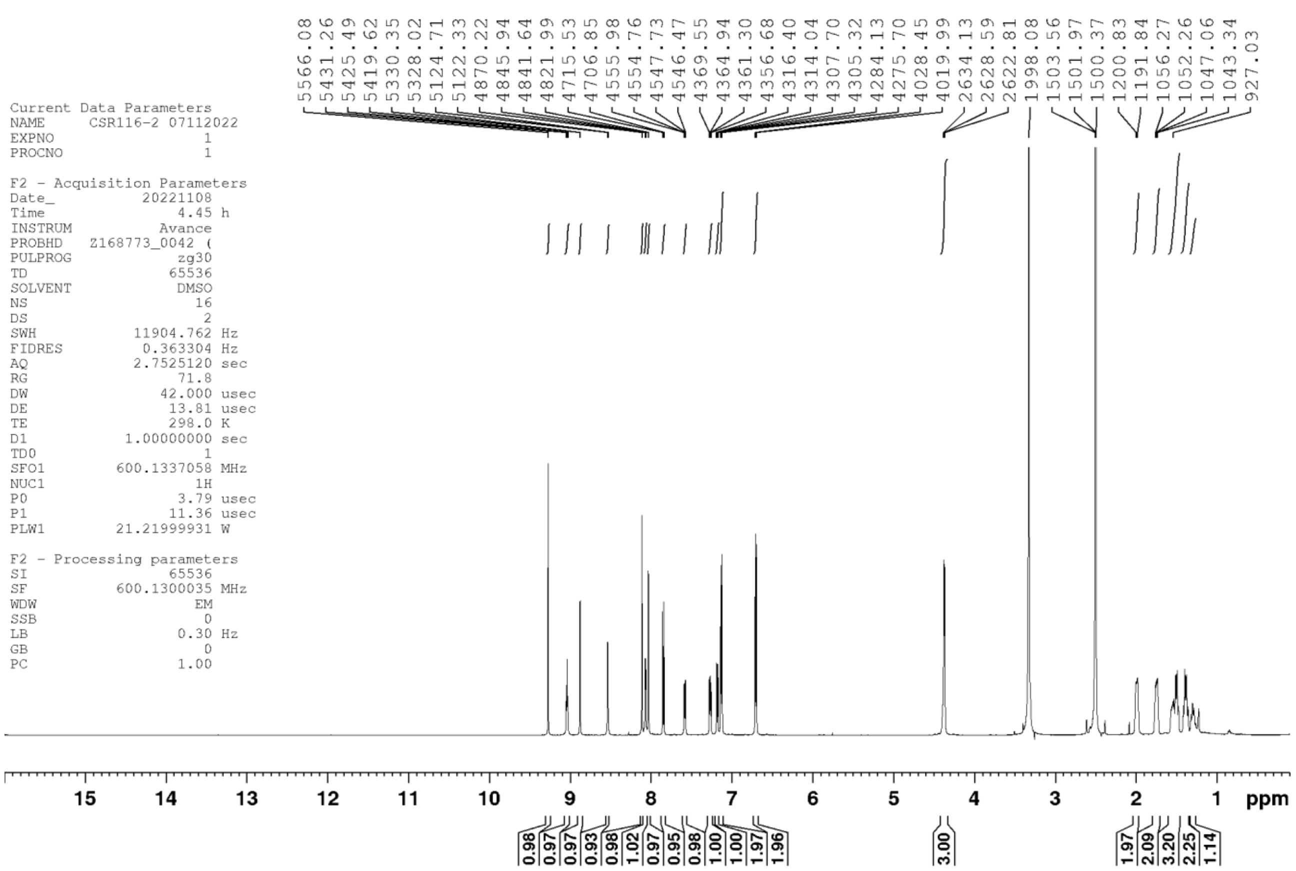

Figure S31. ^{1}H NMR spectrum of compound **15**.

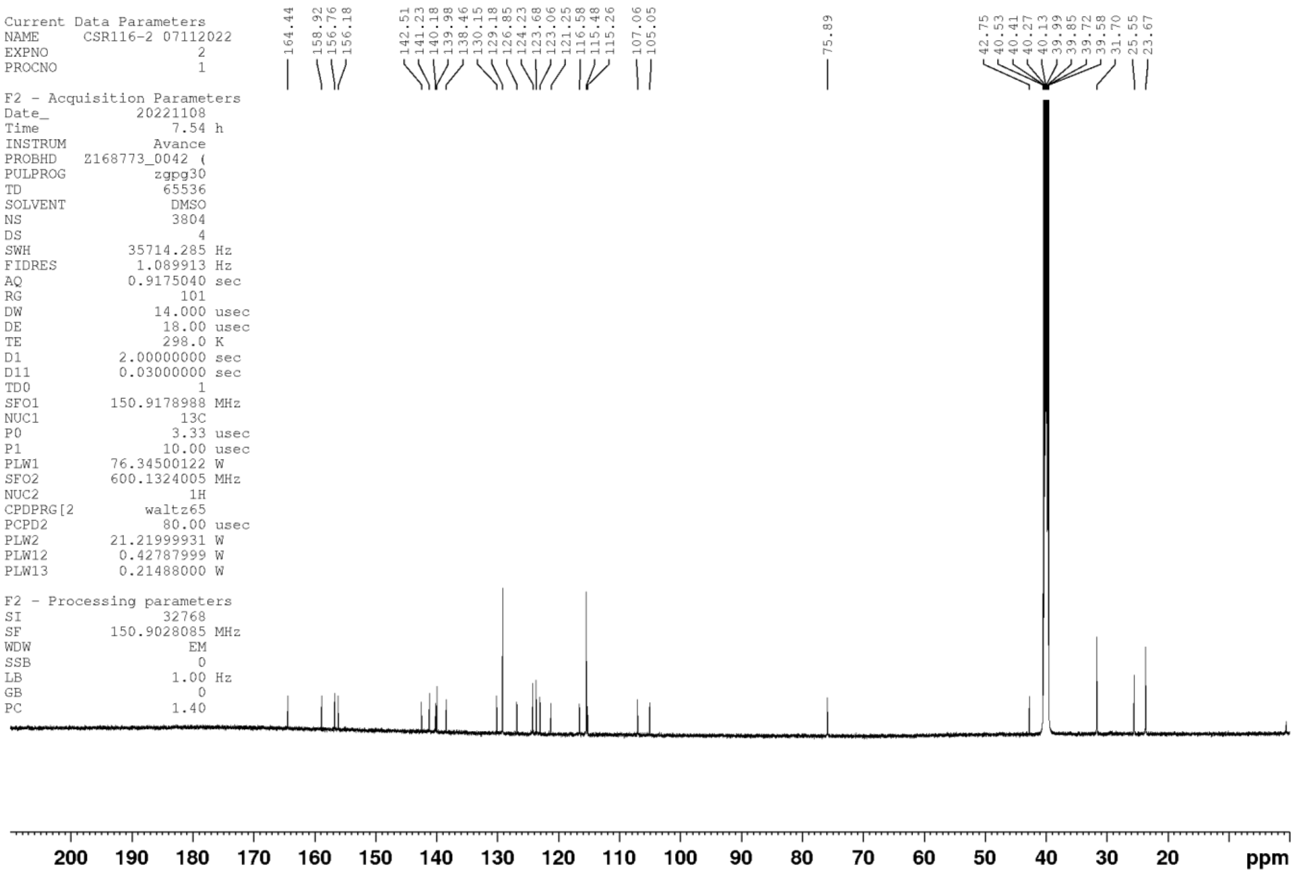

Figure S32. ^{13}C NMR spectrum of compound **15**.

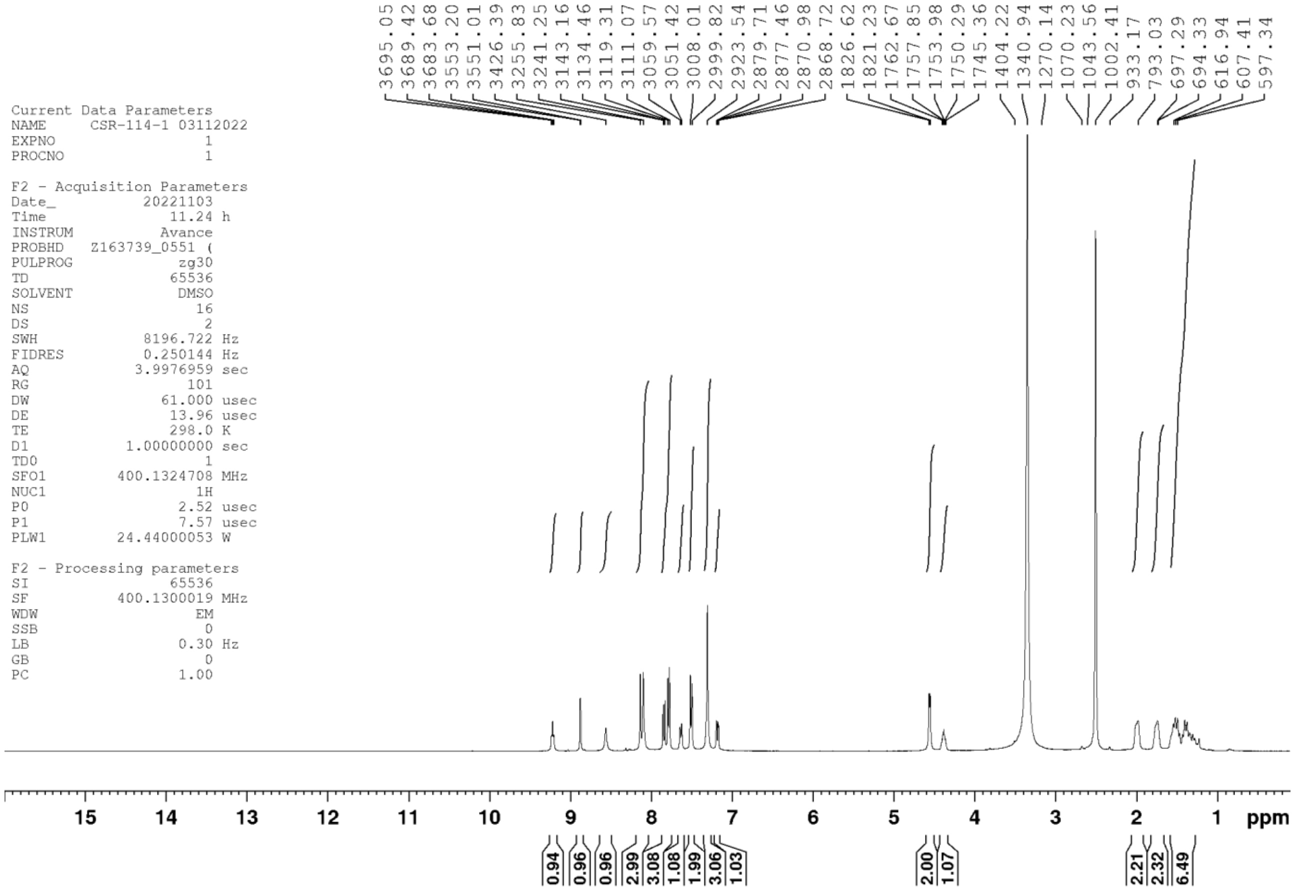

Figure S33. ^{1}H NMR spectrum of compound **16**.

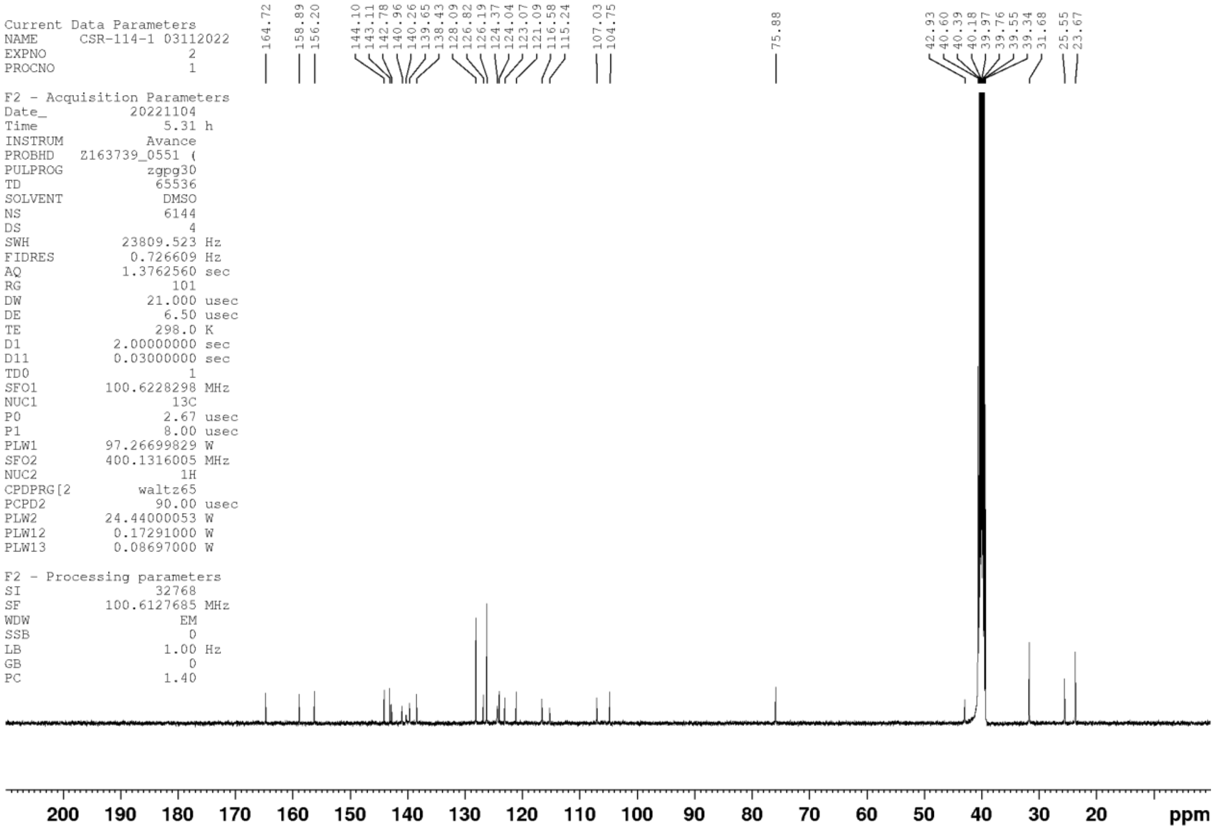

Figure S34. ^{13}C NMR spectrum of compound **16**.

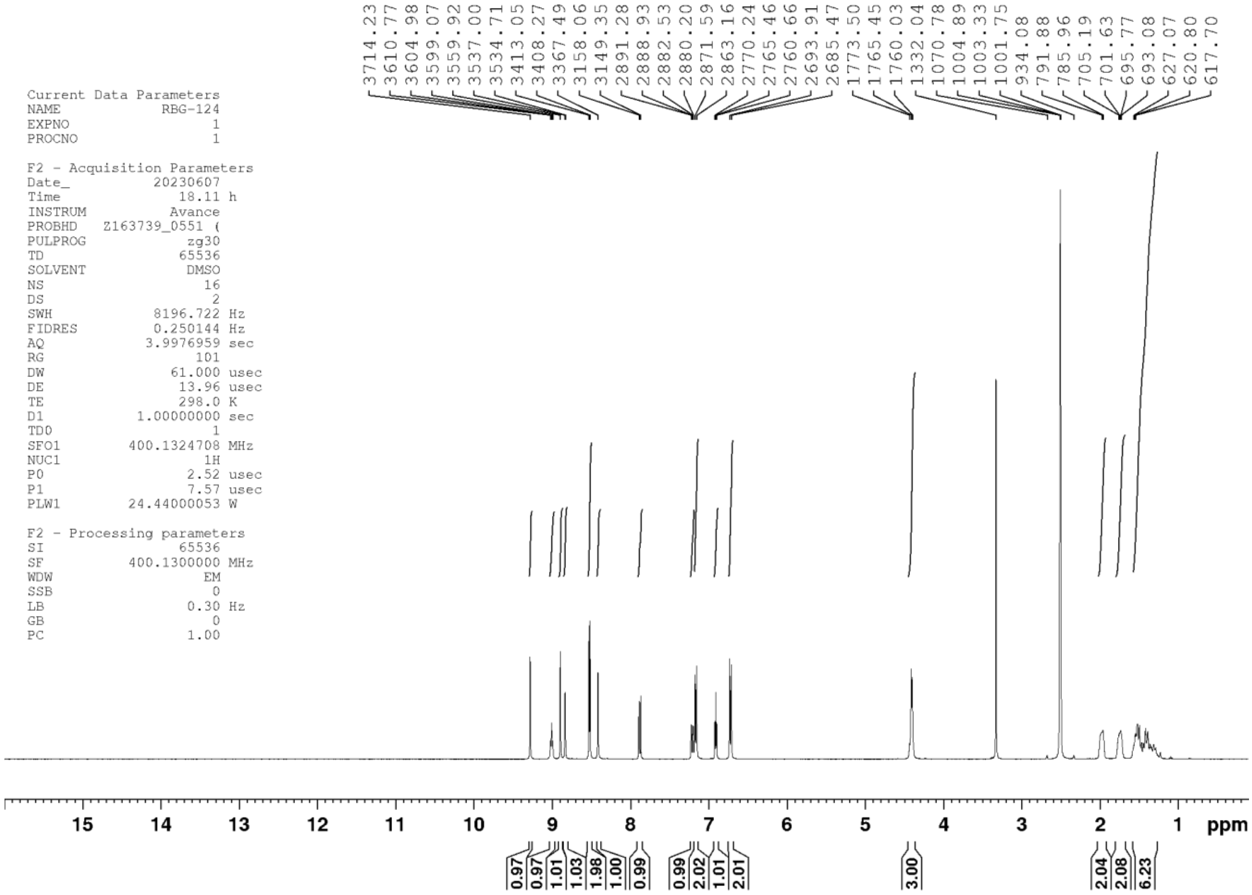

Figure S35. ^{1}H NMR spectrum of compound **17**.

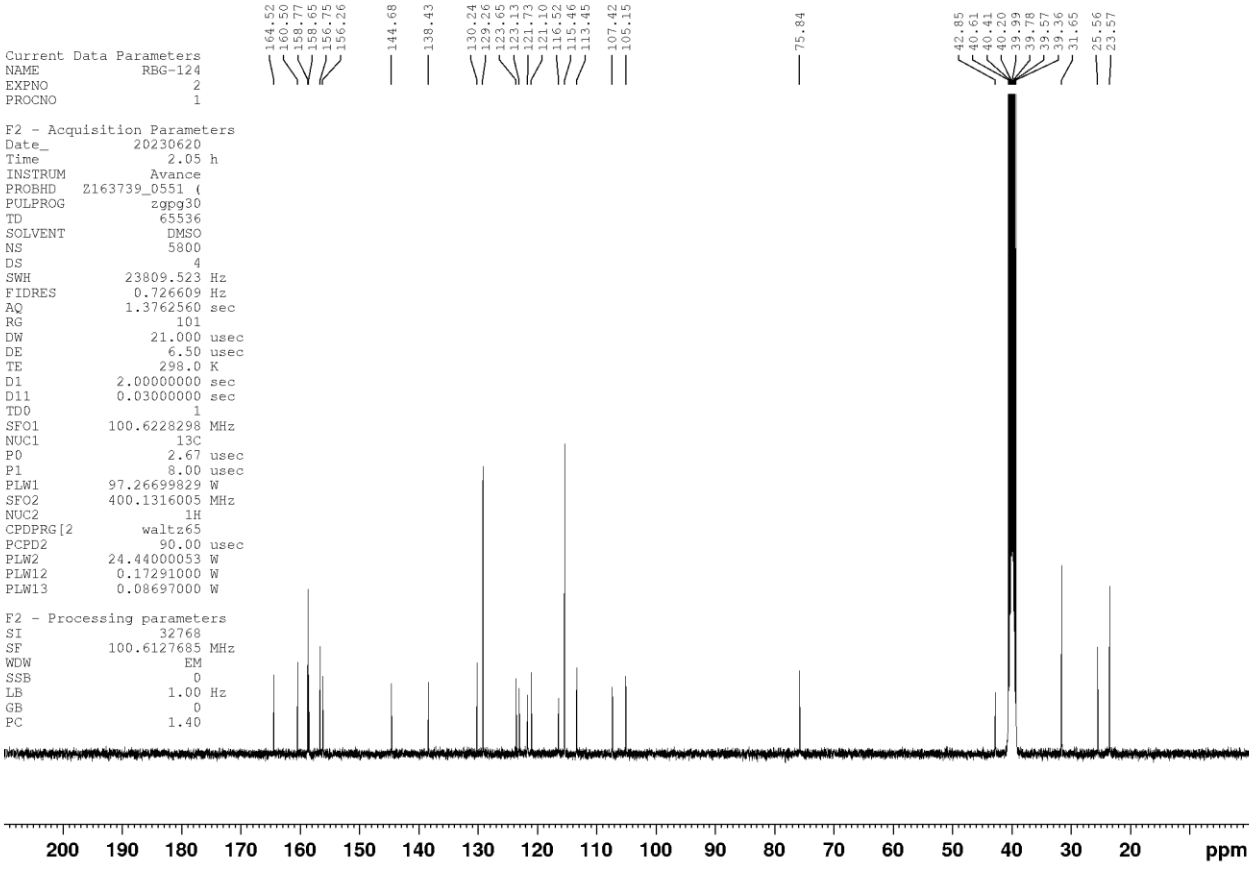

Figure S36. ^{13}C NMR spectrum of compound **17**.

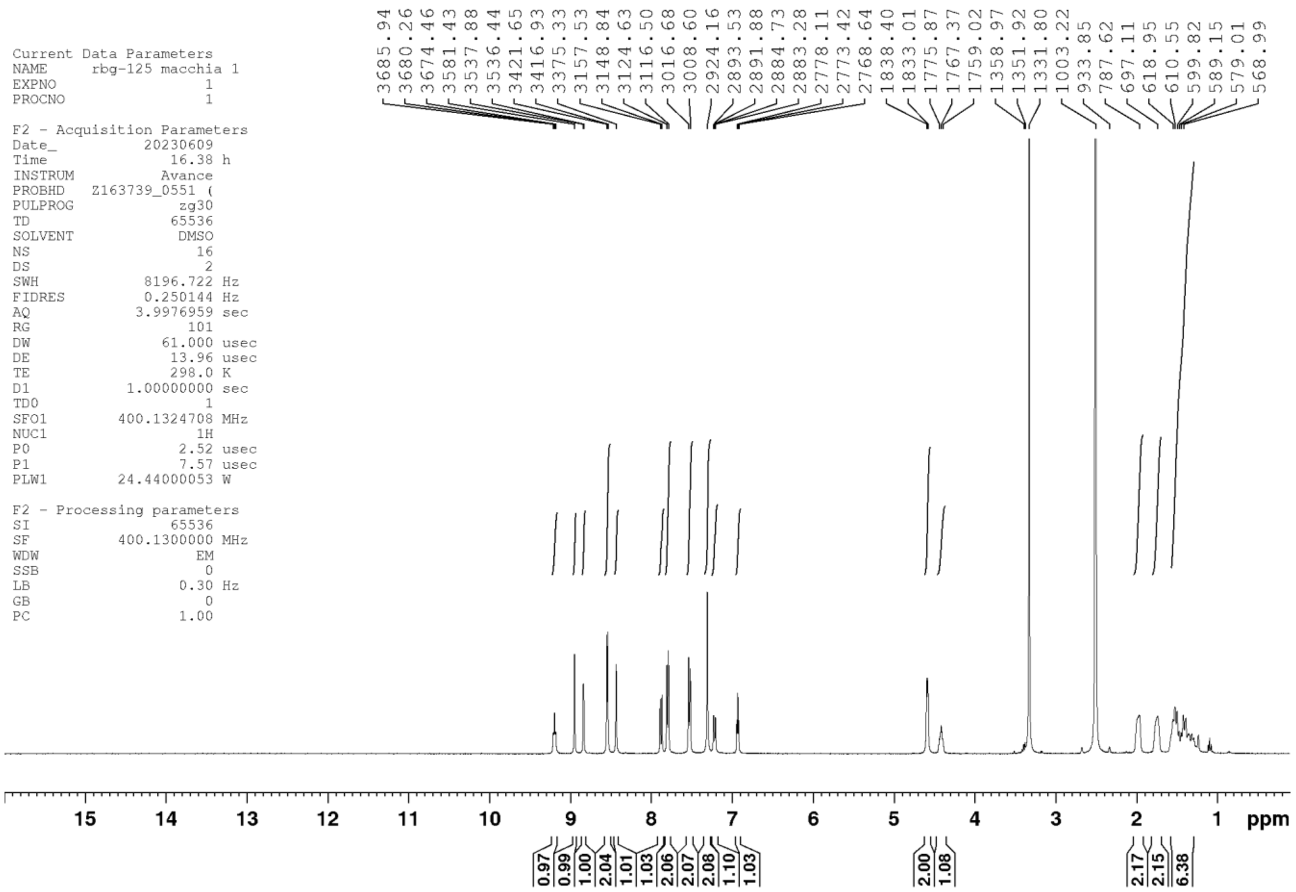

Figure S37. ^{1}H NMR spectrum of compound **18**.

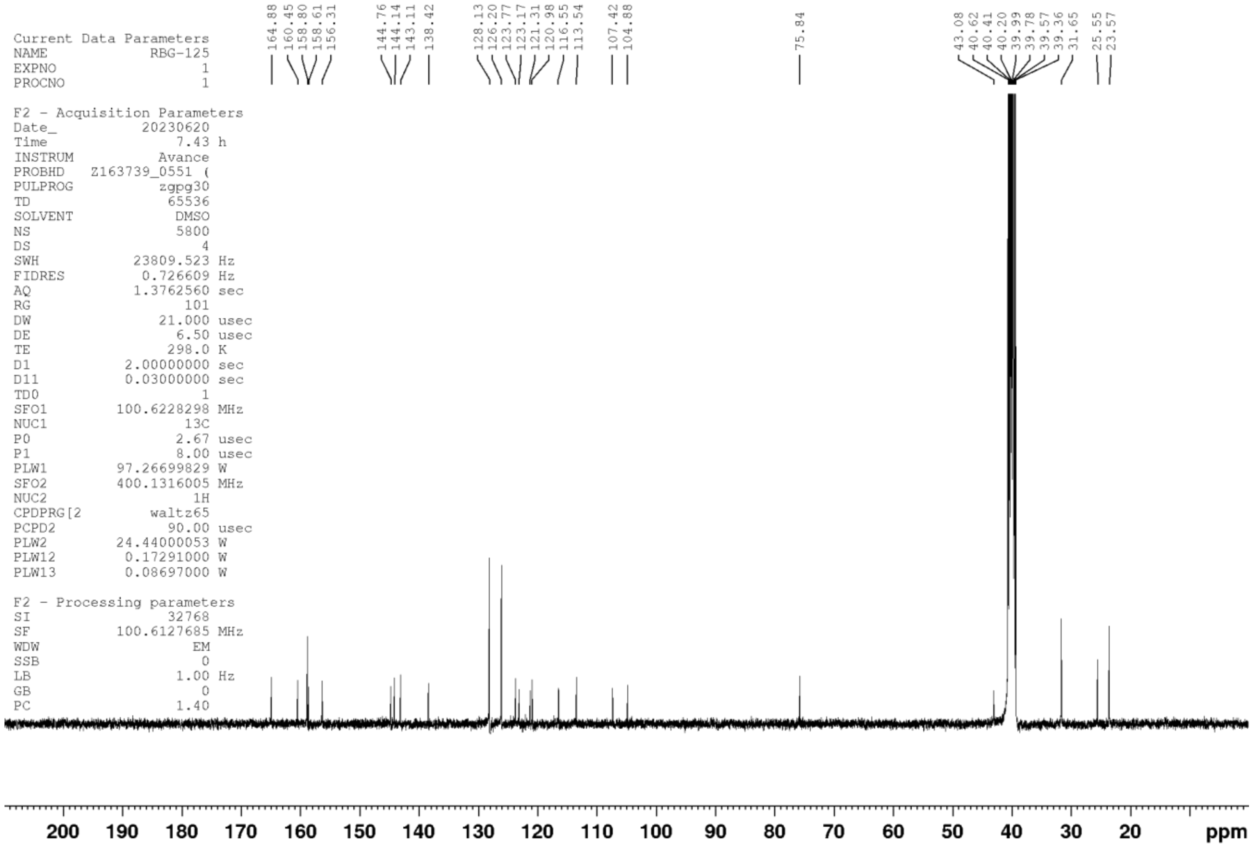

Figure S38. ^{13}C NMR spectrum of compound **18**.

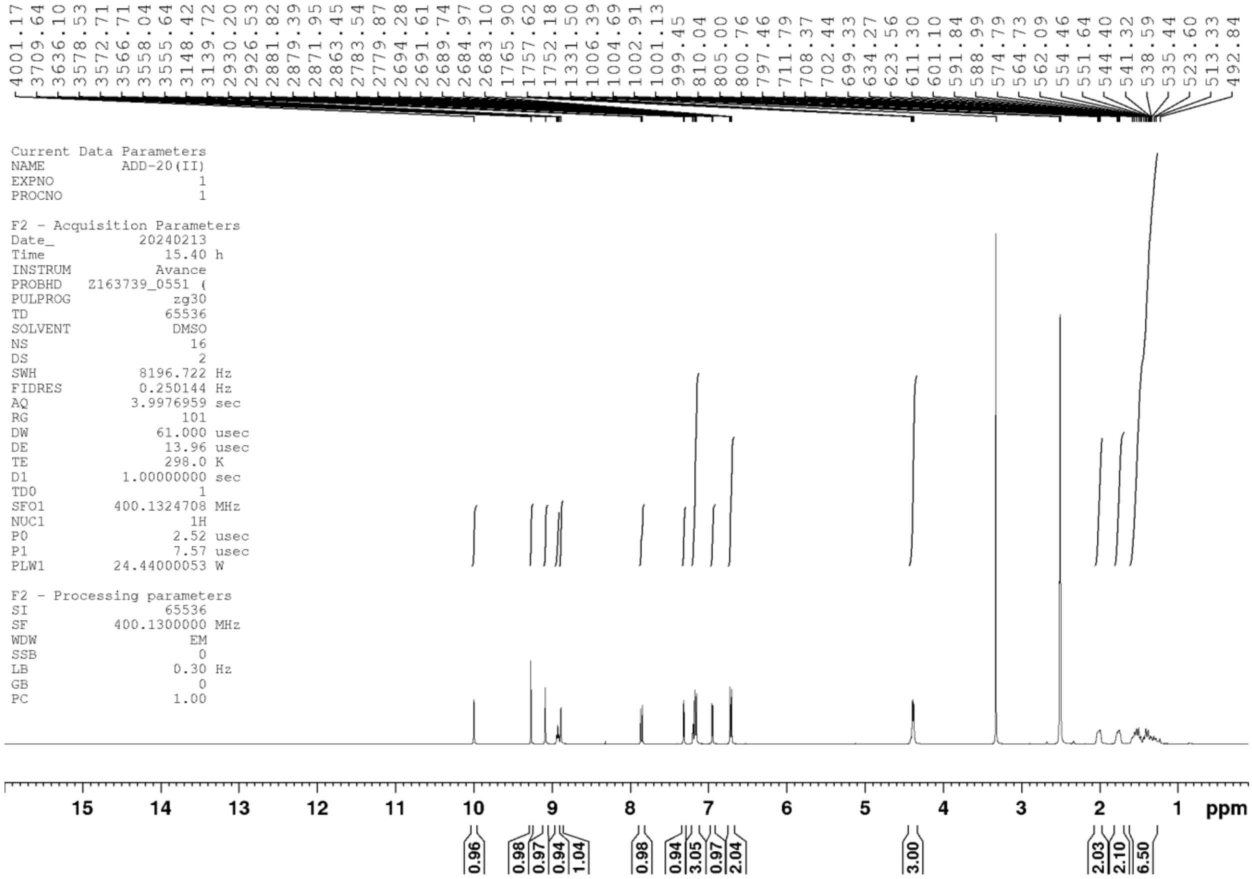

Figure S39. ^{1}H NMR spectrum of compound **19**.

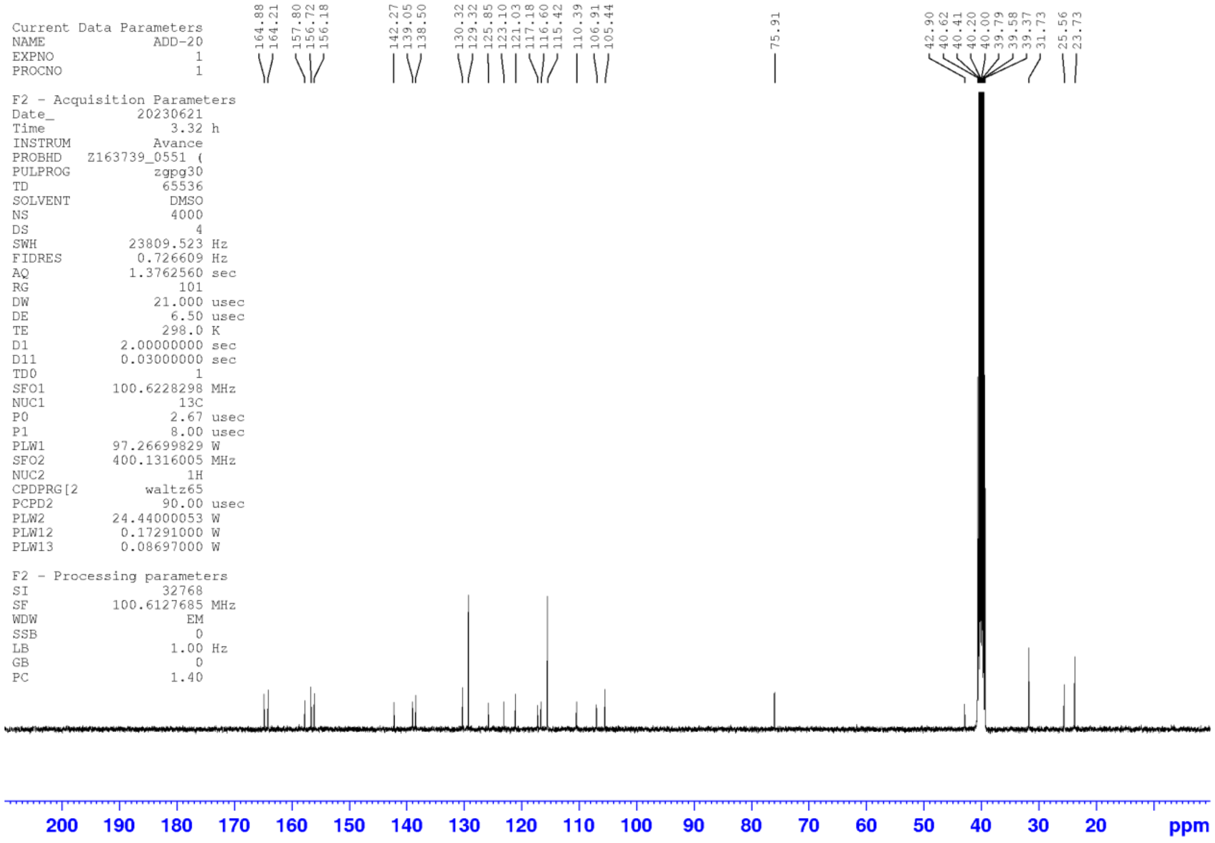

Figure S40. ^{13}C NMR spectrum of compound **19**.

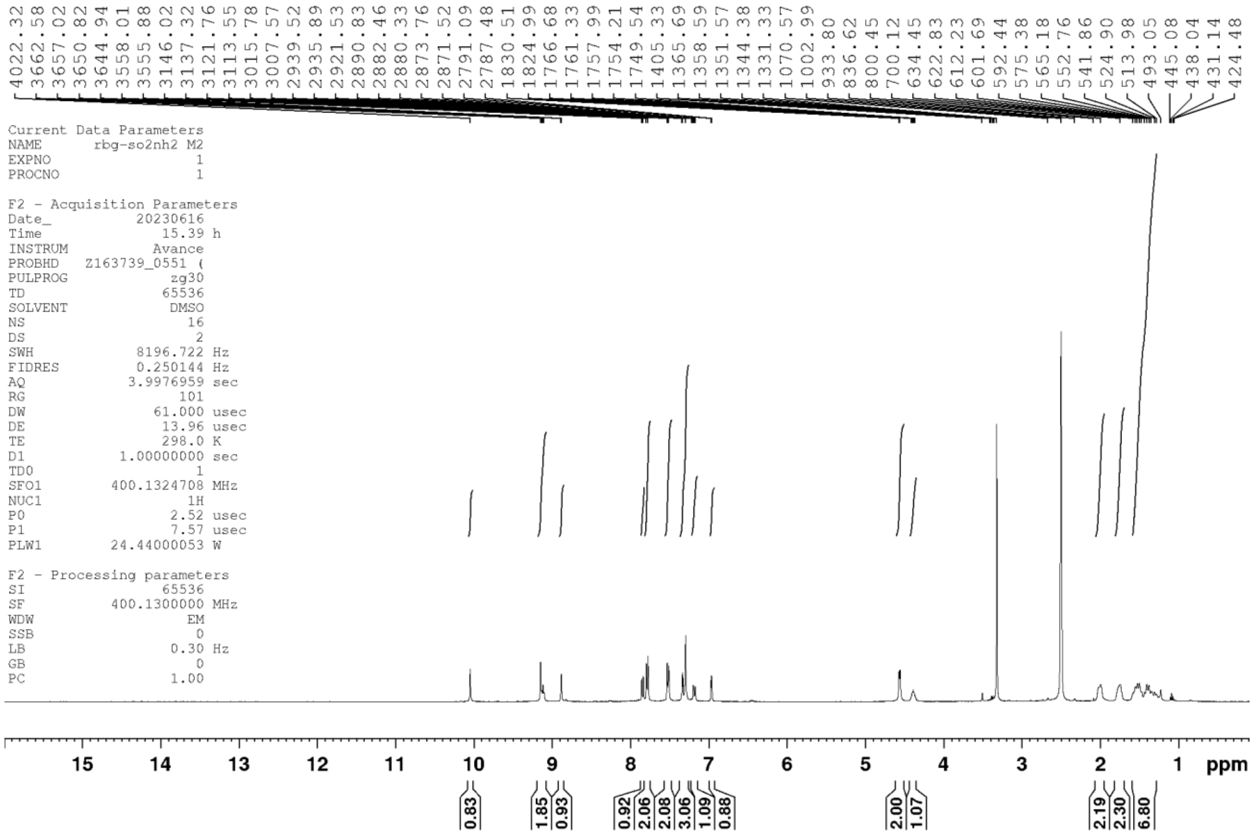

Figure S41. ^{1}H NMR spectrum of compound **20**.

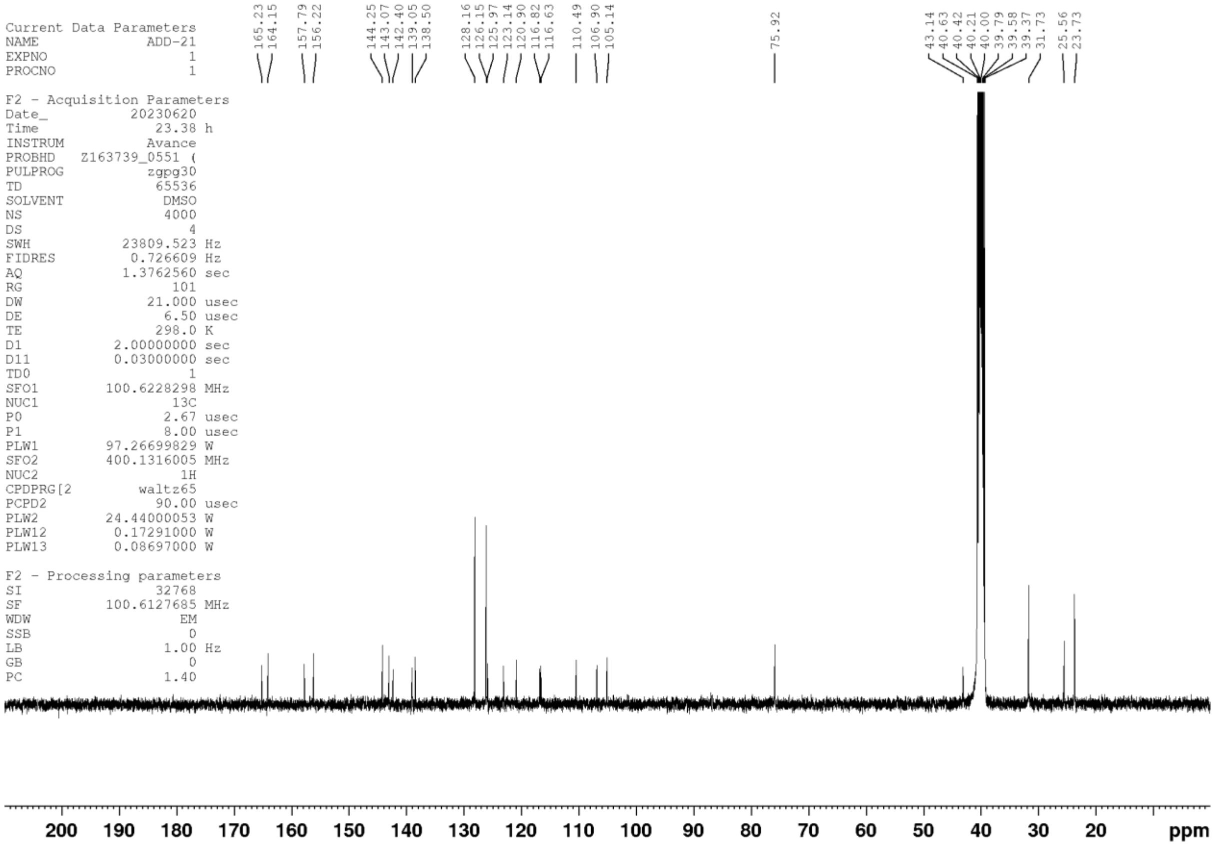

Figure S42. ^{13}C NMR spectrum of compound **20**.
